# Author Correction: Cross-ancestry genome-wide association analysis of corneal thickness strengthens link between complex and Mendelian eye diseases

**DOI:** 10.1038/s41467-018-07819-1

**Published:** 2019-01-08

**Authors:** Adriana I. Iglesias, Aniket Mishra, Veronique Vitart, Yelena Bykhovskaya, René Höhn, Henriët Springelkamp, Gabriel Cuellar-Partida, Puya Gharahkhani, Jessica N. Cooke Bailey, Colin E. Willoughby, Xiaohui Li, Seyhan Yazar, Abhishek Nag, Anthony P. Khawaja, Ozren Polašek, David Siscovick, Paul Mitchell, Yih Chung Tham, Jonathan L. Haines, Lisa S. Kearns, Caroline Hayward, Yuan Shi, Elisabeth M. van Leeuwen, Kent D. Taylor, Blue Mountains Eye Study - GWAS group, Jie Jin Wang, Elena Rochtchina, John Attia, Rodney Scott, Elizabeth G. Holliday, Tien Yin Wong, Paul N. Baird, Jing Xie, Michael Inouye, Ananth Viswanathan, Xueling Sim, Pieter Bonnemaijer, Jerome I. Rotter, Nicholas G. Martin, Tanja Zeller, Richard A. Mills, Emmanuelle Souzeau, Sandra E. Staffieri, Jost B. Jonas, Irene Schmidtmann, Thibaud Boutin, Jae H. Kang, Sionne E. M. Lucas, Tien Yin Wong, Manfred E. Beutel, James F. Wilson, Wellcome Trust Case Control Consortium 2 (WTCCC2), Peter Donnelly, Ines Barroso, Jenefer M. Blackwell, Elvira Bramon, Matthew A. Brown, Juan P. Casas, Aiden Corvin, Panos Deloukas, Audrey Duncanson, Janusz Jankowski, Hugh S. Markus, Christopher G. Mathew, Colin N. A. Palmer, Robert Plomin, Anna Rautanen, Stephen J. Sawcer, Richard C. Trembath, Nicholas W. Wood, Chris C. A. Spencer, Gavin Band, Céline Bellenguez, Colin Freeman, Garrett Hellenthal, Eleni Giannoulatou, Matti Pirinen, Richard Pearson, Amy Strange, Zhan Su, Damjan Vukcevic, Cordelia Langford, Sarah E. Hunt, Sarah Edkins, Rhian Gwilliam, Hannah Blackburn, Suzannah J. Bumpstead, Serge Dronov, Matthew Gillman, Emma Gray, Naomi Hammond, Alagurevathi Jayakumar, Owen T. McCann, Jennifer Liddle, Simon C. Potter, Radhi Ravindrarajah, Michelle Ricketts, Matthew Waller, Paul Weston, Sara Widaa, Pamela Whittaker, NEIGHBORHOOD consortium, R. Rand Allingham, Murray H. Brilliant, Donald L. Budenz, William G. Christen, John Fingert, David S. Friedman, Douglas Gaasterland, Terry Gaasterland, Michael A. Hauser, Peter Kraft, Richard K. Lee, Paul R. Lichter, Yutao Liu, Stephanie J. Loomis, Sayoko E. Moroi, Margaret A. Pericak-Vance, Anthony Realini, Julia E. Richards, Joel S. Schuman, William K. Scott, Kuldev Singh, Arthur J. Sit, Douglas Vollrath, Robert N. Weinreb, Gadi Wollstein, Donald J. Zack, Kang Zhang, André G. Uitterlinden, Eranga N. Vithana, Paul J. Foster, Pirro G. Hysi, Alex W. Hewitt, Chiea Chuen Khor, Louis R. Pasquale, Grant W. Montgomery, Caroline C. W. Klaver, Tin Aung, Norbert Pfeiffer, David A. Mackey, Christopher J. Hammond, Ching-Yu Cheng, Jamie E. Craig, Yaron S. Rabinowitz, Janey L. Wiggs, Kathryn P. Burdon, Cornelia M. van Duijn, Stuart MacGregor

**Affiliations:** 1 Department of Ophthalmology, Erasmus Medical Center, 3000 CA Rotterdam, The Netherlands; 2 Department of Epidemiology, Erasmus Medical Center, 3000 CA Rotterdam, The Netherlands; 3 Department of Clinical Genetics, Erasmus Medical Center, 3000 CA Rotterdam, The Netherlands; 4 University of Bordeaux, Bordeaux Population Health Research Center, INSERM UMR 1219, F-33000 Bordeaux, France; 5 Institute of Genetics and Molecular Medicine, Medical Research Council Human Genetics Unit, University of Edinburgh, Edinburgh, EH42XU UK; 6 Regenerative Medicine Institute and Department of Surgery, Cedars-Sinai Medical Center, Los Angeles, CA 90048 USA; 7 Cornea Genetic Eye Institute, Los Angeles, CA 90048 USA; 8 Department of Ophthalmology, University Medical Center Mainz, 55131 Mainz, Germany; 9 Department of Ophthalmology, Inselspital, University Hospital Bern, University of Bern, CH-3010 Bern, Switzerland; 10 Statistical Genetics, QIMR Berghofer Medical Research Institute, Brisbane, QLD 4029 Australia; 11 Department of Population and Quantitative Health Sciences, Case Western Reserve University, Cleveland, OH 44106 USA; 12 Institute for Computational Biology, Case Western Reserve University, Cleveland, OH 44106 USA; 13 Biomedical Sciences Research Institute, Ulster University, Belfast, Northern Ireland BT52 1SA UK; 14 Royal Victoria Hospital, Belfast Health and Social Care Trust, Belfast, Northern Ireland BT12 6BA UK; 15 Institute for Translational Genomics and Population Sciences and Department of Pediatrics, Los Angeles Biomedical Research Institute at Harbor-UCLA Medical Center, Torrance, CA 90509 USA; 16 Division of Genomic Outcomes, Departments of Pediatrics and Medicine, Harbor-UCLA Medical Center, Torrance, CA 90502 USA; 17 Centre for Ophthalmology and Visual Science, University of Western Australia, Lions Eye Institute, Perth, WA WA 6009 Australia; 18 Department of Twin Research and Genetic Epidemiology, King’s College London, London, WC2R 2LS UK; 19 Department of Public Health and Primary Care, Institute of Public Health, University of Cambridge School of Clinical Medicine, Cambridge, CB2 0SR UK; 20 NIHR Biomedical Research Centre, Moorfields Eye Hospital NHS Foundation Trust and UCL Institute of Ophthalmology, London, EC1V 9EL UK; 21 Faculty of Medicine, University of Split, HR-21000 Split, Croatia; 22 Departments of Medicine and Epidemiology and Cardiovascular Health Research Unit, University of Washington, Washington, WA 98101 USA; 23 The New York Academy of Medicine, New York, NY 10029 USA; 24 Centre for Vision Research, Department of Ophthalmology and Westmead Institute for Medical Research, University of Sydney, Sydney, NSW 2145 Australia; 25 Singapore Eye Research Institute, Singapore National Eye Centre, Singapore, 168751 Singapore; 26 Centre for Eye Research Australia, University of Melbourne, Royal Victorian Eye and Ear Hospital, East Melbourne, VIC 3002 Australia; 27 Department of Genetics and Computational Biology, QIMR Berghofer Medical Research Institute, Brisbane, QLD 4029 Australia; 28 Department of General and Interventional Cardiology, University Heart Center Hamburg, 20251 Hamburg, Germany; 29 German Center for Cardiovascular Research (DZHK), Partner Site Hamburg/Kiel/Lübeck, 20246 Hamburg, Germany; 30 Department of Ophthalmology, Flinders University, Adelaide, SA 5042 Australia; 31 Department of Ophthalmology, Medical Faculty Mannheim of the Ruprecht-Karls-University of Heidelberg, 68167 Mannheim, Germany; 32 Institute for Medical Biostatistics, Epidemiology and Informatics, University Medical Center Mainz, 55131 Mainz, Germany; 33 Channing Division of Network Medicine, Brigham and Women’s Hospital, Boston, MA 02115 USA; 34 Menzies Institute for Medical Research, University of Tasmania, Hobart, TAS 7005 Australia; 35 Ophthalmology & Visual Sciences Academic Clinical Program (Eye ACP), Duke-NUS Medical School, Singapore, 169857 Singapore; 36 Department of Ophthalmology, Yong Loo Lin School of Medicine, National University of Singapore, Singapore, 117549 Singapore; 37 Department of Psychosomatic Medicine and Psychotherapy, University Medical Center Mainz, Mainz, 55131 Germany; 38 Centre for Global Health Research, Usher Institute for Population Health Sciences and Informatics, University of Edinburgh, Edinburgh, EH16 4UX UK; 39 Department of Internal Medicine, Erasmus Medical Center, 3000 CA Rotterdam, The Netherlands; 40 Netherlands Consortium for Healthy Ageing, Netherlands Genomics Initiative, 2593 HW The Hague, The Netherlands; 41 School of Medicine, Menzies Institute for Medical Research, University of Tasmania, Hobart, TAS 7005 Australia; 42 Genome Institute of Singapore, 60 Biopolis Street, Singapore, 138672 Singapore; 43 Department of Ophthalmology, Harvard Medical School and Massachusetts Eye and Ear Infirmary, Boston, MA 02114 USA; 44 Institute for Molecular Bioscience, University of Queensland, Brisbane, QLD 4067 Australia; 45 Department of Ophthalmology, Radboud University Medical Center, 6525 GA Nijmegen, The Netherlands; 48 Department of Ophthalmology, Duke University Medical Center, Durham, NC 27705 USA; 49 Center for Human Genetics, Marshfield Clinic Research Foundation, Marshfield, WI USA; 50 Department of Ophthalmology, University of North Carolina, Chapel Hill, NC 27517 USA; 51 Department of Medicine, Brigham and Women’s Hospital, Boston, MA 02114 USA; 52 Department of Ophthalmology, College of Medicine, University of Iowa, Iowa City, IA 52242 USA; 53 Department of Anatomy/Cell Biology, College of Medicine, University of Iowa, Iowa City, IA 52242 USA; 54 Wilmer Eye Institute, John Hopkins University, Baltimore, MD 21287 USA; 55 Eye Doctors of Washington, Chevy Chase, MD 20815 USA; 56 Scripps Genome Center, University of California at San Diego, San Diego, CA 92037 USA; 57 Department of Medicine, Duke University Medical Center, Durham, NC 27710 USA; 58 Department of Biostatistics, Harvard School of Public Health, Boston, MA 02115 USA; 59 Bascom Palmer Eye Institute, University of Miami Miller School of Medicine, Miami, FL 33136 USA; 60 Department of Ophthalmology and Visual Sciences, University of Michigan, Ann Arbor, MI 48105 USA; 61 Institute for Human Genomics, University of Miami Miller School of Medicine, Miami, FL 33136 USA; 62 Department of Ophthalmology, WVU Eye Institute, Morgantown, WV 26506 USA; 63 Department of Ophthalmology, UPMC Eye Center, University of Pittsburgh, Pittsburgh, PA 15213 USA; 64 Department of Ophthalmology, Stanford University, Palo Alto, CA 94303 USA; 65 Department of Ophthalmology, Mayo Clinic, Rochester, MN 55905 USA; 66 Department of Genetics, Stanford University, Palo Alto, CA 94305 USA; 67 Department of Ophthalmology, Hamilton Eye Center, University of California, San Diego, CA 92093 USA; 68 Wellcome Trust Centre for Human Genetics, University of Oxford, Roosevelt Drive, Oxford, OX37BN UK; 69 Dept Statistics, University of Oxford, Oxford, OX1 3TG UK; 70 Wellcome Trust Sanger Institute, Wellcome Trust Genome Campus, Hinxton, Cambridge, CB10 1SA UK; 71 Telethon Institute for Child Health Research, Centre for Child Health Research, University of Western Australia, 100 Roberts Road, Subiaco, WA 6008 Australia; 72 Cambridge Institute for Medical Research, University of Cambridge School of Clinical Medicine, Cambridge, CB2 0XY UK; 73 Department of Psychosis Studies, NIHR Biomedical Research Centre for Mental Health at the Institute of Psychiatry, King’s College London and The South London and Maudsley NHS Foundation Trust, Denmark Hill, London, SE5 8AF UK; 74 University of Queensland Diamantina Institute, Brisbane, QLD 4102 Australia; 75 Dept Epidemiology and Population Health, London School of Hygiene and Tropical Medicine, London, WC1E 7HT UK; 76 Dept Epidemiology and Public Health, University College London, London, WC1E 6BT UK; 77 Neuropsychiatric Genetics Research Group, Institute of Molecular Medicine, Trinity College Dublin, Dublin 2, Eire, Ireland; 78 Molecular and Physiological Sciences, The Wellcome Trust, London, NW1 2BE UK; 79 Department of Oncology, Old Road Campus, University of Oxford, Oxford, OX3 7DQ UK; 80 Digestive Diseases Centre, Leicester Royal Infirmary, Leicester, LE7 7HH UK; 81 Centre for Digestive Diseases, Queen Mary University of London, London, E1 2AD UK; 82 Clinical Neurosciences, St George’s University of London, London, SW17 0RE UK; 83 King’s College London Dept Medical and Molecular Genetics, King’s Health Partners, Guy’s Hospital, London, SE1 9RT UK; 84 Biomedical Research Centre, Ninewells Hospital and Medical School, Dundee, DD1 9SY UK; 85 King’s College London Social, Genetic and Developmental Psychiatry Centre, Institute of Psychiatry, Denmark Hill, London, SE5 8AF UK; 86 University of Cambridge Dept Clinical Neurosciences, Addenbrooke’s Hospital, Cambridge, CB2 0QQ UK; 87 Dept Molecular Neuroscience, Institute of Neurology, Queen Square, London, WC1N 3BG UK; 46 University of Newcastle, Newcastle, NSW 2308 Australia; 47 Walter and Eliza Hall Institute of Medical Research, Melbourne, VIC 3052 Australia

Correction to: *Nature Communications* 10.1038/s41467-018-03646-6; published online 14 May 2018

Emmanuelle Souzeau, who contributed to analysis of data, was inadvertently omitted from the author list in the originally published version of this Article. This has now been corrected in both the PDF and HTML versions of the Article.

