# Supplementary material for: Cross-ancestry genome-wide association analysis of corneal thickness strengthens link between complex and Mendelian eye diseases
Source: Nat Commun. 2018 May 14;9:1864. doi: 10.1038/s41467-018-03646-6 (PMC5951816; doi:10.1038/s41467-018-03646-6)
Supplement: Supplementary file 1 — Supplementary Information [file 41467_2018_3646_MOESM1_ESM.pdf]

Cross-ancestry genome-wide association analysis of corneal thickness strengthens link between complex and Mendelian eye diseases

Supplementary Note 1

Keratoconus cohorts

Australian and Northern Ireland Keratoconus

Australian participants with keratoconus (n=711) were ascertained through the Department of Ophthalmology of Flinders Medical Centre, Adelaide, Australia; private optometry practices in Adelaide and Melbourne, Australia; the Royal Victorian Eye and Ear Hosptial, Melbourne, Australia; and by Australia-wide mail out to members of Keratoconus Australia, a community-based support group for patients. Patients from Northern Ireland (n=135) were recruited from the Ophthalmology Department of the Belfast Health and Social Care National Health System Trust. Clinical data were obtained from the participants‟ eye care practitioner, and patients were included in the study only if they met the recruitment criteria. The Blue Mountains Eye Study was used as the control cohort. This population study of individuals over the age of 50 years living in the Blue Mountains area west of Sydney undertook detailed ophthalmic evaluations of all participants. 2761 participants had genotyping information available. Details have described previously[1].

USA keratoconus study

Clinically affected Caucasian keratoconus cases (N=240) were enrolled into the GWAS as a part of the longitudinal videokeratography and genetic study at the Cornea Genetic Eye Institute[1,2]. After removing samples with poor quality of genotyping, 222 samples were included in the analysis. Caucasian controls (N=3,324) were obtained from the Cardiovascular Health Study (CHS), a population-based cohort study of risk factors for cardiovascular disease and stroke in adults 65 years of age or older, recruited at four field centers[3]. 5,201 predominantly Caucasian individuals were recruited in 1989-1990 from random samples of Medicare eligibility lists, followed by an additional 687 African-Americans recruited in 1992-1993 (total n= 5,888). CHS

was approved by the Institutional Review Board at each recruitment site, and subjects provided informed consent for the use of their genetic information. African-American CHS participants were excluded from analysis due to insufficient number of ethnically-matched cases. Genotyping details have been described previously[1]

Primary-open angle glaucoma cohorts

Australian & New Zealand Registry of Advanced Glaucoma

ANZRAG recruits cases of advanced glaucoma Australia-wide through ophthalmologist referral. The cohort also included participants enrolled in the Glaucoma Inheritance Study in Tasmania (GIST) who met the criteria for ANZRAG. This cohort has been described previously[4]. Advanced POAG was defined as best-corrected visual acuity worse than 6/60 due to POAG, or a reliable 24-2 Visual Field with a mean deviation of worse than -22db or at least 2 out of 4 central fixation squares affected with a Pattern Standard Deviation of < 0.5%. The less severely affected eye was also required to have signs of glaucomatous disc damage. Clinical exclusion criteria for this advanced POAG study were: i) pseudoexfoliation or pigmentary glaucoma, ii) angle closure or mixed mechanism glaucoma; iii) secondary glaucoma due to aphakia, rubella, rubeosis or inflammation; iv) infantile glaucoma, v) glaucoma in the presence of a known associated syndrome. The ANZRAG cohort included 1,155 ANZRAG glaucoma cases and 1,992 controls genotyped on Illumina Omni1M or OmniExpress arrays and imputed against 1000 Genomes Phase 1 Europeans. The case set included all samples from the previously published GWAS[4]. Controls were drawn from the Australian Cancer Study (225 oesophageal cancer cases, 317 Barrett's oesophagus cases and 552 controls) or from a study of inflammatory bowel diseases (303 cases and 595 controls). The quality control methods have been reported elsewhere[4].Imputation was conducted using IMPUTE2 in 1-Mb sections, with the 1000 Genomes phase 1 Europeans (March 2012 release) used as the reference panel. SNPs with imputation quality score >0.8 and MAF > 0.01 were carried forward for analysis. All were Australians of European ancestry. Approval was obtained from the Human Research Ethics Committees of Southern Adelaide Health Service/Flinders University, University of Tasmania, QIMR Berghofer Institute of Medical Research (Queensland Institute of Medical Research) and the Royal Victorian Eye and Ear Hospital.

NEIGHBORHOOD (National Eye Institute (NEI) Glaucoma Human Genetics Collaboration Heritable Overall Operational Database)

The NEIGHBORHOOD dataset is the meta-analysis of imputed GWAS summary data for 8 independent studies (total sample = 3,853 cases and 33,480 controls)[5]. The study was approved by the Massachusetts Eye and Ear Infirmary institutional review board and all subjects signed consent forms approved by the local IRB prior to enrolling in the study. For all enrollees, primary open angle glaucoma (POAG) cases were defined as individuals for whom reliable visual field (VF) tests show characteristic VF defects consistent with glaucomatous optic neuropathy. Individuals were classified as affected if the VF defects were reproduced on a subsequent test or if a single qualifying VF was accompanied by a cup-disc ratio (CDR) of 0.7 or more in at least one eye. For some cases VFs were not reliable and these cases had either CDR> 0.7 in both eyes are a difference in CDR of at least 0.2 between the eyes. In the OHTS study (one of the 8 NEIGHBORHOOD datasets) an alternative case definition based on progression of optic nerve degeneration was also used[6]. Patients with clinical features of secondary glaucoma based on the examination of the ocular anterior segment were excluded from this study. Elevation of IOP was not a criterion for inclusion; however, 67% of cases did have a history of elevated IOP (≥22 mm Hg) measured in a clinical setting (typically between the hours of 8AM and 5PM) and were classified as high-pressure glaucoma (HPG).

For all datasets genome-wide genotypes were obtained from either Illumina or Affymetrix platforms details have been described in detail elsewhere[5].

Supplementary Table 1. Descriptives of European and Asian studies included in the CCT Meta-analysis

Set 1. European CCT cohorts

Studies	Sample Size	Labmda	Mean CCT	SD CCT	Range CCT	Mean Age	SD Age	Range Age	Sex (% male)	Genotyping platform	Imputation platform	Measurement method	PMID for1000G imputation
BATS	1153	1.0050	544.3	35.00	381:679	21.4	12.6	5;90	56%	Illumina Human660W-Quad	MACH/minimac	Ultrasound pachymetry, Tomey SP 2000	25637523
BMES	1048	1.0098	539.8	33.38	441.1; 662.2	62.76	7.56	49;86	57.73%	Illumina Human660W-Quad	IMPUTE2	Ultrasound pachymetry device	25637523
CROATIA-Korcula	848	0.9324	558.0	36.72	468; 779.5	56.36	13.70	18;98	35.14%	Illumina 370CNV-Quad v1	IMPUTE2	Nidek Echoscan US-1800 A-scan	28073927
CROATIA-Split	782	1.0017	559.2	35.40	445.5; 663.5	50.31	14.39	18;85	39.45%	Illumina 370CNV-Quad v1	IMPUTE2	Nidek Echoscan US-1800 A-scan	28073927
CROATIA-Vis	591	0.9549	560.9	34.20	441; 661.5	56.71	13.52	25;86	40.00%	Illumina 370CNV-Quad v1	IMPUTE2	NIDEK Echoscan US-1800 A-scan	28073927
EPIC	184	1.0010	558.5	39.60	454; 662	72.00	7.83	52;88	47.83%	Affymetrix GeneChip Human Mapping 500K Array Set	IMPUTE2	Ultrasound pachymetry (Pachmate DGH 55, mean of 10 readings per eye; DGH Technology, Exton, PA)	28073927
GHS1	2727	1.0246	551.4	34.60	442; 676	55.90	10.9	35;74	51.40%	Affymetrix Genome-Wide Human SNP 6.0 Array	MACH version 1.0.18.c	Pachycam, Oculus, Wetzlar, Germany	28073927
GHS2	1118	1.0169	562	34	412; 675	55.00	10.8	35;74	49.70%	Affymetrix Genome-Wide Human SNP 6.0 Array	MACH version 1.0.18.c	Pachycam, Oculus, Wetzlar, Germany	28073927
Orcades	1096	1.0058	538	32.74	431.5; 663.0	55.09	14.3	18.11;89.21	37.59%	Illumina 370CNV-Quad v1	IMPUTE2	NIDEK Echoscan US-1800 A-scan	28073927

Studies	Sample Size	Labmda	Mean CCT	SD CCT	Range CCT	Mean Age	SD Age	Range Age	Sex (% male)	Genotyping platform	Imputation platform	Measurement method	PMID for1000G imputation
RS1	873	0.9891	544.5	34.47	431.7; 678.5	75.75	7.10	56.5;96.26	50.76%	Illumina 550K	MACH/minimac	Non-contact biometer Lenstar LS900, Haag-Streit, Köniz, Switerzerland	25637523
RS2	1215	1.0080	547.6	34.18	443.0; 669.0	72.58	5.30	65.87;98.59	46.50%	Illumina 550K	MACH/minimac	Non-contact biometer Lenstar LS900, Haag-Streit, Köniz, Switerzerland	25637523
RS3	2391	0.9950	550.8	33.70	443.5; 674.0	61.53	5.10	51.53;89.87	43.44%	Illumina 610K and 660K	MACH/minimac	Non-contact biometer Lenstar LS900, Haag-Streit, Köniz, Switerzerland	25637523
TEST	686	0.9857	544.3	35.00	381:679	21.4	12.6	5;90	56%	Illumina Human660W-Quad	MACH/minimac	Ultrasound pachymetry, Tomey SP 2000	25637523
TwinsUK	2080	1.2400	546.47	33.5	430.3; 657.5	56.71	11.6	16.11 - 83.26	1.78%	Illumina 610K, Illumina 317K	IMPUTE2	Ultrasound pachymetry device	28073927
Raine	1011	1.0127	537.90	32.26	417;647	17	1	18-22	48.8	Illumina 610K	MACH/minimac	Pachycam, Oculus, Wetzlar, Germany	25637523
**Total**	**17803**												

Set 2. Asian CCT cohorts

Studies	Sample Size	Lambda	Mean CCT	SD CCT	Range CCT	Mean Age	SD Age	Range Age	Sex (% male)	Genotyping platform	Imputation platform	Measurement method	PMID for1000G imputation
BES	620	1.005	536.5	34.70	416;644	45.23	31.77	9;90	35.37%	610	Minimac		28073927
SCES	1861	1.006	552.7	32.80	402;684.5	58.51	9.50	44.47;84.92	51.16%	610	Minimac	ultrasonic pachymetry	28073927
SCES2	608	1.028	552.0	32.91	442;657	60.37	9.53	46.28;83.67	51.32%	OmniExpress	Minimac		28073927
SiMES	2510	1.042	540.3	32.90	424.5; 662.5	59.56	11.02	40.11;80.93	49.56%	610	Minimac	ultrasonic pachymetry	28073927
SINDI	2508	1.039	539.5	33.25	418.5; 653.5	57.98	9.98	42.67;84.13	51.36%	610	Minimac	ultrasonic pachymetry	28073927
Total	8107												

Supplementary Table 2. CCT associated loci from Meta-analysis of European studies (n = 17,803) and look up in Asian Meta-analysis (n = 8,107

					European-specific Meta-analysis			Asian-specific Meta-analysis		
**MarkerID**	**Chr:BP_hg19**	**CytoLocation**	**NearestGene***	**EA/OA**	**Freq EA Eur**	**β_Euro (s.e.)**	**p_EURO**	**Freq EA ASN**	**β_ASN (s.e.)**	**p_ASN**
**rs115781177**	**2:33348494**	**2p22-p21**	***LTBP1***	**a/g**	**0.930**	**-5.0453 (0.8944)**	**1.69E-08**	**NA**	**NA**	**NA**
rs121908120	2:219755011	2q35	*WNT10A*	a/t	0.029	-11.4832 (1.5894)	5.02E-13	NA	NA	NA
rs62279163	2:228168099	2q36-q37	*COL4A3*	a/c	0.347	-2.5123 (0.3959)	2.21E-10	0.3495	-2.0039 (0.5445)	2.33E-04
**rs13024279**	**2:235472595**	**2q37.1**	***ARL4C***	**a/g**	**0.436**	**-2.282 (0.3963)**	**8.52E-09**	**0.6837**	**-0.0641 (0.5811)**	**9.12E-01**
**3:136138073**	**3:136138073**	**3q22.3**	***STAG1***	**d/r**	**0.235**	**-2.6465 (0.4748)**	**2.49E-08**	**0.1889**	**-2.8081 (1.0968)**	**1.05E-02**
rs9822953	3:156472071	3q25.31	*TIPARP*	t/c	0.670	2.6943 (0.404)	2.57E-11	0.6667	1.1125 (0.6173)	7.15E-02
rs7617946	3:171936825	3q26.31	*FNDC3B*	t/c	0.732	3.1864 (0.4251)	6.59E-14	0.5402	2.326 (0.5198)	7.66E-06
3:177298094	3:177298094	3q26.32	*TBL1XR1*	i/r	0.382	-2.4318 (0.4219)	8.20E-09	0.4918	-1.6662 (0.5335)	1.79E-03
rs10064391	5:64686659	5q12.3	*CWC27*	a/g	0.629	-2.7649 (0.3967)	3.18E-12	0.7021	-0.8887 (0.601)	1.39E-01
rs1931656	6:82610188	6q14.1	*FAM46A*	a/t	0.451	2.1722 (0.3909)	2.75E-08	0.4717	2.9646 (0.5293)	2.13E-08
rs68168107	7:66255238	7q11.21	*RABGEF1*	a/g	0.265	-3.3069 (0.4459)	1.21E-13	0.3392	-2.7037 (0.556)	1.16E-06
**rs10429294**	**8:95969322**	**8q22.1**	***NDUFAF6***	**t/c**	**0.504**	**2.3608 (0.3947)**	**2.21E-09**	**0.6643**	**1.3474 (0.5595)**	**1.60E-02**
rs10817107	9:113656170	9q31.3	*LPAR1*	a/c	0.214	2.6501 (0.4603)	8.55E-09	0.2102	3.3713 (0.6558)	2.74E-07
rs1536482	9:137440528	9q34.2	*COL5A1*	a/g	0.337	-4.5691 (0.3985)	1.95E-30	0.3248	-2.8641 (0.6005)	1.85E-06
rs2386136	9:139864341	9q34.2	*PRR31*	a/g	0.495	3.7247 (0.4433)	4.40E-17	NA	NA	NA
rs35809595	10:63831928	10q21.2	*ARID5B*	a/g	0.405	-2.2981 (0.3997)	8.97E-09	0.3641	-2.6693 (0.5367)	6.56E-07
**rs56009602**	**11:130289612**	**11q24.3**	***ADAMTS8***	**t/c**	**0.050**	**6.8655 (0.927)**	**1.30E-13**	**0.1007**	**7.2477 (0.9399)**	**1.25E-14**
**rs7308752**	**12:91527181**	**12q21.33**	***DCN***	**a/g**	**0.910**	**3.8747 (0.6775)**	**1.07E-08**	**0.726**	**2.2886 (0.6819)**	**7.91E-04**
rs11111869	12:104402485	12q23.3	*GLT8D2*	a/g	0.170	3.1744 (0.5098)	4.77E-10	0.2248	3.4794 (0.6356)	4.38E-08

					European-specific Meta-analysis			Asian-specific Meta-analysis		
**MarkerID**	**Chr:BP_hg19**	**CytoLocation**	**NearestGene***	**EA/ OA**	**Freq EA Eur**	**β_Euro (s.e.)**	**p_EURO**	**Freq EA ASN**	**β_ASN (s.e.)**	**p_ASN**
rs7327928	13:23228314	13q12.11	*FGF9-SGCG*	t/c	0.684	-2.4174 (0.4055)	2.50E-09	0.6456	-1.1019 (0.5665)	5.18E-02
rs2755238	13:41110270	13q14.11	*FOXO1*	t/c	0.899	5.8302 (0.6265)	1.32E-20	NA	NA	NA
rs785420	15:30196116	15q13.1	*TJP1*	a/t	0.893	4.0816 (0.6409)	1.91E-10	0.9168	3.0358 (1.2091)	1.21E-02
rs12912010	15:67467143	15q22.33	*SMAD3*	t/g	0.221	2.7636 (0.476)	6.40E-09	0.359	2.2196 (0.5397)	3.92E-05
rs34896088	15:85821624	15q25.3	*AKAP13*	a/c	0.791	3.4329 (0.4775)	6.52E-13	0.6461	1.9599 (0.5645)	5.17E-04
rs930847	15:101558562	15q26.3	*LRRK1*	t/g	0.770	-3.5726 (0.4532)	3.19E-15	0.7309	-3.793 (0.6169)	7.82E-10
rs35193497	16:88324821	16q24.2	*ZNF469*	t/g	0.358	-6.2382 (0.4343)	8.64E-47	0.2886	-4.928 (0.6221)	2.34E-15
**17:7423081**	**17:7423081**	**17p13.1**	***POLR2A***	**d/r**	**0.624**	**-2.4139 (0.4293)**	**1.87E-08**	**0.5294**	**-0.0501 (0.5358)**	**9.25E-01**
rs9900807	17:14564143	17p12	*HS3ST3B1*	t/c	0.286	-2.4969 (0.4167)	2.08E-09	0.4766	-1.9066 (0.5219)	2.59E-04

EA; effect allele, OA; other allele, Freq EA Eur, Frequency of the effect allele in European (EUR) or Asian (ASN) population. β; effect size of the effect allele in European (EUR) or Asian (ASN) population, p; p-value.

*Nearest gene (reference NCBI build37) is given as locus label, but this should not be interpreted as providing support that the nearest gene is the best candidate, a list including all the genes +/- 200kb of the lead SNP is presented in **Supplementary Table 12**

NA= SNPs that did not meet our filtering criteria in the Asian-specific meta-analysis (i.e., minor allele frequency (MAF) > 0.01, Info > 0.3 and available in at least two cohorts).

Supplementary Table 3. CCT associated loci from Meta-analysis of Asian studies and look up in European-specific Meta-analysis

					Asian-specific Meta-analysis			European-specific Meta-analysis		
**MarkerID**	**Chr:BP_hg19**	**CytoLocation**	**NearestGene**	**EA/OA**	**Freq_EA_ASN**	**β (s.e.) ASN**	**p_ASN**	**Freq_EA_Eur**	**β (s.e.) EUR**	**p_EURO**
rs96067	1:36571920	1p34.3	*COL8A2*	a/g	0.5609	3.9449 (0.5205)	3.48E-14	0.8063	0.9964(0.4871)	4.08E-02
rs6445046	3:171933252	3q26.31	*FNDC3B*	t/g	0.6609	3.1726 (0.5758)	3.59E-08	0.7835	3.7388(0.4996)	7.22E-14
rs1538138	6:82794594	6q14.1	*FAM46A*	t/c	0.2379	-3.5148 (0.6138)	1.03E-08	0.2464	-2.1104(0.449)	2.60E-06
rs6971897	7:66412774	7q11.21	*RABGEF1*	a/t	0.6321	-3.3593 (0.5967)	1.80E-08	0.3649	-2.3597(0.4343)	5.53E-08
rs13290289	9:13563660	9p23	*MPDZ*	t/g	0.2455	-3.8698 (0.6012)	1.22E-10	0.1835	-1.7069(0.4926)	5.30E-04
9:113718662	9:113718662	9q31.3	*LPAR1*	d/r	0.211	3.8266 (0.6619)	7.41E-09	0.2865	2.1025(0.4384)	1.62E-06
rs3132309	9:137433436	9q34.3	*COL5A1*	a/c	0.4486	-4.8903 (0.5646)	4.63E-18	0.4139	-4.0865(0.4299)	1.97E-21
rs16920206	10:66485072	10q21.3	*REEP3*	t/c	0.9397	-9.4663 (1.6472)	9.09E-09	0.972	2.3196(1.4794)	1.17E-01
rs56063990	11:130293150	11q24.3	*ADAMTS8*	t/c	0.9038	-7.6327 (0.9858)	9.72E-15	0.9565	-6.8984(1.0244)	1.65E-11
rs11553764	12:104415244	12q23.3	*GLT8D2*	t/c	0.2036	4.1453 (0.6759)	8.62E-10	0.1693	3.19(0.5366)	2.77E-09
rs1828481	15:85840912	15q25.3	*AKAP13*	a/c	0.5231	-3.0994 (0.5366)	7.66E-09	0.6127	-2.0371(0.3855)	1.26E-07
rs4965359	15:101585336	15q26.3	*LRRK1*	a/g	0.4997	-3.2801 (0.5292)	5.69E-10	0.3896	-1.808(0.3899)	3.54E-06
rs12448211	16:88330513	16q24.2	*ZNF469*	a/g	0.6913	4.7832 (0.5911)	5.86E-16	0.6232	5.8807(0.417)	3.63E-45

EA; effect allele, OA; other allele, Freq EA Eur, Frequency of the effect allele in European (EUR) or Asian (ASN) population. β; effect size of the effect allele in European (EUR) or Asian (ASN) population, p; p-value.

*Nearest gene (reference NCBI build37) is given as locus label, but this should not be interpreted as providing support that the nearest gene is the best candidate, a list including all the genes +/- 200kb of the lead SNP is presented in **Supplementary Table 12**

Supplementary Table 4. Follow-up of CCT associated loci in Keratoconus cohorts (n = 933 cases / n= 5,946 controls)

MarkerID	Chr:BP_hg19	Cyto Loci	Sentinel_CCT_variant	Nearest_gene	EA	OR	SE	P	expected risk effect direction‡
rs96067	1:36571920	1p34.2	rs96067	*COL8A2*	G	1.170	0.073	3.12E-02	+
**rs4846476**	**1:218526228**	**1q41**	**rs4846476**	***TGFB2***	**C**	**1.087**	**0.071**	**2.34E-01**	**+**
**rs115781177**	**2:33348494**	2p22-p21	**rs115781177**	***LTBP1***	**G**	**0.720**	**0.129**	**1.06E-02**	**+**
rs4608502	2:228134155	2q36-q37	rs4608502	*COL4A3*	T	1.210	0.062	2.11E-03	+
**rs28641809***	**3:136290489**	3q22.3	**3:136138073**	***STAG1***	**A**	**1.187**	**0.069**	**1.35E-02**	**+**
rs4857612*	3:177306621	3q26.32	3:177306757	*TBL1XR1*[a]	C	1.015	0.060	8.07E-01	+
rs17024437*	4:149081808	4q31.1	rs28789690	*NR3C2*	A	1.268	0.106	2.45E-02	+
rs1309531*	5:64306311	5q12.3	rs10471310	*ADAMTS6*	T	0.935	0.061	2.71E-01	+
**rs11743204***	**5:178671014**	**5q35.3**	**rs35028368**	***ADAMTS2***	**C**	**1.070**	**0.063**	**2.87E-01**	**+**
**rs13191376**	**6:45522139**	**6p21.1**	**rs13191376**	***RUNX2***	**T**	**0.941**	**0.063**	**3.30E-01**	**-**
**rs1412710**	**6:75837203**	**6q13**	**rs1412710**	***COL12A1***	**T**	**0.782**	**0.089**	**5.63E-03**	**-**
rs1931656	6:82610188	6q14.1	rs1931656	*FAM46A*	A	1.015	0.060	8.05E-01	-
**rs9455877***	**6:169556637**	6q27	**6:169553553**	***THBS2***	**G**	**1.205**	**0.079**	**1.84E-02**	**+**
rs3800817*	7:66263550	7q11.21	7:66262284	*RABGEF1*[b]	A	1.177	0.067	1.53E-02	+
**rs2106166**	**7:92668332**	**7q21.2**	**rs2106166**	***SAMD9***	**T**	**1.082**	**0.060**	**1.89E-01**	**+**
**rs3808520**	**8:23164773**	**8p21.3**	**rs3808520**	***LOXL2***	**C**	**0.895**	**0.075**	**1.40E-01**	**+**
rs66720556	9:13559717	9p23	rs66720556	*MPDZ*	A	1.437	0.074	1.02E-06	+
**rs7026684**	**9:4215308**	**9p24.2**	**rs7026684**	***GLIS3***	**A**	**0.957**	**0.063**	**4.79E-01**	**-**
rs3132303	9:137444298	9q34.2	rs3132303	*COL5A1*	C	0.704	0.074	2.21E-06	+
rs2386136*	9:139864341	9q34.3	rs7040970	*LCN12*	A	0.918	0.064	1.84E-01	+
rs35809595	10:63831928	10q21.2	rs35809595	*ARID5B*	A	1.154	0.061	1.99E-02	+
**rs2419835**	**10:115296564**	**10q25.3**	**rs2419835**	***HABP2***	**C**	**1.140**	**0.086**	**1.26E-01**	**+**

MarkerID	Chr:BP_hg19	Cyto Loci	Sentinel_CCT_variant	Nearest_gene	EA	OR	SE	P	expected risk effect direction‡
rs4938174	11:110913240	11q23.1	rs4938174	*ARHGAP20-C11orf53*	A	0.979	0.066	7.51E-01	+
**rs7308752**	**12:91527181**	12q21.33	**rs7308752**	***DCN***	**G**	**1.317**	**0.101**	**6.33E-03**	**+**
rs11553764	12:104415244	12q23.3	rs11553764	*GLT8D2*	T	0.968	0.078	6.80E-01	+
rs2755238*	13:41110270	13q14.11	13:41112152	*FOXO1*	C	1.548	0.089	8.90E-07	+
**rs56223983**	**14:81814754**	**14q31.1**	**rs56223983**	***STON2***	**T**	**0.987**	**0.064**	**8.39E-01**	**+**
rs62014489*	15:30171879	15q13.1	rs785422	*TJP1*	A	0.873	0.099	1.72E-01	-
**rs8030753**	**15:48801935**	**15q21.1**	**rs8030753**	***FBN1***	**T**	**1.035**	**0.087**	**6.94E-01**	**-**
rs12912010	15:67467143	15q22.33	rs12912010	*SMAD3*	T	0.779	0.078	1.43E-03	+
rs4843040	15:85838636	15q25.3	rs4843040	*AKAP13*[c]	T	1.196	0.070	1.08E-02	+
rs930847	15:101558562	15q26.3	rs930847	*LRRK1*	G	0.951	0.073	4.95E-01	+
rs35193497	16:88324821	16q24.2	rs35193497	*ZNF469*	T	0.844	0.065	9.47E-03	-
rs4792535	17:14565130	17p12	rs4792535	*HS3ST3B1*	T	0.990	0.067	8.84E-01	-
rs11656734*	17:7426695	17p13.1	17:7423081	*POLR2Ad*	C	0.844	0.063	6.91E-03	+
**rs71313932***	**22:19960198**	**22q11.21**	**rs8133436**	***ARVCF***	**C**	**0.983**	**0.068**	**7.96E-01**	**+**

Results from the look-up in the keratoconus meta-analysis (933 cases and 5,946 controls of European ancestry). MarkerID, rsID; Chr:bp_hg19, chromosome: base pair NCBI build37, Cyto loci, cytogenic location; Sentinel_CCT_variant, CCT lead SNP; Nearest gene (reference NCBI build37) is given as locus label, but this should not be interpreted as providing support that the nearest gene is the best candidate, a list including all the genes +/- 200kb of the lead SNP is presented in Supplementary Table 12;EA; Effect Allele, OR; Odd ratio, SE, standard error of the odds ratio; P, P-value.

* In LD r2>0.8 with Lead SNP
[a]. The lead SNP is located in a validated non-coding mRNA, *LINC00578*; [b] In Lu et al this locus was reported as two loci (VKORC1L1 and C7orf42); [c] The lead SNP is located in the pseudogene *ADAMTS7P4*
‡ variants in which the CCT-reducing allele also increases the risk of keratoconus were labelled with positive sign (+), variants in which this condition was not fulfilled were labelled with negative sign (-)
Novel CCT-loci are shown in **bold**

Supplementary Table 5. Follow-up of CCT associated loci in POAG cohorts

					Meta-analysis of POAG cohorts				ANZRAG cohort		NEIGHBORHOOD Consortium	
**MarkerID**	**Chr:BP_hg19**	**CytoLocation**	**NearestGene**	**EA/OA**	**β (s.e.)**	**OR†**	**P-value**	**Expected effect direction‡**	**β (s.e.)**	**P-value**	**β (s.e)**	**P-value**
rs96067	1:36571920	1p34.2	*COL8A2*	a/g	0.051 (0.037)	1.05	0.161	-	0.149 (0.071)	0.039	0.016 (0.043)	0.717
**rs4846476**	**1:218526228**	**1q41**	***TGFB2***	**c/g**	**0.023 (0.034)**	1.02	**0.493**	+	**0.103 (0.061)**	**0.115**	**-0.009 (0.040)**	**0.830**
**rs115781177**	**2:33348494**	**2p22-p21**	***LTBP1***	**a/g**	**0.010 (0.057)**	1.01	**0.864**	+	**-0.026 (0.104)**	**0.808**	**0.025 (0.068)**	**0.711**
rs121908120	2:219755011	2q35	*WNT10A*	a/t	0.111 (0.205)	1.12	0.589	+	-0.111 (0.205)	0.601	NA	NA
rs4608502	2:228134155	2q36-q37	*COL4A3*	t/c	-0.013 (0.030)	0.99	0.658	-	0.024 (0.056)	0.683	-0.028 (0.036)	0.429
**3:136138073**	**3:136138073**	**3q22.3**	***STAG1***	**d/r**	**0.061 (0.034)**	1.06	**0.071**	+	**0.064 (0.065)**	**0.346**	**0.060 (0.039)**	**0.127**
rs9822953	3:156472071	3q25.31	*TIPARP*[a]	t/c	-0.053 (0.031)	0.95	0.089	+	-0.024 (0.057)	0.682	-0.065 (0.037)	0.079
rs6445046	3:171933252	3q26.31	*FNDC3B*	t/g	0.110 (0.037)	1.12	0.003	-	0.093 (0.070)	0.192	0.117 (0.044)	0.008
3:177306757	3:177306757	3q26.32	*TBL1XR1*[b]	d/r	-0.032 (0.030)	0.97	0.285	-	-0.049 (0.055)	0.390	-0.025 (0.035)	0.482
rs17024437*	4:149081808	4q31.1	*NR3C2*	a/g	-0.066 (0.054)	0.94	0.221	-	-0.064 (0.102)	0.541	-0.066 (0.063)	0.294
**rs1309531**	**5:64306311**	**5q12.3**	***CWC27***	**a/t**	**0.016 (0.029)**	1.02	**0.577**	+	**0.032 (0.054)**	**0.570**	**0.010 (0.034)**	**0.771**
**rs10064391**	**5:64686659**	**5q12.3**	***ADAMTS6***	**a/g**	**0.028 (0.030)**	1.03	**0.353**	+	**0.050 (0.056)**	**0.376**	**0.019 (0.036)**	**0.603**
**rs249767**	**5:141918585**	**5q31.3**	***FGF1***	**t/c**	**-0.067 (0.035)**	0.94	**0.059**	+	**-0.085 (0.066)**	**0.206**	**-0.060 (0.042)**	**0.160**
**rs35028368**	**5:178671146**	**5q35.3**	***ADAMTS2***	**i/r**	**-0.016 (0.039)**	0.98	**0.678**	**-**	**NA**	**NA**	**-0.016 (0.039)**	**0.679**

					Meta-analysis of POAG cohorts				ANZRAG cohort		NEIGHBORHOOD Consortium	
MarkerID	Chr:BP_hg19	CytoLocation	NearestGene	EA/OA	β (s.e.)	OR†	P-value	Expected effect direction‡	β (s.e.)	P-value	β (s.e)	P-value
**rs13191376**	**6:45522139**	**6p21.1**	***RUNX2***	**t/c**	**-0.050 (0.030)**	0.95	**0.098**	**-**	**-0.051 (0.055)**	**0.374**	**-0.050 (0.036)**	**0.169**
**rs1412710**	**6:75837203**	**6q13**	***COL12A1***	**t/c**	**0.050 (0.040)**	1.05	**0.213**	+	**0.176 (0.072)**	**0.017**	**-0.007 (0.048)**	**0.886**
rs1931656	6:82610188	6q14.1	*FAM46A*	a/t	0.029 (0.029)	1.03	0.307	-	0.036 (0.054)	0.525	0.027 (0.034)	0.425
rs9361886	6:82778502	6q14.1	*FAM46A*	t/c	-0.016 (0.030)	0.98	0.610	+	0.035 (0.053)	0.517	-0.041 (0.037)	0.272
**6:169553553**	**6:169553553**	**6q27**	***THBS2***	**i/r**	**0.064 (0.058)**	1.07	**0.274**	+	**NA**	**NA**	**0.064 (0.058)**	**0.274**
7:66262284	7:66262284	7q11.21	*RABGEF1*[c]	d/r	-0.054 (0.032)	0.95	0.099	-	-0.085 (0.061)	0.171	-0.041 (0.038)	0.288
**rs2106166**	**7:92668332**	**7q21.2**	***CDK6-SAMD9***	**a/t**	**0.014 (0.029)**	1.01	**0.626**	**-**	**0.108 (0.053)**	**0.048**	**-0.026 (0.035)**	**0.455**
**rs3808520**	**8:23164773**	**8p21.3**	***LOXL2***	**c/g**	**0.027 (0.036)**	1.03	**0.447**	**-**	**-0.103 (0.066)**	**0.130**	**0.081 (0.043)**	**0.057**
**rs10429294**	**8:95969322**	**8q22.1**	***NDUFAF6***	**t/c**	**0.020 (0.029)**	1.02	**0.485**	**-**	**0.0336 (0.054)**	**0.543**	**0.015 (0.035)**	**0.668**
**rs7026684**	**9:4215308**	**9p24.2**	***GLIS3***	**a/g**	**0.020 (0.030)**	1.02	**0.496**	+	**0.067 (0.055)**	**0.234**	**0.001 (0.035)**	**0.984**
rs66720556	9:13559717	9p23	*MPDZ-NFIB*	a/t	0.077 (0.037)	1.08	0.034	+	0.104 (0.068)	0.139	0.067 (0.043)	0.124
rs10980623	9:113660537	9q31.3	*LPAR1*	a/g	0.036 (0.035)	1.04	0.303	+	0.124 (0.065)	0.064	0.001 (0.041)	0.986
rs3094339	9:136884738	9q34.2	*VAV2-BRD3*	a/g	0.021 (0.033)	1.02	0.523	-	0.046 (0.062)	0.472	0.011 (0.038)	0.770
rs9409911*	9:137434446	9q34.2	*LOC100506532*	a/g	-0.024 (0.030)	0.98	0.437	-	0.021 (0.057)	0.719	-0.041 (0.036)	0.250
rs1536482	9:137440528	9q34.3	*COL5A1*	a/g	0.026 (0.031)	1.03	0.399	+	0.010 (0.058)	0.862	0.032 (0.037)	0.378
rs56289510*	9:137461867	9q34.3	*COL5A1*	t/c	-0.055 (0.060)	0.95	0.356	+	-0.216 (0.111)	0.058	0.010 (0.070)	0.893

MarkerID	Chr:BP_hg19	CytoLocation	NearestGene	EA/OA	Meta-analysis of POAG cohorts				ANZRAG cohort		NEIGHBORHOOD Consortium	
					β (s.e.)	OR†	P-value	Expected effect direction‡	β (s.e.)	P-value	β (s.e)	P-value
rs7032489	9:137559775	9q34.3	*COL5A1*	c/g	0.009 (0.041)	1.01	0.817	-	-0.018 (0.074)	0.815	0.021 (0.049)	0.662
rs2386136*	9:139864341	9q34.3	*PRR31*	a/g	-0.004 (0.033)	1.00	0.912	+	0.038 (0.055)	0.508	-0.027 (0.041)	0.519
rs35809595	10:63831928	10q21.2	*ARID5B*	a/g	-0.037 (0.030)	0.96	0.212	-	-0.016 (0.055)	0.775	-0.045 (0.035)	0.196
**rs2419835**	**10:115296564**	**10q25.3**	***TCF7L2-HABP2***	**t/c**	**-0.020 (0.041)**	0.98	**0.624**	**+**	**-0.077 (0.081)**	**0.358**	**-2.000E-03 (0.048)**	**0.997**
rs4938174	11:110913240	11q23.1	*ARHGAP20-C11orf53*	a/g	-0.037 (0.031)	0.96	0.237	+	-0.076 (0.058)	0.207	-0.021 (0.037)	0.562
**rs56009602**	**11:130289612**	**11q24.3**	***ADAMTS8***	**t/c**	**0.216 (0.076)**	1.24	**0.005**	**-**	**0.458 (0.137)**	**0.001**	**0.107 (0.092)**	**0.240**
**rs7308752**	**12:91527181**	**12q21.33**	***DCN***	**a/g**	**0.006 (0.049)**	1.01	**0.910**	**-**	**0.138 (0.096)**	**0.159**	**-0.042 (0.057)**	**0.465**
rs116878472	12:104210992	12q23.3	*NT5DC3*	t/c	-0.014 (0.168)	0.99	0.932	-	-0.014 (0.168)	0.934	NA	NA
rs11553764	12:104415244	12q23.3	*GLT8D2*	t/c	-0.028 (0.040)	0.97	0.484	+	-0.141 (0.075)	0.068	0.015 (0.046)	0.739
rs10161679	13:23243645	13q12.11	*FGF9-SGCG*[d]	a/g	0.074 (0.033)	1.08	0.027	+	0.109 (0.063)	0.090	0.060 (0.040)	0.128
rs2755238*	13:41110270	13q14.11	*LOC105370172-FOXO1*	t/c	-0.136 (0.047)	0.87	0.004	+	-0.255 (0.0916)	0.007	-0.094 (0.054)	0.085
**rs56223983**	**14:81814754**	**14q31.1**	***STON2***	**t/g**	**0.001 (0.031)**	1.00	**0.971**	**-**	**0.004 (0.057)**	**0.947**	**0 (0.037)**	**0.999**
rs62014489*	15:30171879	15q13.1	*TJP1*	a/g	0.041 (0.048)	1.04	0.391	+	0.029 (0.090)	0.757	0.046 (0.056)	0.416
**rs8030753**	**15:48801935**	**15q21.1**	***FBN1***	**t/c**	**-0.040 (0.042)**	0.96	**0.341**	**+**	**-0.009 (0.078)**	**0.911**	**-0.052 (0.049)**	**0.293**
rs12912010	15:67467143	15q22.33	*SMAD3*	t/g	-0.001 (0.035)	1.00	0.984	+	-0.002 (0.065)	0.980	-3E-03 (0.041)	0.993
rs4843040	15:85838636	15q25.3	*PDE8A-AKAP13*[e]	t/c	0.039 (0.034)	1.04	0.261	+	-0.008 (0.064)	0.904	0.057 (0.040)	0.160

					Meta-analysis of POAG cohorts				ANZRAG cohort		NEIGHBORHOOD Consortium	
**MarkerID**	**Chr:BP_hg19**	**CytoLocation**	**NearestGene**	**EA/OA**	**β (s.e.)**	**OR†**	**P-value**	**Expected effect direction‡**	**β (s.e.)**	**P-value**	**β (s.e)**	**P-value**
rs2034809	15:101555399	15q26.3	*LRRK1*	a/g	0.007 (0.030)	1.01	0.808	+	0.059 (0.061)	0.352	-0.009 (0.035)	0.794
rs930847	15:101558562	15q26.3	*LRRK1*	t/g	-0.018 (0.035)	0.98	0.608	-	-0.121 (0.063)	0.062	0.027 (0.041)	0.510
rs752092	15:101781934	15q26.3	*CHSY1*	a/g	0.054 (0.031)	1.06	0.076	+	0.103 (0.057)	0.078	0.035 (0.036)	0.344
rs35193497	16:88324821	16q24.2	*ZNF469*	t/g	-0.021 (0.033)	0.98	0.525	-	-0.018 (0.061)	0.771	-0.022 (0.039)	0.573
16:88338872*	16:88338872	16q24.2	*ZNF469-BANP*	i/r	0.048 (0.099)	1.05	0.630	+	NA	NA	0.048 (0.099)	0.631
rs4792535	17:14565130	17p12	*HS3ST3B1-PMP22*	t/c	-0.021 (0.031)	0.98	0.499	-	-0.022 (0.058)	0.714	-0.021 (0.037)	0.574
**rs8133436**	**21:47519535**	**21q22.3**	***COL6A2***	**t/c**	**-0.196 (0.126)**	0.82	**0.121**	+	**-0.248 (0.157)**	**0.119**	**-0.099 (0.212)**	**0.639**
**rs71313932**	**22:19960198**	**22q11.21**	***ARVCF***	**c/g**	**0.0133 (0.032)**	1.01	**0.677**	**-**	**-0.048 (0.059)**	**0.434**	**0.039 (0.038)**	**0.309**

* In LD r2>0.8 with Lead SNP

† OR calculated using the following formula OR = exp(β)

[a] The lead SNP is located in a validated non-coding mRNA, *LINC00886*; [b] The lead SNP is located in a validated non-coding mRNA, *LINC00578*; [c] In Lu et al this locus was reported as two loci (*VKORC1L1* and *C7orf42*); [d] The lead SNP is located 228KB 3' of the pseudogene *BASP1P1*; [e] The lead SNP is located in the pseudogene *ADAMTS7P4*

Independent SNPs found in CoJo analysis are underline

‡ variants in which the CCT-reducing allele also increases the risk of POAG were labeled with positive sign (+), variants in which this condition was not fulfilled were labeled with negative sign (-)

Nearest gene (reference NCBI build37) is given as locus label, but this should not be interpreted as providing support that the nearest gene is the best candidate, a list including all the genes +/- 200kb of the lead SNP is presented in Supplementary Table 12.

Novel CCT-loci are shown in bold

Supplementary Table 6. Tissue enrichment results from DEPICT

MeSH_Term	Name	MeSH first level	MeSH Second level	*P*-value	FDR
**A10.690.467**	**Muscle Smooth**	**Tissues**	**Muscles**	**3.35E-06**	**<0.01**
**A11.329.629**	**Osteoblasts**	**Cells**	**Connective Tissue Cells**	**8.37E-06**	**<0.01**
**A11.620**	**Muscle Cells**	**Cells**	**Muscle Cells**	**2.02E-05**	**<0.01**
**A11.620.520**	**Myocytes Smooth Muscle**	**Cells**	**Muscle Cells**	**2.02E-05**	**<0.01**
**A11.872.580**	**Mesenchymal Stem Cells**	**Cells**	**Stem Cells**	**4.54E-05**	**<0.01**
**A11.329.171**	**Chondrocytes**	**Cells**	**Connective Tissue Cells**	**6.26E-05**	**<0.01**
A07.231.114	Arteries	Cardiovascular System	Blood Vessels	7.30E-05	<0.01
**A10.165.450.300.425**	**Keloid**	**Tissues**	**Connective Tissue**	**1.96E-04**	**<0.01**
**A11.329.830**	**Stromal Cells**	**Cells**	**Connective Tissue Cells**	**3.41E-04**	**<0.01**
**A10.165.450**	**Granulation Tissue**	**Tissues**	**Connective Tissue**	**3.55E-04**	**<0.01**
**A10.165.450.300**	**Cicatrix**	**Tissues**	**Connective Tissue**	**3.55E-04**	**<0.01**
A05.360.319.679.690	Myometrium	Urogenital System	Genitalia	6.65E-04	<0.05
**A11.329.228**	**Fibroblasts**	**Cells**	**Connective Tissue Cells**	**7.33E-04**	**<0.05**
A02.835.583	Joints	Musculoskeletal System	Skeleton	8.08E-04	<0.05
A02.835.583.443	Joint Capsule	Musculoskeletal System	Skeleton	8.08E-04	<0.05
A02.835.583.443.800	Synovial Membrane	Musculoskeletal System	Skeleton	8.08E-04	<0.05
**A11.872.190.260**	**Embryoid Bodies**	**Cells**	**Stem Cells**	**9.47E-04**	**<0.05**
A07.541.510.110	Aortic Valve	Cardiovascular System	Heart	1.05E-03	<0.05
A07.541.510	Heart Valves	Cardiovascular System	Heart	1.05E-03	<0.05
A05.360.319.679	Uterus	Urogenital System	Genitalia	1.22E-03	<0.05
**A10.615.789**	**Serous Membrane**	**Tissues**	**Membranes**	**1.38E-03**	**<0.05**
A05.360.319	Genitalia Female	Urogenital System	Genitalia	2.38E-03	<0.05
**A02.165**	**Cartilage**	**Musculoskeletal System**	**Cartilage**	**2.55E-03**	**<0.05**
**A11.329.114**	**Adipocytes**	**Cells**	**Connective Tissue Cells**	**2.70E-03**	**<0.05**
A05.360.444.492	Penis	Urogenital System	Genitalia	2.95E-03	<0.05
A15.145.846	Serum	Hemic and Immune Systems	Blood	3.66E-03	<0.05

MeSH_Term	Name	MeSH first level	MeSH Second level	*P*-value	FDR
A05.360	Genitalia	Urogenital System	Genitalia	4.07E-03	<0.05
A05.360.444.492.362	Foreskin	Urogenital System	Genitalia	4.39E-03	<0.05
A07.231	Blood Vessels	Cardiovascular System	Blood Vessels	5.20E-03	<0.05
A03.556.875	Upper Gastrointestinal Tract	Digestive System	Gastrointestinal Tract	5.36E-03	<0.05
A05.360.319.679.490	Endometrium	Urogenital System	Genitalia	6.09E-03	<0.05
A03.556.875.500	Esophagus	Digestive System	Gastrointestinal Tract	6.10E-03	<0.05
A05.360.319.679.256	Cervix Uteri	Urogenital System	Genitalia	8.94E-03	<0.05

Results using genome-wide significant (P-value < 5.0e-08) SNPs in DEPICT

Supplementary Table 7. Gene expression of genes prioritized by DEPICT in human cornea, according to the Ocular Tissue Database

**Locus**	**Nr of genes In locus**	**Ensembl_gene_ID**	**Gene_symbol**	**Nominal P-value**	**Gene Closest to lead SNP**	**Cornea**
rs6445046	1	ENSG00000075420	FNDC3B	1.56E-09	TRUE	79.98
rs7026684	1	ENSG00000107249	GLIS3	2.45E-09	FALSE	135.81
**rs1412710**	**1**	**ENSG00000111799**	**COL12A1**	**3.78E-09**	**TRUE**	**721.26**
rs34557764; rs56028712	10	ENSG00000169902	TPST1	4.88E-09	FALSE	20.06
rs201460780	1	ENSG00000186340	THBS2	6.10E-09	TRUE	36.74
rs8133436	2	ENSG00000142173	COL6A2	6.87E-09	TRUE	42.66
rs115781177	1	ENSG00000049323	LTBP1	1.11E-08	TRUE	93.30
rs930847; rs753930	1	ENSG00000154237	LRRK1	1.52E-08	TRUE	56.31
rs8030753	1	ENSG00000166147	FBN1	1.66E-08	TRUE	23.99
rs3132303; rs3132307; rs57953080; rs7851613; rs4304399	1	ENSG00000130635	COL5A1	5.99E-08	TRUE	38.13
**rs12912010**	**1**	**ENSG00000166949**	**SMAD3**	**9.59E-08**	**FALSE**	**222.9**
**rs7308752**	**6**	**ENSG00000011465**	**DCN**	**3.71E-07**	**FALSE**	**1508.98**
rs28498458	1	ENSG00000131873	CHSY1	6.11E-07	TRUE	167.29
rs3808520	2	ENSG00000134013	LOXL2	1.34E-06	FALSE	31.87
5:178671146	1	ENSG00000087116	ADAMTS2	1.39E-06	TRUE	20.65

Locus	Nr of genes In locus	Ensembl_gene_ID	Gene_symbol	Nominal P-value	Gene Closest to lead SNP	Cornea
**rs7308752**	**6**	**ENSG00000139329**	**LUM**	**2.23E-06**	**TRUE**	**538.12**
rs13191376	1	ENSG00000124813	RUNX2	2.49E-06	FALSE	20.40
rs4846476	1	ENSG00000092969	TGFB2	4.46E-06	TRUE	26.63
rs274763	5	ENSG00000171812	COL8A2	6.86E-06	TRUE	81.40
rs35193497; rs6603051 ; rs13335142 ;rs12933008	1	ENSG00000225614	ZNF469	7.28E-06	TRUE	138.92
rs274763	5	ENSG00000126070	EIF2C3	1.02E-05	FALSE	70.82
rs10161679	1	ENSG00000102683	SGCG	1.16E-05	TRUE	NA
rs121908120	5	ENSG00000135925	WNT10A	1.66E-05	TRUE	53.00
rs4718248	4	ENSG00000164669	INTS4L1	2.26E-05	FALSE	25.25
rs7308752	6	ENSG00000083782	EPYC	2.93E-05	FALSE	24.29
rs76242003	1	ENSG00000166106	ADAMTS15	4.04E-05	TRUE	20.40
rs66720556	1	ENSG00000107186	MPDZ	5.02E-05	TRUE	26.63
rs785422	1	ENSG00000104067	TJP1	5.43E-05	TRUE	181.82
rs2755238	1	ENSG00000150907	FOXO1	6.20E-05	TRUE	138.92
rs11553764; rs116878472	10	ENSG00000120820	GLT8D2	6.89E-05	TRUE	128.54
rs2654583	1	ENSG00000154227	CERS3	1.04E-04	TRUE	NA
rs4843040; rs11855750	2	ENSG00000170776	AKAP13	1.25E-04	FALSE	130.09
rs4608502	3	ENSG00000236432	-	1.54E-04	FALSE	NA

Locus	Nr of genes In locus	Ensembl_gene_ID	Gene_symbol	Nominal P-value	Gene Closest to lead SNP	Cornea
**rs7308752**	**6**	**ENSG00000139330**	**KERA**	**2.24E-04**	**FALSE**	**6590.28**
rs4792535	1	ENSG00000232058	-	2.94E-04	TRUE	
rs274763	5	ENSG00000092850	TEKT2	4.96E-04	FALSE	29.30
rs8133436	2	ENSG00000160282	FTCD	5.27E-04	FALSE	48.45
rs10471310; rs201319128; rs7723472	1	ENSG00000049192	ADAMTS6	9.22E-04	TRUE	18.58
rs56009602; rs73044822	2	ENSG00000134917	ADAMTS8	1.37E-03	TRUE	27.88
rs2419835	1	ENSG00000148702	HABP2	1.41E-03	TRUE	27.03
rs35809595	1	ENSG00000150347	ARID5B	1.90E-03	TRUE	NA
rs10980623; rs56761894	1	ENSG00000198121	LPAR1	2.16E-03	TRUE	88.32
rs34557764; rs56028712	10	ENSG00000106609	C7orf42	2.25E-03	TRUE	66.68
rs410876	3	ENSG00000235138	-	3.67E-03	FALSE	NA
rs71313931	1	ENSG00000099889	ARVCF	4.04E-03	FALSE	32.85
rs4718248	4	ENSG00000152926	ZNF117	4.58E-03	FALSE	25.88
rs201512053	8	ENSG00000073711	PPP2R3A	6.66E-03	FALSE	26.90
rs4608502	3	ENSG00000169031	COL4A3	0.01	FALSE	82.37
rs56223983	1	ENSG00000140022	STON2	0.02	TRUE	35.62
rs4843040; rs11855750	2	ENSG00000229212	-	0.03	TRUE	NA
rs9822953; rs182936	3	ENSG00000163659	TIPARP	0.03	FALSE	134.31

Locus	Nr of genes In locus	Ensembl_gene_ID	Gene_symbol	Nominal P-value	Gene Closest to lead SNP	Cornea
rs1931656	1	ENSG00000224995	-	0.04	TRUE	NA
rs56009602; rs73044822	2	ENSG00000175773	-	0.05	FALSE	NA
rs410876	3	ENSG00000169925	BRD3	0.05	FALSE	44.56

In the Ocular Tissue Database (OTDB), the gene expression is indicated as Affymetrix Probe Logarithmic Intensity Error (PLIER) normalized value. The PLIER normalization method was described by Wagner et al. The OTDB is available at https://genome.uiowa.edu/otdb/. In red the top five most expressed genes

Supplementary Table 8. Top 30 Biosystems gene-sets or pathways observed through pathway analysis using INRICH approach

Pathway or gene-set	#Genes	#INTERVAL EURO	p_EURO	#INTERVAL Asn	p_ASN	Combined p FishersMethod
487440 GO:0001501 skeletal system development	460	31	3.999E-04	8	7.998E-04	5.104E-06
516560 GO:0071636 positive regulation of transforming growth factor beta production	14	4	1.800E-03	2	3.999E-04	1.090E-05
516558 GO:0071634 regulation of transforming growth factor beta production	23	5	2.200E-03	2	1.600E-03	4.770E-05
499609 GO:0030199 collagen fibril organization	37	11	3.999E-04	3	1.960E-02	9.997E-05
487718 GO:0001822 kidney development	256	23	2.599E-03	6	3.199E-03	1.056E-04
752191 GO:0061448 connective tissue development	201	19	2.000E-03	5	4.999E-03	1.251E-04
500302 GO:0031012 extracellular matrix	410	30	5.999E-04	6	2.360E-02	1.722E-04
499608 GO:0030198 extracellular matrix organization	348	28	5.999E-04	5	3.999E-02	2.792E-04
516920 GO:0072001 renal system development	274	23	8.398E-03	6	3.399E-03	3.273E-04
1270244 R-HSA-1474244 Extracellular matrix organization	256	23	1.400E-03	5	2.180E-02	3.477E-04
490453 GO:0005581 collagen trimer	84	10	5.099E-02	5	9.998E-04	5.549E-04
490653 GO:0005578 proteinaceous extracellular matrix	343	28	9.998E-04	5	5.379E-02	5.825E-04
494250 GO:0010171 body morphogenesis	47	7	8.998E-03	2	6.999E-03	6.721E-04
506148 GO:0043062 extracellular structure organization	351	29	1.400E-03	5	4.839E-02	7.180E-04
507369 GO:0044420 extracellular matrix component	132	16	1.440E-02	5	5.999E-03	8.945E-04

513879 GO:0060323 head morphogenesis	38	5	2.679E-02	2	3.399E-03	9.385E-04
1270260 R-HSA-216083 Integrin cell surface interactions	66	8	4.799E-03	2	2.040E-02	1.001E-03
487568 GO:0001655 urogenital system development	312	23	1.860E-02	6	5.999E-03	1.127E-03
490452 GO:0005201 extracellular matrix structural constituent	64	7	2.224E-01	5	5.999E-04	1.323E-03
513881 GO:0060325 face morphogenesis	33	4	4.959E-02	2	2.799E-03	1.372E-03
492160 GO:0009887 organ morphogenesis	890	39	1.178E-01	10	1.200E-03	1.394E-03
492160 GO:0009887 organ morphogenesis	890	39	1.186E-01	10	1.200E-03	1.402E-03
499352 GO:0022617 extracellular matrix disassembly	117	10	2.438E-01	5	7.998E-04	1.860E-03
510765 GO:0048710 regulation of astrocyte differentiation	28	7	2.000E-04	0	1.000E+00	1.903E-03
510767 GO:0048712 negative regulation of astrocyte differentiation	14	7	2.000E-04	0	1.000E+00	1.903E-03
513880 GO:0060324 face development	52	6	2.080E-02	2	1.040E-02	2.041E-03
493898 GO:0009791 post-embryonic development	109	8	2.252E-01	4	1.400E-03	2.856E-03
517274 GO:0072358 cardiovascular system development	820	44	9.998E-03	8	3.339E-02	3.006E-03
507243 GO:0044236 multicellular organism metabolic process	100	12	3.679E-02	4	9.398E-03	3.102E-03
512374 GO:0051496 positive regulation of stress fiber assembly	42	5	4.059E-02	2	8.598E-03	3.127E-03

Supplementary Table 9. Top 30 diseases and bio functions observed using Ingenuity Pathway Analysis (IPA) software

Category	B-H *P*-value	Molecules
Cancer	4.6E-09-5.08E-02	*RRP15,GLT8D2,ADAMTS8,ABCA2,ZNF469,C9orf139,FEV,FNDC3B,EPYC,FUT7,NT5DC3,ADAMTS2,THRAP3,LUM,CDK5R2,TNFRSF10C,LSS,SPATC1L,HEPACAM2,SBDS,PLEKHF2,TP53INP1,CCNE2,ARHGAP10,STK36,KERA,DCN,TRAPPC3,PDE8A,FTCD,AKAP13,RTL10,SAMD9L,ADAMTS6,FBXW5,ZNF354C,THBS2,NR3C2,RXRA,TEKT2,ENTPD4,STON2,NPDC1,COL4A3,TTLL4,RTKN2,FILIP1L,TYW1,COL5A1,RABL6,TIPARP,ALDH1A3,CFAP65,MUSK,DGCR8,CLIC3,GNB1L,PAXX,DUT,TJP1,COL12A1,NRAP,ARVCF,RABGEF1,LTBP1,TRAF2,UAP1L1,HCFC2,COX7A2,CHMP7,IHH,SUPT3H,COL4A4,PCBP3,CASP7,C8G,INTS8,ZDHHC8,SMAD3,DPP7,ZFPM1,PCCB,WNT6,MRPS31,TMEM30A,LCN12,LEKR1,SSR3,DPY19L4,TDG,STAG1,RUNX2,COMT,IQCH,AAGAB,PHPT1,AGO1,ADPRHL2,TNFRSF10D,PTGDS,TXNRD1,ZBTB44,SEL1L,ARHGAP21,NDUFAF6,FBN1,CCDC183,TMEM248,TM4SF20,TXNRD2,COL8A2,SAPCD2,GLIS3,MCM3AP,CHSY1,CRYBA2,MAN1B1,MFF,GTF2A1,LRRK1,LOXL2,FILIP1,HSP90B1,COL6A1,KCTD7,TGFB2,TNFRSF10A,NFYB,LCNL1,ADAMTS15,GRIN1,NHEJ1,COL6A2,CYP27A1,MAMDC4,PRKAG3,EDF1,FGF1,MAP7D1,FOXO1,WNT10A,HABP2,LPAR1,ENTPD2,FAM46A,TANGO2,TRMT2A,SAMD9*
Gastrointestinal Disease	4.6E-09-5.08E-02	*RRP15,GLT8D2,ADAMTS8,ABCA2,ZNF469,C9orf139,EPYC,FNDC3B,FUT7,ADAMTS2,THRAP3,CDK5R2,TNFRSF10C,LSS,HEPACAM2,SPATC1L,PLEKHF2,CCNE2,TP53INP1,ARHGAP10,STK36,KERA,DCN,TRAPPC3,FTCD,PDE8A,AKAP13,SAMD9L,FBXW5,ADAMTS6,ZNF354C,THBS2,NR3C2,RXRA,TEKT2,ENTPD4,NPDC1,STON2,COL4A3,TTLL4,FILIP1L,RTKN2,TYW1,COL5A1,RABL6,TIPARP,ALDH1A3,MUSK,DGCR8,GNB1L,PAXX,TJP1,COL12A1,ARVCF,NRAP,RABGEF1,LTBP1,TRAF2,UAP1L1,HCFC2,COX7A2,CHMP7,IHH,SUPT3H,COL4A4,C8G,CASP7,INTS8,ZDHHC8,SMAD3,DPP7,ZFPM1,WNT6,PCCB,MRPS31,LEKR1,LCN12,TDG,DPY19L4,STAG1,RUNX2,COMT,IQCH,AAGAB,PHPT1,ADPRHL2,TNFRSF10D,PTGDS,TXNRD1,ZBTB44,SEL1L,ARHGAP21,NDUFAF6,FBN1,CCDC183,TMEM248,TXNRD2,TM4SF20,COL8A2,GLIS3,SAPCD2,CHSY1,MCM3AP,MAN1B1,CRYBA2,GTF2A1,LRRK1,LOXL2,FILIP1,HSP90B1,COL6A1,TGFB2,TNFRSF10A,NFYB,LCNL1,ADAMTS15,NHEJ1,GRIN1,CYP27A1,COL6A2,MAMDC4,PRKAG3,EDF1,FGF1,MAP7D1,FOXO1,LPAR1,WNT10A,HABP2,ENTPD2,TANGO2,FAM46A,TRMT2A,SAMD9*
Organismal Injury and Abnormalities	4.6E-09-5.08E-02	*RRP15,GLT8D2,ADAMTS8,ABCA2,ZNF469,C9orf139,FEV,FNDC3B,EPYC,FUT7,NT5DC3,ADAMTS2,THRAP3,LUM,CDK5R2,LSS,TNFRSF10C,HEPACAM2,SPATC1L,SBDS,PLEKHF2,TP53INP1,CCNE2,STK36,ARHGAP10,KERA,DCN,TRAPPC3,FTCD,PDE8A,AKAP13,RTL10,SAMD9L,FBXW5,ADAMTS6,ZNF354C,THBS2,NR3C2,RXRA,TEKT2,ENTPD4,STON2,NPDC1,COL4A3,TTLL4,FILIP1L,RTKN2,TYW1,COL5A1,RABL6,TIPARP,ALDH1A3,CFAP65,MUSK,DGCR8,CLIC3,GNB1L,PAXX,DUT,TJP1,COL12A1,ARVCF,NRAP,RABGEF1,LT*

Category	B-H *P*-value	Molecules
		BP1,TRAF2,UAP1L1,HCFC2,COX7A2,CHMP7,IHH,COL4A4,SUPT3H,PCBP3,C8G,CASP7,INTS8,ZDHHC8,SMAD3,DPP7,ZFPM1,PCCB,WNT6,MRPS31,TMEM30A,LCN12,LEKR1,SSR3,TDG,DPY19L4,RUNX2,STAG1,COMT,IQCH,PHPT1,AAGAB,AGO1,ADPRHL2,TNFRSF10D,PTGDS,TXNRD1,ZBTB44,SEL1L,ARHGAP21,FBN1,NDUFAF6,CCDC183,TMEM248,TXNRD2,TM4SF20,COL8A2,GLIS3,SAPCD2,MCM3AP,CHSY1,MAN1B1,CRYBA2,MFF,GTF2A1,LRRK1,FILIP1,LOXL2,HSP90B1,KCTD7,COL6A1,TGFB2,TNFRSF10A,NFYB,LCNL1,ADAMTS15,NHEJ1,GRIN1,CYP27A1,COL6A2,MAMDC4,PRKAG3,EDF1,FGF1,MAP7D1,HABP2,LPAR1,FOXO1,WNT10A,ENTPD2,FAM46A,TANGO2,TRMT2A,SAMD9
**Connective Tissue Disorders**	**7.83E-08-5.08E-02**	***COL8A2,ADAMTS8,ZNF469,SMAD3,COL4A3,CHSY1,MAN1B1,LRRK1,ADAMTS2,COL5A1,HSP90B1,COL6A1,ALDH1A3,RUNX2,LUM,TGFB2,TNFRSF10C,TNFRSF10A,ADAMTS15,STK36,COL6A2,TJP1,DCN,COL12A1,TNFRSF10D,PTGDS,PDE8A,SEL1L,AKAP13,SAMD9L,ADAMTS6,WNT10A,FOXO1,THBS2,FBN1,IHH,COL4A4,RXRA,SAMD9***
Reproductive System Disease	3.19E-06-5.08E-02	*GLT8D2,ZDHHC8,ZFPM1,WNT6,PCCB,EPYC,MRPS31,FNDC3B,FUT7,ADAMTS2,DPY19L4,THRAP3,RUNX2,STAG1,COMT,LUM,LSS,TNFRSF10C,ADPRHL2,HEPACAM2,TP53INP1,KERA,DCN,PTGDS,TXNRD1,ZBTB44,FTCD,SEL1L,AKAP13,SAMD9L,ARHGAP21,ZNF354C,THBS2,FBN1,NR3C2,RXRA,TM4SF20,TEKT2,COL8A2,NPDC1,SAPCD2,COL4A3,MCM3AP,TTLL4,RTKN2,FILIP1L,GTF2A1,LRRK1,FILIP1,LOXL2,TYW1,COL5A1,RABL6,HSP90B1,COL6A1,TIPARP,ALDH1A3,CFAP65,DGCR8,MUSK,TGFB2,TNFRSF10A,ADAMTS15,NHEJ1,PAXX,GRIN1,DUT,COL6A2,COL12A1,PRKAG3,NRAP,ARVCF,FGF1,LTBP1,TRAF2,MAP7D1,FOXO1,LPAR1,ENTPD2,COX7A2,IHH,COL4A4,SUPT3H,SAMD9,CASP7,INTS8*
Endocrine System Disorders	9.4E-06-5.08E-02	*ADAMTS8,ZDHHC8,ABCA2,SMAD3,DPP7,WNT6,PCCB,FNDC3B,FUT7,LCN12,ADAMTS2,TDG,THRAP3,STAG1,RUNX2,CDK5R2,TNFRSF10C,LSS,IQCH,PLEKHF2,CCNE2,TP53INP1,STK36,DCN,PTGDS,TRAPPC3,AKAP13,ADAMTS6,THBS2,NR3C2,CCDC183,RXRA,TEKT2,COL8A2,GLIS3,COL4A3,MCM3AP,MAN1B1,FILIP1,LOXL2,COL5A1,HSP90B1,RABL6,COL6A1,MUSK,DGCR8,TGFB2,TNFRSF10A,ADAMTS15,GRIN1,PAXX,DUT,COL6A2,TJP1,COL12A1,MAMDC4,NRAP,FGF1,LTBP1,TRAF2,MAP7D1,FOXO1,HABP2,ENTPD2,UAP1L1,FAM46A,COL4A4,SAMD9,C8G*
Metabolic Disease	9.4E-06-5.08E-02	*GRIN1,COL8A2,COL6A2,ZNF469,COL12A1,COL4A3,WNT6,PTGDS,MFF,ADAMTS2,FGF1,COL5A1,FTCD,COL6A1,COMT,MUSK,COL4A4,NR3C2*
**Skeletal and Muscular Disorders**	**9.4E-06-5.08E-02**	***COL8A2,ZNF469,SMAD3,COL4A3,CHSY1,MAN1B1,LRRK1,LOXL2,ADAMTS2,COL5A1,TDG,HSP90B1,COL6A1,ALDH1A3,RUNX2,LUM,MUSK,TGFB2,TNFRSF10C,TNFRSF10A,ADAMTS15,STK36,COL6A2,TJP1,DCN,COL12A1,TNFRSF10D,PTGDS,LTBP1,FGF1,PDE8A,SEL1L,AKAP13,SAMD9L,ADAMTS6,WNT10A,FOXO1,THBS2,FBN1,IHH,COL4A4,RXRA***
Immunological Disease	1.76E-05-5.08E-02	*COL8A2,GLIS3,STON2,SAPCD2,COL4A3,SMAD3,CHSY1,FILIP1L,TMEM30A,FUT7,LEKR1,HSP90B1,COL6A1,THRAP3,RUNX2,COMT,MUSK,LUM,TGFB2,CLIC3,TNFRSF10C,TNFRSF10A,ADAMTS15,NHEJ1,GRIN1,STK36,COL6A2,TJP1,DCN,COL12A1,TNFRSF10D,PTGDS,NRAP,TXNRD1,LTBP1,RABGEF1,ZBTB44,FTCD,PDE8A,AKAP13,TRAF2,LPAR1,FOXO1,ENTPD2,FBN1,COL4A4,RXRA,SAMD9,CASP7*

Category	B-H *P*-value	Molecules
**Tissue Morphology**	**3.44E-05-5.08E-02**	***COL8A2,GLIS3,STON2,COL4A3,SMAD3,CHSY1,FEV,ZFPM1,FUT7,LRRK1,COL5A1,TDG,HSP90B1,ALDH1A3,RUNX2,COMT,LUM,MUSK,DGCR8,CDK5R2,TGFB2,TNFRSF10A,GRIN1,NHEJ1,TP53INP1,CCNE2,ARHGAP10,KERA,CYP27A1,COL12A1,DCN,PTGDS,TXNRD1,LTBP1,RABGEF1,FGF1,AKAP13,TRAF2,FOXO1,LPAR1,THBS2,FBN1,IHH,NR3C2,RXRA***
Dermatological Diseases and Conditions	4.89E-05-5.08E-02	*GLT8D2,ADAMTS8,ABCA2,ZDHHC8,ZNF469,SMAD3,C9orf139,EPYC,NT5DC3,LCN12,LEKR1,ADAMTS2,DPY19L4,TDG,THRAP3,RUNX2,LUM,CDK5R2,LSS,TNFRSF10C,IQCH,HEPACAM2,ADPRHL2,STK36,KERA,DCN,TNFRSF10D,TRAPPC3,FTCD,SEL1L,AKAP13,SAMD9L,ADAMTS6,FBXW5,ARHGAP21,ZNF354C,THBS2,FBN1,NR3C2,RXRA,TXNRD2,TEKT2,COL8A2,ENTPD4,STON2,NPDC1,GLIS3,COL4A3,MAN1B1,FILIP1L,RTKN2,LRRK1,FILIP1,TYW1,LOXL2,COL5A1,HSP90B1,KCTD7,COL6A1,TIPARP,DGCR8,MUSK,TGFB2,TNFRSF10A,LCNL1,ADAMTS15,NHEJ1,GRIN1,CYP27A1,COL6A2,TJP1,COL12A1,MAMDC4,ARVCF,NRAP,RABGEF1,LTBP1,MAP7D1,LPAR1,HABP2,WNT10A,ENTPD2,TANGO2,FAM46A,IHH,COL4A4,TRMT2A,PCBP3,SAMD9*
**Hair and Skin Development and Function**	**4.89E-05-5.08E-02**	***DCN,SMAD3,FUT7,ADAMTS2,TXNRD1,FGF1,COL5A1,FOXO1,RUNX2,LUM,THBS2,TGFB2,FBN1,RXRA***
Cell Morphology	5.07E-05-5.08E-02	*COL8A2,ABCA2,ZDHHC8,GLIS3,STON2,SMAD3,COL4A3,CHSY1,FEV,ZFPM1,LOXL2,ADAMTS2,COL5A1,HSP90B1,RUNX2,LUM,MUSK,CDK5R2,TGFB2,GRIN1,TP53INP1,ARHGAP10,CYP27A1,TJP1,DCN,COL12A1,PTGDS,RABGEF1,FGF1,AKAP13,TRAF2,FOXO1,LPAR1,THBS2,IHH,FBN1,RXRA*
Cellular Assembly and Organization	5.07E-05-5.08E-02	*GRIN1,TJP1,SMAD3,DCN,MFF,LOXL2,ADAMTS2,COL5A1,MUSK,LUM,THBS2,TGFB2,FBN1,RXRA*
Embryonic Development	5.64E-05-5.08E-02	*COL8A2,GLIS3,COL4A3,SMAD3,ZFPM1,WNT6,FILIP1L,FNDC3B,ADAMTS2,LOXL2,COL5A1,TDG,HSP90B1,TIPARP,ALDH1A3,RUNX2,COMT,LUM,CDK5R2,TGFB2,SBDS,GRIN1,CCNE2,STK36,CYP27A1,KERA,DCN,TXNRD1,FGF1,LTBP1,AKAP13,SAMD9L,ADAMTS6,FOXO1,WNT10A,THBS2,FBN1,IHH,COL4A4,NR3C2,RXRA,TXNRD2,CASP7*
Nervous System Development and Function	5.64E-05-5.08E-02	*GRIN1,COL8A2,ZDHHC8,KERA,COL4A3,FGF1,COL5A1,ALDH1A3,COMT,MUSK,THBS2,LUM,CDK5R2,TGFB2,IHH,NR3C2,RXRA*
**Ophthalmic Disease**	**5.64E-05-5.08E-02**	***COL8A2,GRIN1,ZNF469,KERA,TJP1,COL4A3,DCN,CRYBA2,LOXL2,LTBP1,HSP90B1,LPAR1,ALDH1A3,LUM,TGFB2,FBN1,NR3C2,LSS,RXRA***
Organ Development	5.64E-05-5.08E-02	*COL8A2,GLIS3,COL4A3,SMAD3,ZFPM1,WNT6,FILIP1L,LOXL2,COL5A1,TDG,TIPARP,RUNX2,ALDH1A3,COMT,DGCR8,LUM,TGFB2,CDK5R2,TNFRSF10A,GRIN1,STK36,CYP27A1,KERA,PTGDS,LTBP1,FGF1,AKAP13,ADAMTS6,LPAR1,FOXO1,HABP2,THBS2,FBN1,IHH,NR3C2,COL4A4,RXRA,TXNRD2,CASP7*

**Category**	**B-H *P*-value**	**Molecules**
Organ Morphology	5.64E-05-5.08E-02	*COL8A2,GLIS3,SMAD3,COL4A3,CHSY1,ZFPM1,WNT6,LRRK1,LOXL2,ADAMTS2,COL5A1,TDG,HSP90B1,COL6A1,ALDH1A3,RUNX2,COMT,LUM,TGFB2,TNFRSF10A,CYP27A1,KERA,COL12A1,DCN,LTBP1,FGF1,RABGEF1,AKAP13,FOXO1,WNT10A,ENTPD2,THBS2,FBN1,IHH,NR3C2,RXRA,TXNRD2,CASP7*
Organismal Development	5.64E-05-5.08E-02	*ADAMTS8,SMAD3,ZFPM1,WNT6,FNDC3B,FUT7,ADAMTS2,TDG,THRAP3,RUNX2,COMT,LUM,CDK5R2,SBDS,TP53INP1,CCNE2,STK36,KERA,DCN,TXNRD1,AKAP13,SAMD9L,ADAMTS6,THBS2,FBN1,NR3C2,RXRA,TXNRD2,COL8A2,GLIS3,COL4A3,CHSY1,FILIP1L,LRRK1,LOXL2,COL5A1,HSP90B1,TIPARP,ALDH1A3,MUSK,DGCR8,TGFB2,TNFRSF10A,NHEJ1,GRIN1,CYP27A1,TJP1,COL12A1,EDF1,LTBP1,FGF1,TRAF2,WNT10A,HABP2,FOXO1,ENTPD2,IHH,COL4A4,CASP7*
**Tissue Development**	**5.64E-05-5.08E-02**	***ADAMTS8,SMAD3,ZFPM1,WNT6,FNDC3B,ADAMTS2,TDG,THRAP3,RUNX2,STAG1,COMT,LUM,CDK5R2,SBDS,TP53INP1,STK36,KERA,DCN,PTGDS,AKAP13,ADAMTS6,THBS2,FBN1,NR3C2,RXRA,TXNRD2,COL8A2,GLIS3,COL4A3,CHSY1,FILIP1L,LRRK1,LOXL2,COL5A1,HSP90B1,COL6A1,TIPARP,ALDH1A3,MUSK,DGCR8,TGFB2,TNFRSF10A,GRIN1,CYP27A1,COL12A1,EDF1,LTBP1,FGF1,FOXO1,HABP2,LPAR1,WNT10A,IHH,COL4A4,CASP7***
**Visual System Development and Function**	**5.64E-05-5.08E-02**	***COL5A1,COL8A2,KERA,ALDH1A3,COL4A3,THBS2,LUM,TGFB2,IHH,RXRA,FGF1***
Hereditary Disorder	9.11E-05-5.08E-02	*COL8A2,ZNF469,SMAD3,COL4A3,CHSY1,CRYBA2,MAN1B1,FILIP1L,MFF,LRRK1,LOXL2,ADAMTS2,COL5A1,COL6A1,RUNX2,MUSK,DGCR8,TGFB2,LSS,NHEJ1,GRIN1,CCNE2,COL6A2,KERA,DCN,COL12A1,FGF1,LTBP1,FTCD,AKAP13,SAMD9L,WNT10A,HABP2,IHH,FBN1,NR3C2,COL4A4,RXRA,TM4SF20,TXNRD2,SAMD9*
Cardiovascular System Development and Function	9.26E-05-5.08E-02	*COL8A2,ADAMTS8,SMAD3,COL4A3,ZFPM1,FILIP1L,LOXL2,ADAMTS2,COL5A1,TDG,HSP90B1,TIPARP,THRAP3,RUNX2,COMT,LUM,DGCR8,TGFB2,SBDS,CCNE2,TJP1,DCN,EDF1,FGF1,LTBP1,AKAP13,ADAMTS6,HABP2,FOXO1,THBS2,IHH,FBN1,NR3C2,RXRA,TXNRD2,CASP7*
Developmental Disorder	1.36E-04-5.08E-02	*COL8A2,ZNF469,SMAD3,CHSY1,ZFPM1,MAN1B1,CRYBA2,MFF,FUT7,LOXL2,ADAMTS2,COL5A1,TDG,HSP90B1,COL6A1,ALDH1A3,RUNX2,DGCR8,MUSK,CDK5R2,TGFB2,LSS,NHEJ1,GRIN1,CCNE2,COL6A2,KERA,COL12A1,DCN,TXNRD1,LTBP1,FGF1,FTCD,AKAP13,TRAF2,LPAR1,FBN1,IHH,RXRA,TXNRD2,SAMD9*
**Connective Tissue Development and Function**	**3.51E-04-5.08E-02**	***GLIS3,COL4A3,SMAD3,CHSY1,ZFPM1,FNDC3B,TMEM30A,LRRK1,LOXL2,ADAMTS2,COL6A1,THRAP3,STAG1,ALDH1A3,RUNX2,COMT,LUM,TGFB2,TNFRSF10A,TP53INP1,STK36,CYP27A1,COL12A1,DCN,PTGDS,FGF1,WNT10A,LPAR1,FOXO1,THBS2,IHH,FBN1,NR3C2,RXRA,TXNRD2***
**Skeletal and Muscular System**	**3.51E-04-5.08E-02**	***SMAD3,CHSY1,FNDC3B,LRRK1,ADAMTS2,TDG,HSP90B1,COL6A1,TIPARP,RUNX2,ALDH1A3,COMT,DGCR8,LUM,TGFB2,TNFRSF10A,SBDS,STK36,DCN,COL12A1,PTGDS,FGF1,LTBP1,AKAP13,TRAF2,LPAR1,HABP2,FOXO1,WNT10A,THBS2,IHH,FBN1,RXRA***

Category	B-H *P*-value	Molecules
**Development and Function**		
Hematological Disease	5.99E-04-5.08E-02	*SMAD3,FNDC3B,FUT7,TMEM30A,LEKR1,THRAP3,STAG1,RUNX2,COMT,LUM,TNFRSF10C,SBDS,DCN,TNFRSF10D,TXNRD1,ZBTB44,PDE8A,FTCD,AKAP13,SAMD9L,FBN1,RXRA,COL8A2,SAPCD2,STON2,GLIS3,COL4A3,CHSY1,FILIP1L,LOXL2,HSP90B1,COL6A1,MUSK,TGFB2,CLIC3,TNFRSF10A,ADAMTS15,GRIN1,NHEJ1,COL6A2,TJP1,COL12A1,MAMDC4,NRAP,RABGEF1,LTBP1,TRAF2,HABP2,FOXO1,LPAR1,ENTPD2,COL4A4,CASP7,SAMD9*
Cellular Function and Maintenance	8.35E-04-5.08E-02	*GRIN1,ADAMTS8,SMAD3,DCN,COL4A3,PTGDS,FNDC3B,ADAMTS2,EDF1,LOXL2,FGF1,COL5A1,FOXO1,HABP2,THRAP3,RUNX2,MUSK,LUM,THBS2,TGFB2,IHH*
Cardiovascular Disease	8.4E-04-5.08E-02	*COL8A2,COL4A3,SMAD3,FUT7,LOXL2,COL5A1,TDG,HSP90B1,RUNX2,COMT,LUM,DGCR8,TGFB2,GRIN1,CCNE2,CYP27A1,PTGDS,LTBP1,PDE8A,AKAP13,FOXO1,HABP2,THBS2,FBN1,NR3C2,RXRA,TXNRD2,CASP7*

Category, refers to a general functional category, for example "connective tissue disorder", which includes subcategories, for example "Dupuytren contracture" see (Supplementary Table 10). B-H-p-value, refers to the -log Benjamini-Hochberg corrected P-value. The B-H p-value column, shows the range of P-values (the lowest and highest) of the subcategories belonging to each general category. Molecules, refers to the genes involved in the general category. Top 30 Diseases and Bio functions are shown in the table

Supplementary Table 10. Diseases and Bio functions related to "connective tissue" and "ophthalmic disease" categories found using Ingenuity Pathway Analysis (IPA) software

Category	Function	Function Annotation	*P*-value	B-H *P*-value	Molecules	# Molecules
Connective Tissue Disorders	Dupuytren contracture	Dupuytren contracture	1.29E-10	7.83E-08	ADAMTS2, ADAMTS6, ADAMTS8, COL12A1, COL4A3, COL4A4, COL5A1, COL6A1, COL6A2, COL8A2	10
Connective Tissue Disorders	Dupuytren contracture	Advanced Dupuytren contracture	9.78E-09	5.94E-06	COL12A1, COL4A3, COL4A4, COL5A1, COL6A1, COL6A2, COL8A2	7
Connective Tissue Disorders	collagen disease	Collagen disease	9.55E-08	5.80E-05	COL12A1, COL4A3, COL4A4, COL6A1, COL6A2	5
Connective Tissue Disorders	hereditary connective tissue disorder	Hereditary connective tissue disorder	8.17E-07	4.96E-04	ADAMTS2, CHSY1, COL12A1, COL4A3, COL4A4, COL5A1, COL6A1, COL6A2, FBN1, IHH, LRRK1, RUNX2, SAMD9, SMAD3, TGFB2, ZNF469	16
Connective Tissue Disorders	Bethlem myopathy	Bethlem myopathy	1.38E-06	8.38E-04	COL12A1, COL6A1, COL6A2	3
Connective Tissue Disorders	Ullrich congenital muscular dystrophy	Ullrich congenital muscular dystrophy	1.38E-06	8.38E-04	COL12A1, COL6A1, COL6A2	3
Connective Tissue Disorders	abnormal morphology	Abnormal morphology of chondrocytes	4.52E-06	2.74E-03	CHSY1, IHH, RUNX2, SMAD3, TGFB2	5
Connective Tissue Disorders	abnormal morphology	Abnormal morphology of cartilage tissue	6.64E-06	4.03E-03	CHSY1, IHH, LUM, RUNX2, RXRA, SMAD3, TGFB2	7
Connective Tissue Disorders	abnormal morphology	Abnormal morphology of rib	1.91E-05	1.16E-02	CHSY1, FBN1, IHH, RUNX2, SMAD3, TGFB2	6
Connective Tissue Disorders	abnormal morphology	Abnormal morphology of tendon	4.01E-05	2.43E-02	DCN, LUM, THBS2	3

Category	Function	Function Annotation	*P*-value	B-H *P*-value	Molecules	# Molecules
Connective Tissue Disorders	abnormality	Abnormality of cartilage tissue	2.48E-05	1.51E-02	CHSY1, IHH, LUM, PDE8A, RUNX2, RXRA, SMAD3, TGFB2, THBS2	9
Connective Tissue Disorders	Alport syndrome	Autosomal dominant Alport syndrome	4.97E-05	3.02E-02	COL4A3, COL4A4	2
Connective Tissue Disorders	Alport syndrome	Autosomal recessive Alport syndrome	4.97E-05	3.02E-02	COL4A3, COL4A4	2
Connective Tissue Disorders	lack	Lack of occipital bone	4.97E-05	3.02E-02	RUNX2, TGFB2	2
Connective Tissue Disorders	thin basement membrane disease	Thin basement membrane disease	4.97E-05	3.02E-02	COL4A3, COL4A4	2
Ophthalmic Disease	thinning	Thinning of cornea	4.88E-07	2.96E-04	COL8A2, KERA, LUM, TGFB2	4
Ophthalmic Disease	abnormal morphology	Abnormal morphology of corneal stroma	6.80E-06	4.13E-03	KERA, LUM, RXRA, TGFB2	4
Ophthalmic Disease	abnormal morphology	Abnormal morphology of anterior segment of the eye	1.59E-05	9.65E-03	ALDH1A3, COL4A3, COL8A2, KERA, LUM, RXRA, TGFB2	7
Ophthalmic Disease	abnormal morphology	Abnormal morphology of cornea	5.60E-05	3.40E-02	COL8A2, KERA, LUM, RXRA, TGFB2	5
Connective Tissue Development and Function	abnormal morphology	Abnormal morphology of chondrocytes	4.52E-06	2.74E-03	CHSY1, IHH, RUNX2, SMAD3, TGFB2	5
Connective Tissue Development and Function	abnormal morphology	Abnormal morphology of cartilage tissue	6.64E-06	4.03E-03	CHSY1, IHH, LUM, RUNX2, RXRA, SMAD3, TGFB2	7
Connective Tissue Development and Function	abnormal morphology	Abnormal morphology of rib	1.91E-05	1.16E-02	CHSY1, FBN1, IHH, RUNX2, SMAD3, TGFB2	6
Connective Tissue Development and Function	morphology	Morphology of bone	4.39E-05	2.66E-02	ADAMTS2, ALDH1A3, CHSY1, COL12A1, COMT, DCN, FBN1, FOXO1, IHH, LRRK1, LUM, RUNX2, SMAD3, STK36, TGFB2,	17

Category	Function	Function Annotation	*P*-value	B-H *P*-value	Molecules	# Molecules
					THBS2, WNT10A	
Connective Tissue Development and Function	morphology	Morphology of limb bone	4.52E-05	2.74E-02	COL12A1, COMT, IHH, LRRK1, LUM, RUNX2, SMAD3, TGFB2	8
Connective Tissue Development and Function	lack	Lack of occipital bone	4.97E-05	3.02E-02	RUNX2, TGFB2	2

Category, refers to a general functional category (column A), which includes as subcategories Function (column B) and Function Annotation (column C); p-value, calculated by the Fisher’s Exact Test; B-H-p-value, refers to the -log Benjamini-Hochberg corrected P-value; Molecules, refers to the genes involved in each disease or bio function; # Molecules, refers to the number of genes involved in each function.

Supplementary Table 11.Distance from CCT-lead SNP to a Mendelian causing gene

lead SNP	Chr:bp	Mendelian disease causing gene	Mendelian disorder	Distance from lead SNP
rs96067	1:36571920	*COL8A2*	Fuchs endothelial dystrophy	6.1kb 5' of *COL8A2*
rs4846476	1:218526228	*TGFB2*	Loeys-Dietz syndrome	intronic
rs35028368	5:178671146	*ADAMTS2*	Ehlers-Danlos syndrome, type VIIC	intronic
rs3132303	9:137444298	*COL5A1*	Ehlers-Danlos syndrome, classic type	89kb 5' of *COL5A1*
rs7308752	12:91527181	*DCN-KERA*	DCN(corneal dystrophy) KERA (Cornea plana 2)	12kb 3' of *DCN* and 75kb 5' of *KERA*
rs8030753	15:48801935	*FBN1*	Marfan syndrome	intronic
rs12912010	15:67467143	*SMAD3*	Loeys-Dietz syndrome	intronic
rs4843040	15:85838636	*AGBL1*	Fuchs endothelial dystrophy	784kb from *AGBL1*
rs35193497	16:88324821	*ZNF469*	Brittle cornea syndrome	169kb 5' of *ZNF469*

Supplementary Table 12. Genes +/- 200kb of the lead SNP and genes included IPA analysis

Lead SNP				Genes in +/- 200 kb region of the lead SNP		
**SNP**	**Chr:bp**	**Nearest gene**	**dbSNP functional annotation**	**Coordinates of the region explored**	**Genes***	**Genes expressed in Cornea****
rs96067	1:36571920	*COL8A2*	intergenic	chr1:36371920-36771920	*AGO1,AK025726,AGO3,TEKT2,ADPRHL2,**COL8A2**,TRAPPC3,MAP7D1,THRAP3*	*AGO1,AGO3,TEKT2,ADPRHL2,COL8A2,TRAPPC3,MAP7D1,THRAP3*
rs4846476	1:218526228	*TGFB2*	intronic	chr1:218326228-218726228	*RRP15,LOC728463,**TGFB2***	*RRP15,**TGFB2***
rs115781177	2:33348494	*LTBP1*	intronic	chr2:33148494-33548494	*LINC00486,LOC100271832,**LTBP1**,5S_rRNA*	***LTBP1***
rs121908120	2:219755011	*WNT10A*	missense	chr2:219555011-219955011	*STK36,TTLL4,CYP27A1,AX748340,PRKAG3,WNT6,**WNT10A**,BC038542,CDK5R2,LINC00608,FEV,CRYBA2,MIR375,LOC100129175,CCDC108,IHH,MIR3131,NHEJ1*	*STK36,TTLL4,CYP27A1,PRKAG3,WNT6,**WNT10A**,CDK5R2,FEV,CRYBA2,CFAP65,IHH,NHEJ1*
rs4608502	2:228134155	*COL4A3*	intronic	chr2:227934155-228334155	*COL4A4,**COL4A3**,MFF,TM4SF20*	*COL4A4,**COL4A3**,MFF,TM4SF20*
3:136138073	3:136138073	*STAG1*	intronic	chr3:135938073-136338073	*PCCB,**STAG1***	*PCCB,**STAG1***
rs9822953	3:156472071	*TIPARPa*	intronic	chr3:156272071-156672071	*SSR3,TIPARP-AS1,**TIPARP**,LINC00886,PA2G4P4,AK094480,LEKR1*	*SSR3,**TIPARP,**LEKR1*
rs6445046	3:171933252	*FNDC3B*	intronic	chr3:171733252-172133252	***FNDC3B***	***FNDC3B***
3:177306757	3:177306757	*TBL1XR1b*	intergenic	chr3:177106757-177506757	*LINC00578*	

Lead SNP				Genes in +/- 200 kb region of the lead SNP		
**SNP**	**Chr:bp**	**Nearest gene**	**dbSNP functional annotation**	**Coordinates of the region explored**	**Genes***	**Genes expressed in Cornea****
rs28789690	4:149077899	*NR3C2*	intronic	chr4:148877899-149277899	*ARHGAP10,**NR3C2***	*ARHGAP10,NR3C2*
rs10471310	5:64548961	*ADAMTS6*	intronic	chr5:64348961-64748961	***ADAMTS6***	***ADAMTS6***
rs249767	5:141918585	*FGF1*	intergenic	chr5:141718585-142118585	*TRNA_Asp,Mir_186,**FGF1**, SNORA36*	***FGF1***
rs35028368	5:178671146	*ADAMTS2*	intronic	chr5:178471146-178871146	*ZNF354C,**ADAMTS2***	*ZNF354C,**ADAMTS2***
rs13191376	6:45522139	*RUNX2*	intergenic	chr6:45322139-45722139	*SUPT3H,**RUNX2***	*SUPT3H,**RUNX2***
rs1412710	6:75837203	*COL12A1*	intronic	chr6:75637203-76037203	***COL12A1,**COX7A2,TMEM30A,LOC100506804,FILIP1*	***COL12A1**,COX7A2,TMEM30A,FILIP1*
rs1931656	6:82610188	*FAM46A-IBTK*	intronic	chr6:82410188-82810188	***FAM46A,**BC038576*	***FAM46A***
6:169553553	6:169553553	*THBS2*	intergenic	chr6:169353553-169753553	*AF086258,**THBS2***	***THBS2***
7:66262284	7:66262284	*RABGEF1c*	intronic	chr7:66062284-66462284	*KCTD7,**RABGEF1**,GTF2IRD1P1,TMEM248,SBDS,TYW1*	*KCTD7,**RABGEF1**,TMEM248,SBDS,TYW1*
rs2106166	7:92668332	*SAMD9*	intergenic	chr7:92468332-92868332	***SAMD9,**SAMD9L,HEPACAM2,VPS50*	***SAMD9**,SAMD9L,HEPACAM2,VPS50*
rs3808520	8:23164773	*LOXL2*	intronic	chr8:22964773-23364773	*TNFRSF10C,TNFRSF10D, TNFRSF10A,LOC389641,CHMP7,R3HCC1,BC128546, **LOXL2**,LOC100507156,ENTPD4*	*TNFRSF10C,TNFRSF10D,TNFRSF10A,CHMP7, **LOXL2**,ENTPD4*
rs10429294	8:95969322	*NDUFAF6*	intergenic	chr8:95769322-96169322	*DPY19L4,INTS8,CCNE2,TP53INP1,**NDUFAF6**,AX747981,MIR3150B,MIR3150A,PLEKHF2*	*DPY19L4,INTS8,CCNE2,**NDUFAF6**,TP53INP1, PLEKHF2*

SNP	Lead SNP Chr:bp	Nearest gene	dbSNP functional annotation	Genes in +/- 200 kb region of the lead SNP Coordinates of the region explored	Genes*	Genes expressed in Cornea**
rs7026684	9:4215308	*GLIS3*	intronic	chr9:4015308-4415308	***GLIS3***,*Mir_320*	***GLIS3***
rs66720556	9:13559717	*MPDZ-NFIB*	intergenic	chr9:13359717-13759717	*FLJ41200*	
rs10980623	9:113660537	*LPAR1*	intronic	chr9:113460537-113860537	*MUSK*,***LPAR1***,*Y_RNA*	*MUSK*,***LPAR1***
rs3132303	9:137444298	*COL5A1*	intergenic	chr9:137244298-137644298	*RXRA*,*MIR4669*,***COL5A1***	*RXRA*,***COL5A1***
rs7040970	9:139859013	*LCN12*	intergenic	chr9:139659013-140059013	*TMEM141*,*CCDC183*,*RABL6*,*PHPT1*,*MAMDC4*,*EDF1*,*TRAF2*,*FBXW5*,*C8G*,***LCN12***,*PTGDS*,*LCNL1*,*PAXX*,*CLIC3*,*ABCA2*,*C9orf139*,*FUT7*,*NPDC1*,*ENTPD2*,*SAPCD2*,*UAP1L1*,*MAN1B1-AS1*,*MAN1B1*,*DPP7*,*GRIN1*	*TMEM141*,*CCDC183*,*RABL6*,*PHPT1*,*MAMDC4*,*EDF1*,*TRAF2*,*FBXW5*,*C8G*,***LCN12***,*PTGDS*,*LCNL1*,*PAXX*,*CLIC3*,*ABCA2*,*C9orf139*,*FUT7*,*NPDC1*,*ENTPD2*,*SAPCD2*,*UAP1L1*,*MAN1B1-AS1*,*MAN1B1*,*DPP7*,*GRIN1*
rs35809595	10:63831928	*ARID5B*	intronic	chr10:63631928-64031928	***ARID5B***,*MIR548AV*,*RTKN2*,*ZNF365*	*RTKN2*
rs2419835	10:115296564	*HABP2*	intergenic	chr10:115096564-115496564	***HABP2***,*NRAP*,*CASP7*	***HABP2***,*NRAP*,*CASP7*
rs4938174	11:110913240	*ARHGAP20-C11orf53*	intergenic	chr11:110713240-111113240	no genes in +/- 200KB window	
rs56009602	11:130289612	*ADAMTS8*	intronic	chr11:130089612-130489612	*ZBTB44*,*AX747213*,*BC144418*,*BC144419*,***ADAMTS8***,*ADAMTS15*	*ZBTB44*,***ADAMTS8***,*ADAMTS15*
rs7308752	12:91527181	*LUM-DCN*	intergenic	chr12:91327181-91727181	*LINC00615*,*CCER1*,*EPYC*,*KERA*,***LUM*,*DCN***	*EPYC*,*KERA*,***LUM*,*DCN***
rs11553764	12:104415244	*GLT8D2*	5'-UTR	chr12:104215244-104615244	*NT5DC3*,*GNN*,*HSP90B1*,*MIR3652*,*C12orf73*,*TDG*,***GLT8D2***,*DL490275*,*HCFC2*,*HC*	*NT5DC3*,*HSP90B1*,*TDG*,***GLT8D2***,*HCFC2*,*NFYB*,*TXNRD1*

	Lead SNP			Genes in +/- 200 kb region of the lead SNP		
SNP	Chr:bp	Nearest gene	dbSNP functional annotation	Coordinates of the region explored	Genes*	Genes expressed in Cornea**
					FC2,NFYB	
rs10161679	13:23243645	*FGF9-SGCGd*	intergenic	chr13:23043645-23443645	*BC048997*	
13:41112152	13:41112152	*FOXO1*	intergenic	chr13:40912152-41312152	*LINC00598,7SK,**FOXO1**,MIR320D1,MRPS31*	***FOXO1**,MRPS31*
rs56223983	14:81814754	*STON2*	intronic	chr14:81614754-82014754	*GTF2A1,SNORA79,BC040628,**STON2**,SEL1L*	*GTF2A1,**STON2**,SEL1L*
rs785422	15:30173885	*TJP1*	intergenic	chr15:29973885-30373885	***TJP1***	***TJP1***
rs8030753	15:48801935	*FBN1*	intronic	chr15:48601935-49001935	*DUT,**FBN1***	*DUT,**FBN1***
rs12912010	15:67467143	*SMAD3*	intronic	chr15:67267143-67667143	*Mir_1302,**SMAD3**,AAGAB,IQCH*	***SMAD3**,AAGAB,IQCH*
rs4843040	15:85838636	*AKAP13e*	intergenic	chr15:85638636-86038636	*PDE8A,DQ596274,BC096759,LOC642423,DQ574760,AK308544,GOLGA6L4,AK301968,DQ582071,DQ582071,**AKAP13***	*PDE8A,**AKAP13***
rs930847	15:101558562	*LRRK1*	intronic	chr15:101358562-101758562	*LOC145757,ALDH1A3,AK126286,AF198444,BC073817,**LRRK1**,CHSY1*	*ALDH1A3,**LRRK1**,CHSY1*
rs35193497	16:88324821	*ZNF469*	intergenic	chr16:88124821-88524821	*AK126852,**ZNF469**,ZFPM1*	***ZNF469**,ZFPM1*
rs4792535	17:14565130	*HS3ST3B1*	intergenic	chr17:14365130-14765130		
rs8133436	21:47519535	*COL6A2*	intronic	chr21:47319535-47719535	*PCBP3,COL6A1,**COL6A2**, DKFZ, p586E1322,FTCD,SPATC1L,LSS,MCM3AP-AS1,MCM3AP,YBEY*	*COL6A1,**COL6A2**,FTCD,SPATC1L,LSS,MCM3AP-AS1,MCM3AP*

	Lead SNP			Genes in +/- 200 kb region of the lead SNP		
**SNP**	**Chr:bp**	**Nearest gene**	**dbSNP functional annotation**	**Coordinates of the region explored**	**Genes***	**Genes expressed in Cornea****
rs71313931	22:19960184	*ARVCF*	intronic	chr22:19760184-20160184	*TBX1,GNB1L,C22orf29,TXNRD2,COMT,MIR4761,**ARVCF**,TANGO2,MIR185,DGCR8,MIR3618,MIR1306,TRMT2A,RANBP1,ZDHHC8,LOC388849*	*TANGO2,DGCR8,TRMT2A,ZDHHC8*

[a] The lead SNP is located in a validated non-coding mRNA, *LINC00578*; [b] In Lu et al this locus was reported as two loci (*VKORC1L1* and *C7orf42*); [c] The lead SNP is located in the pseudogene *ADAMTS7P4*

* Genes (including non-coding RNA) were retrieved from the UCSC. Column F "Coordinates of the region explored" shows the coordinates used to retrieve the genes.

** Genes expressed in Cornea. Column H shows the genes that showed expression levels in the cornea (i.e., PLIER > 3) in the ocular tissue database

Supplementary Figures

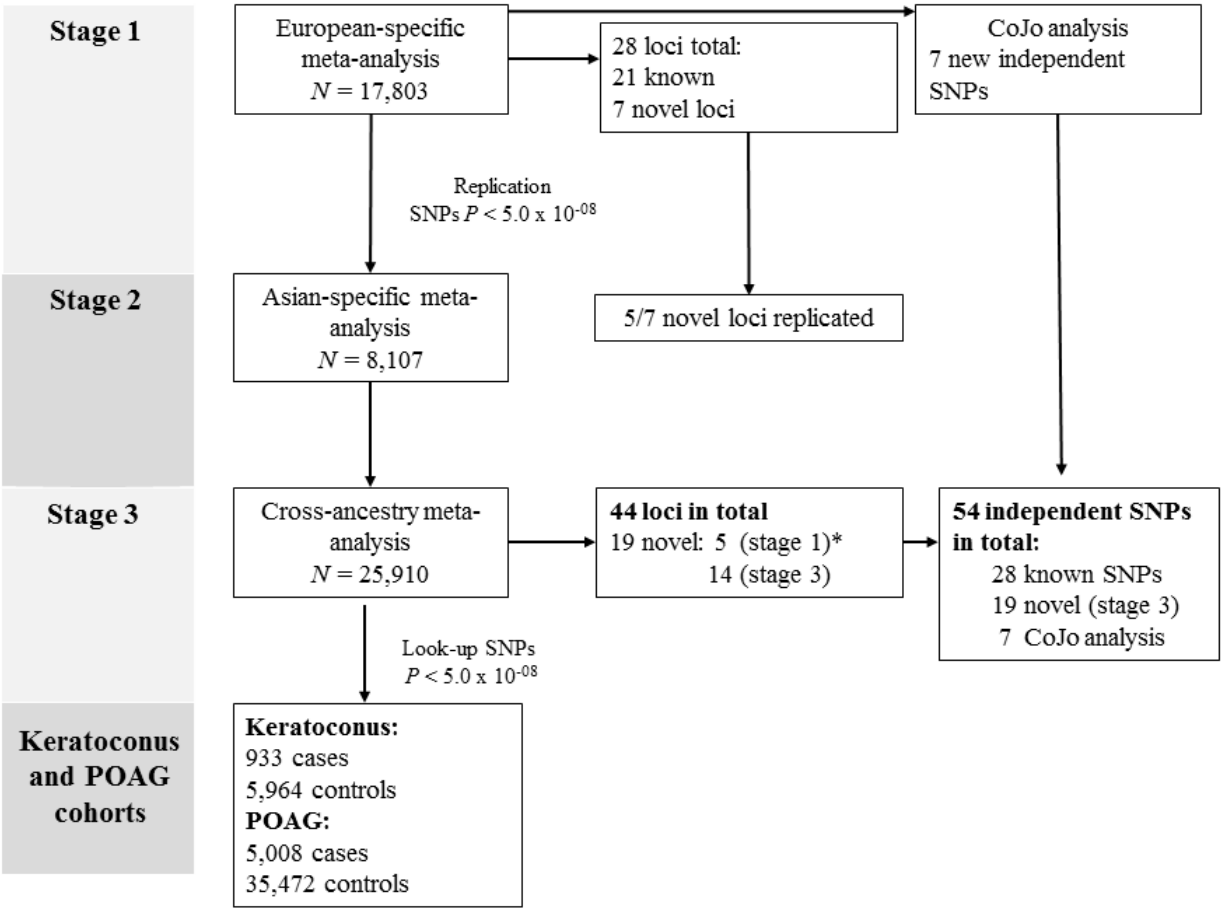

Supplementary Figure 1. Study design and main findings

We conducted a three-stage GWAS meta-analysis of CCT. Stage 1 included 14 studies of European ancestry. In stage 2, we examined the 28 lead SNPs from stage 1 in the Asian-specific meta-analysis. In stage 3, we conducted a cross-ancestry meta-analysis in more than 25,000 individuals to detect additional CCT loci. We then investigated whether the CCT-associated SNPs influence susceptibility to keratoconus and POAG. In total, we identified 44 loci harbouring 54 independent SNPs. POAG, Primary open-angle glaucoma; SNPs, single

nucleotide polymorphisms; CoJo, conditional and joint multiple-SNP. *The associated SNP in *LTBP1* (found in stage 1) is not polymorphic in 1000 Genomes East Asians and hence is a European ancestry-specific locus.

Supplementary Figure 2. A) QQ plot and B) Manhattan plot of CCT in the European-specific meta-analysis

a)

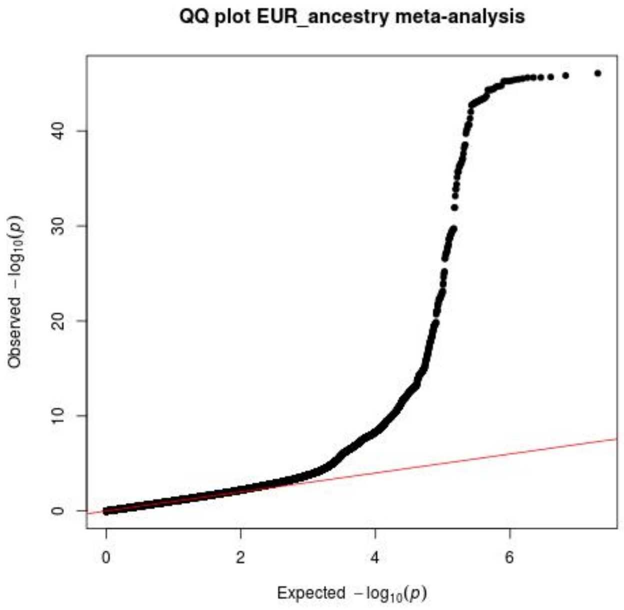

Figure 2a. Quantile-Quantile (QQ) plot of observed versus expected p-values on a log scale for CCT in the European-specific meta-analysis. The red line represents the null hypothesis of no true association.

b)

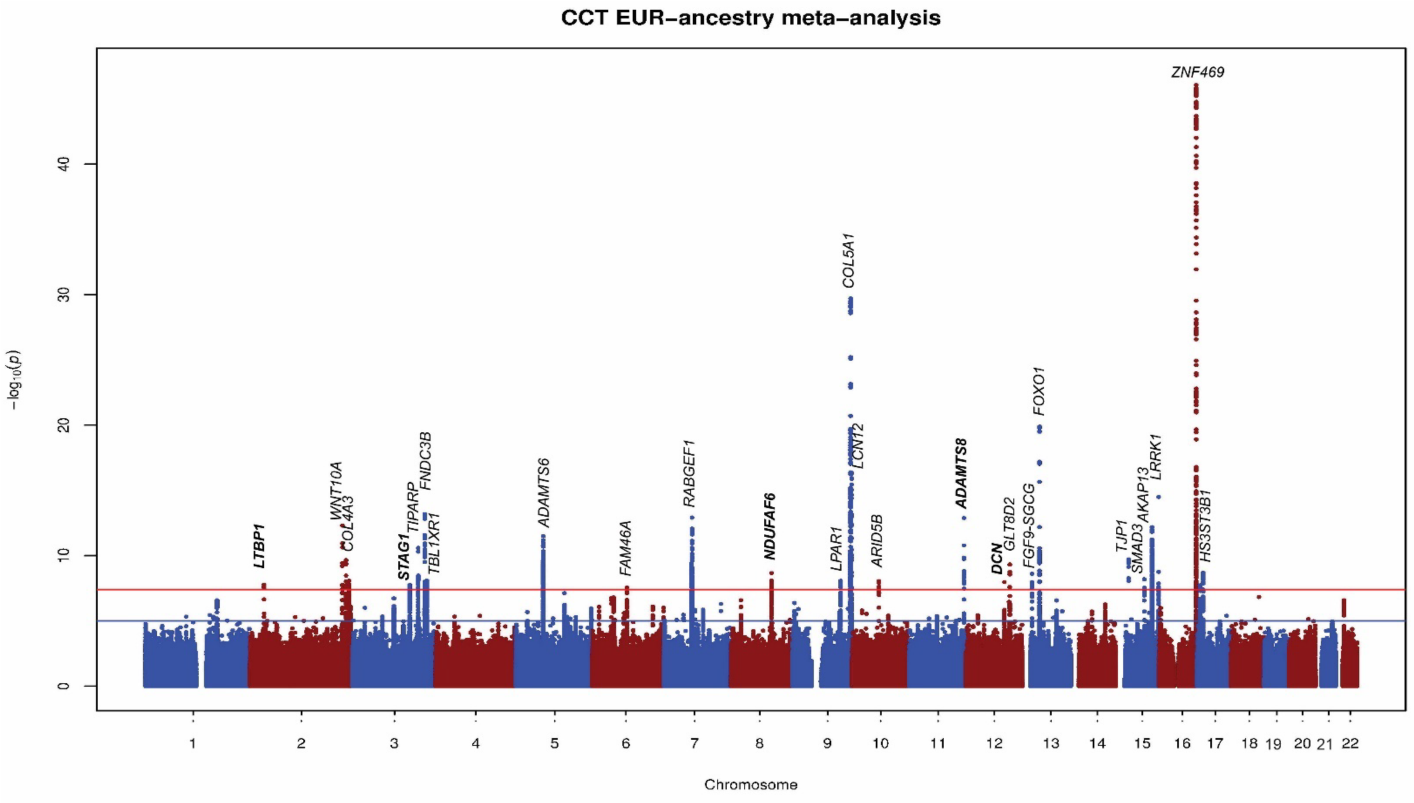

Figure 2b. Manhattan plot of the GWAS meta-analysis for CCT in the European-specific analysis (n = 17,803). The plot shows −log10-transformed *P*-values for all SNPs. The red dotted horizontal line represents the genome-wide significance threshold of $P < 5.0 \times 10^{-8}$; the blue dotted line indicates a *P*-value of 1×10^{-5}.

Supplementary Figure 3. A) Q-Q plot and B) Manhattan plot of CCT in the Asian-specific meta-analysis

a)

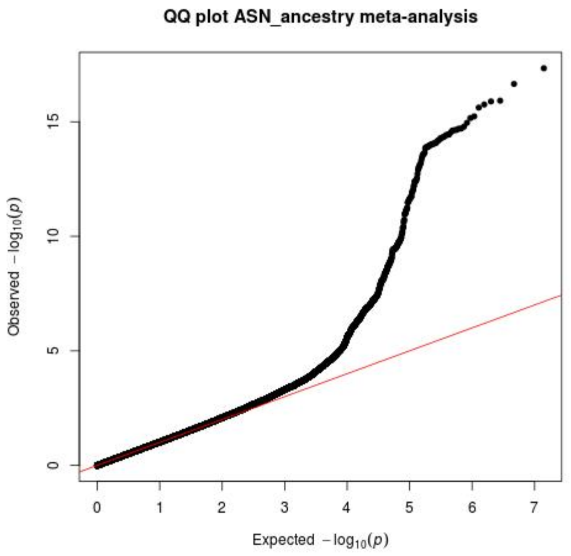

Figure 3a. Quantile-Quantile (QQ) plot of observed versus expected p-values on a log scale for CCT in the Asian-specific meta-analysis. The red line represents the null hypothesis of no true association.

b)

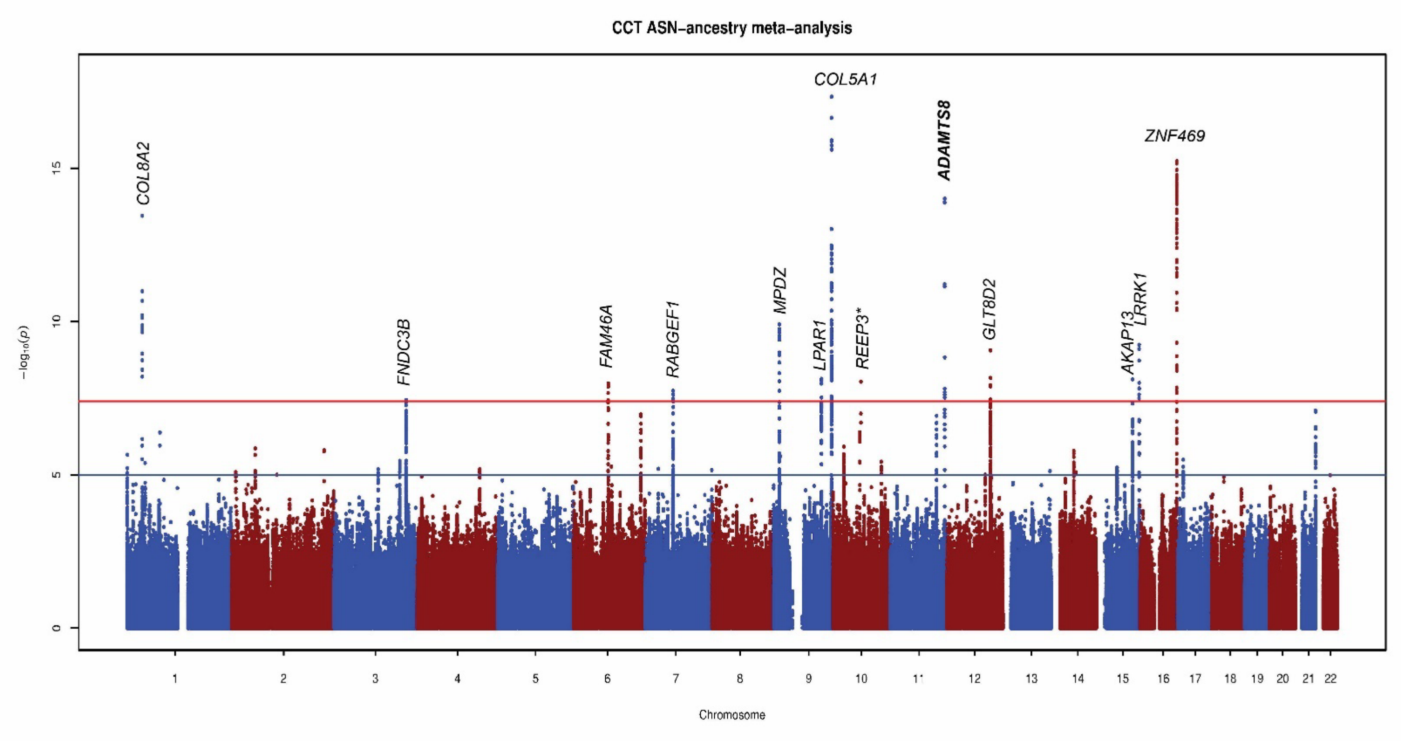

Figure 3b. Manhattan plot of the GWAS meta-analysis for CCT in the Asian-specific analysis (n = 8,107). The plot shows −log10-transformed *P*-values for all SNPs. The red dotted horizontal line represents the genome-wide significance threshold of $P < 5.0 \times 10^{-8}$; the blue dotted line indicates a *P*-value of 1×10^{-5}.

Supplementary Figure 4. Q-Q plot of CCT in the cross-ancestry meta-analysis

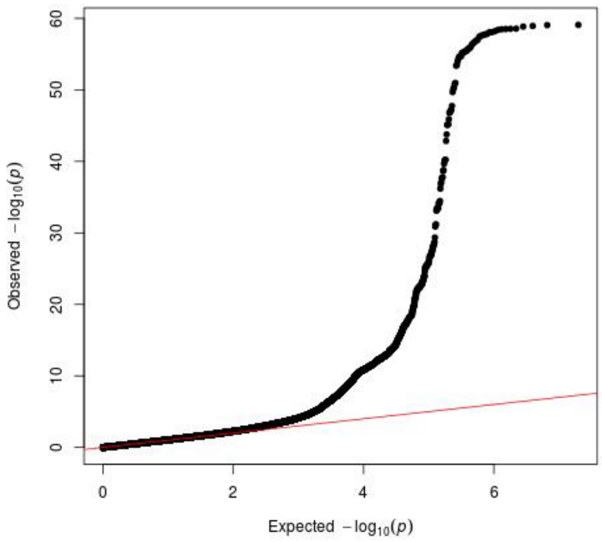

Figure 4. Quantile-Quantile (QQ) plot of observed versus expected p-values on a log scale for CCT in the cross-ancestry meta-analysis. The red line represents the null hypothesis of no true association.

Supplementary Figure 5. Regional association and recombination plots of CCT loci

Regional association and recombination rate plots for all 44 CCT loci identified in the cross-ancestry meta-analysis. A) European-ancestry and B) Asian-ancestry. Plots are centered on the most significant SNP at each locus (from the cross-ancestry meta-analysis) and flanked by the meta-analysis results for SNPs in the 400-kb region surrounding it. For each locus, the lead SNP (lowest *P*-value) is depicted as a purple dot; other SNPs are shaded according to their pairwise correlation (R2) with the lead SNP. The blue line represents the estimated recombination rates; the gene annotations are shown below the figure. Plots were created with Locuszoom (Locuszoom (http://csg.sph.umich.edu/locuszoom). Figures are shown in the same order as in main **Table 1**.

rs96067 a. b.

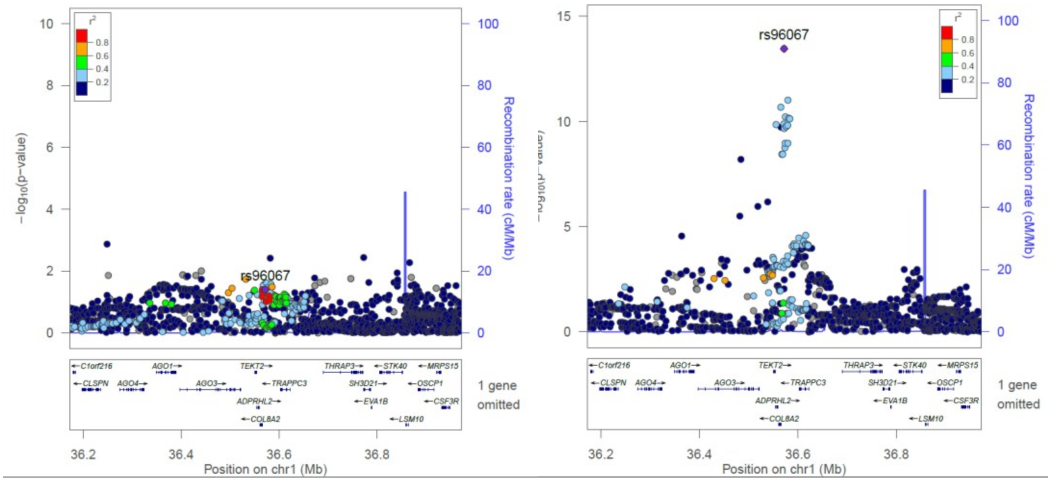

rs4846476 a. b.

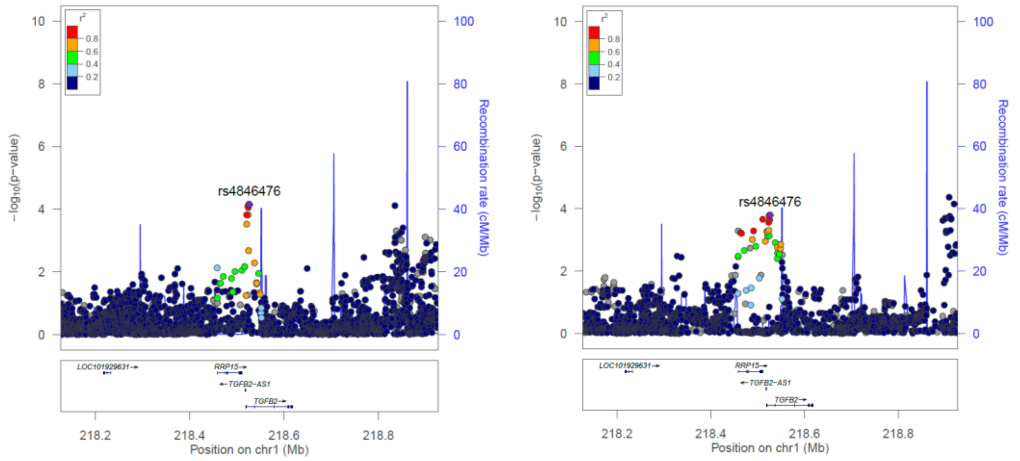

rs115781177 a. b.

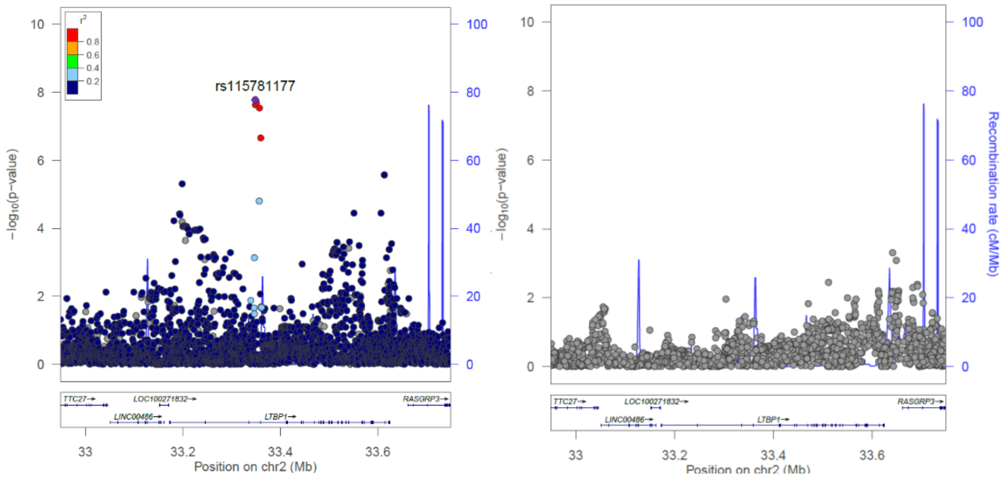

rs1219 08120 a. b.

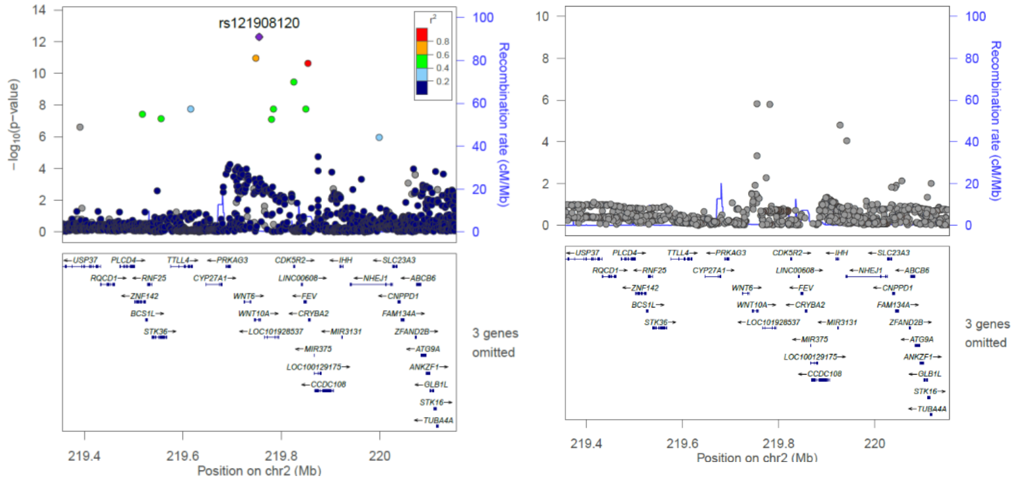

rs4608502 a.

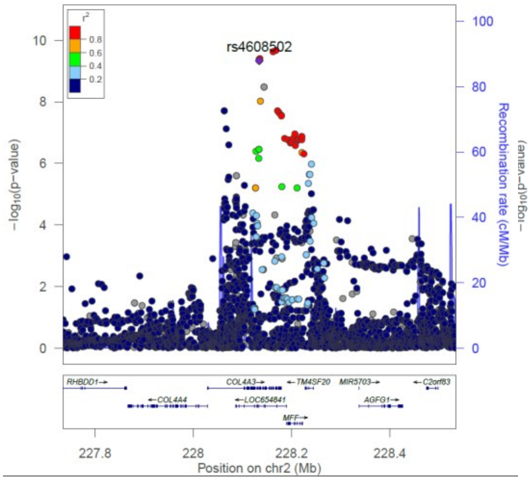

b.

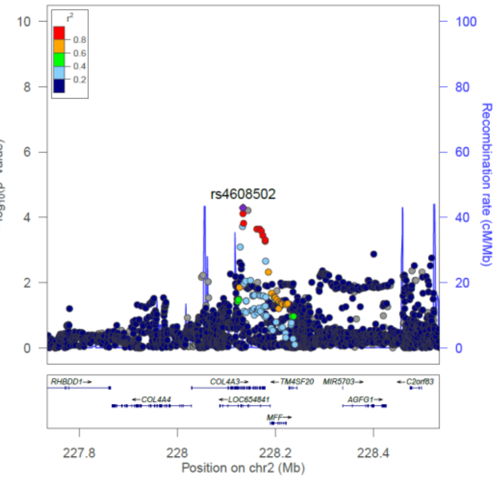

STAG1: a.

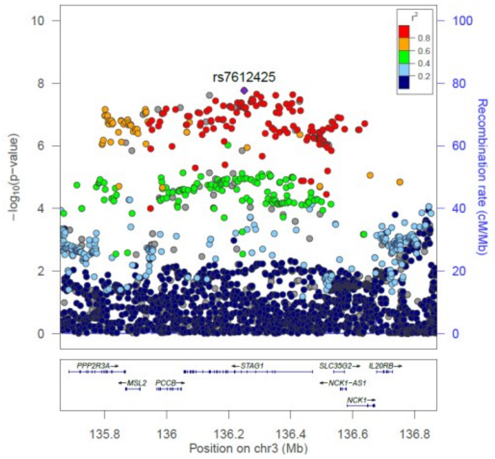

b.

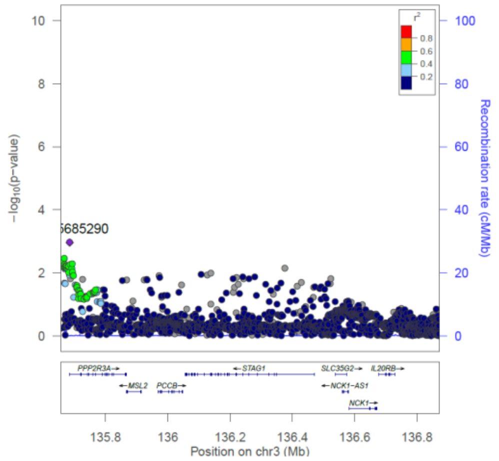

rs9822953 a.

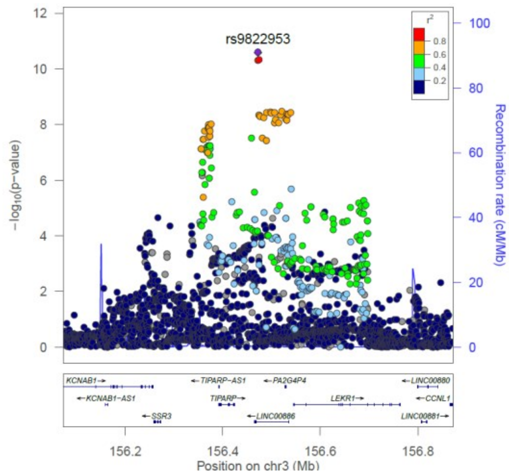

b.

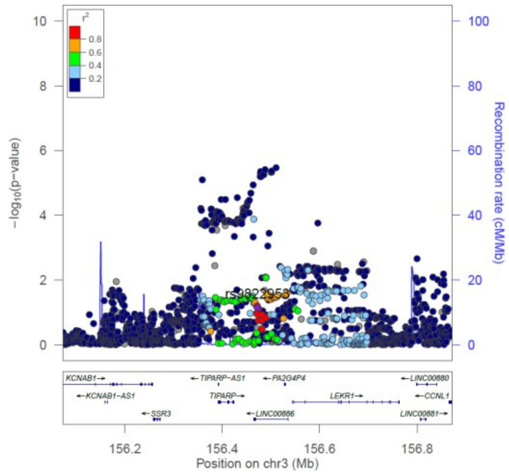

rs6445046 a.

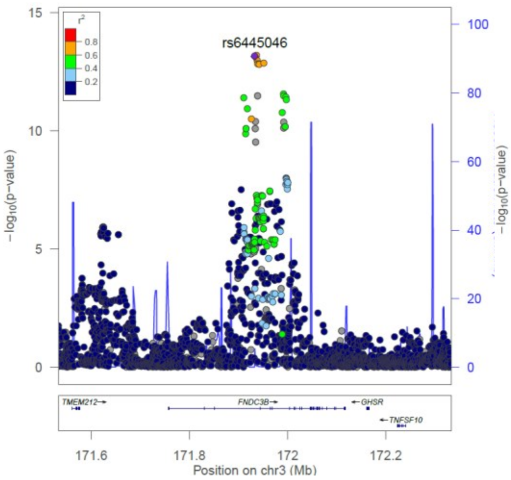

b.

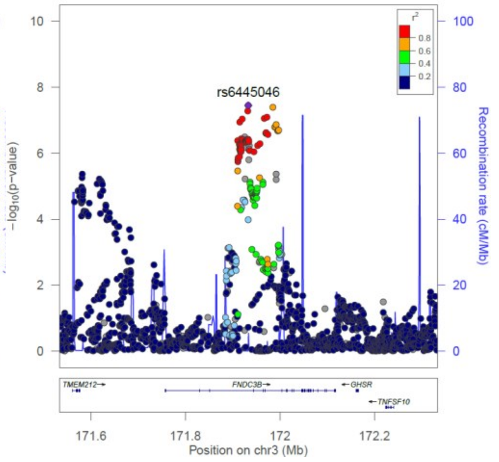

LINC00578 a.

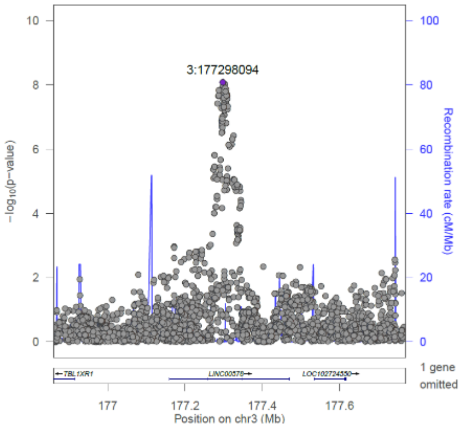

b.

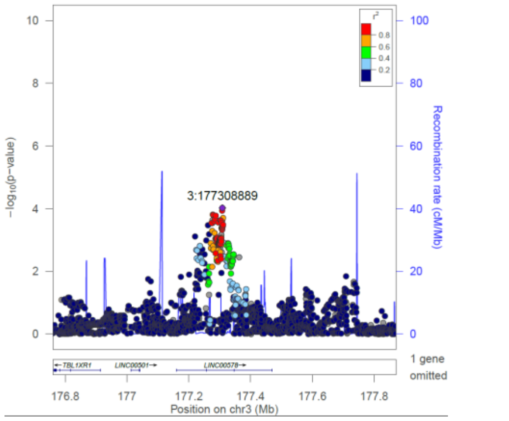

rs28789690 a.

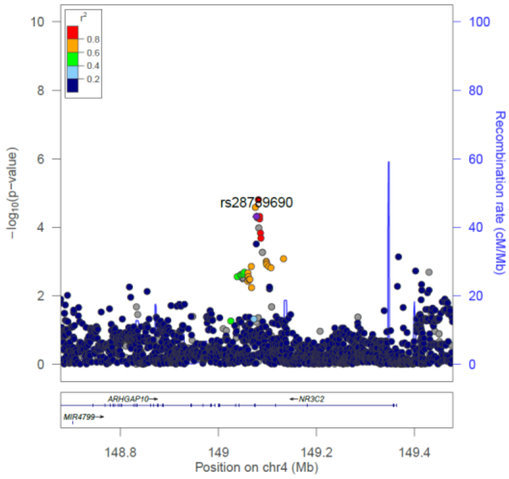

b.

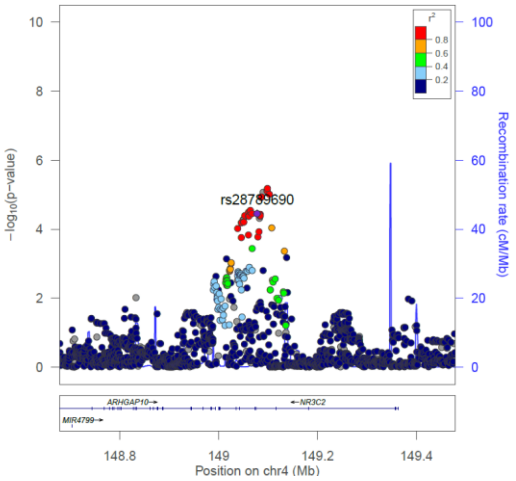

rs10471310 a.

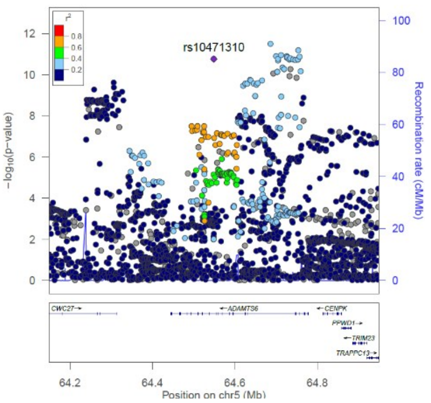

b.

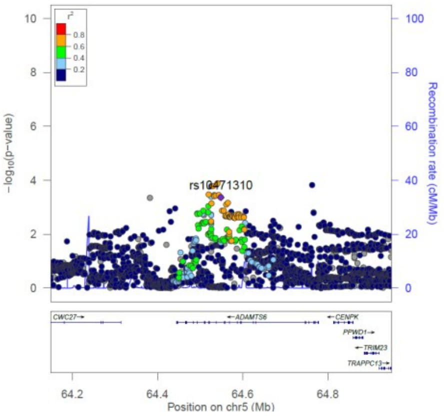

rs249767 a.

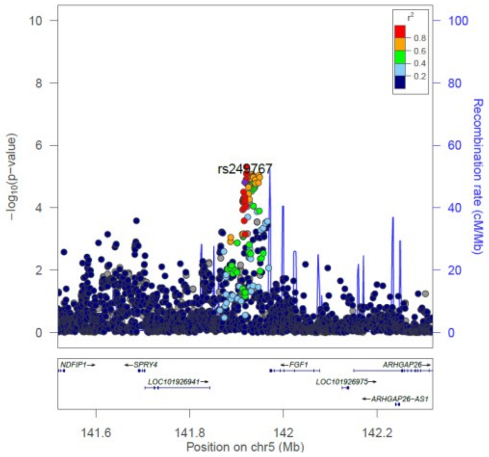

b.

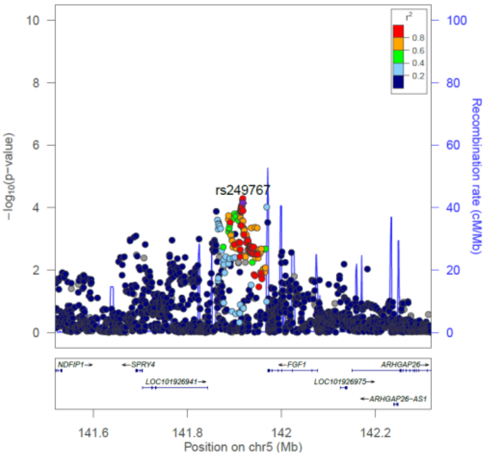

rs35028368 a.

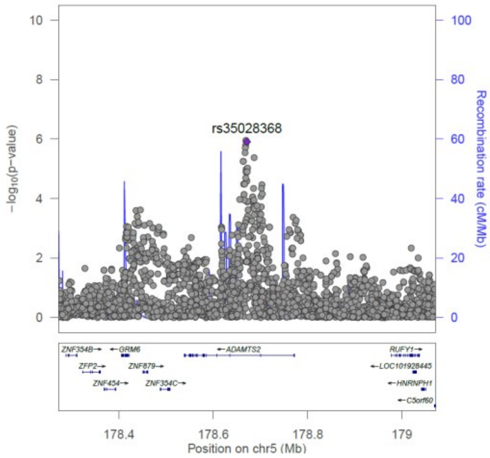

b.

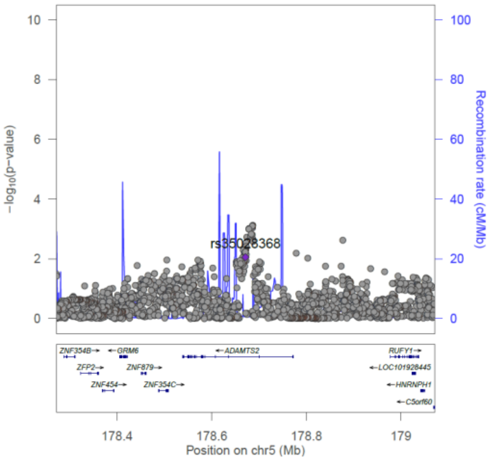

rs13191376 a.

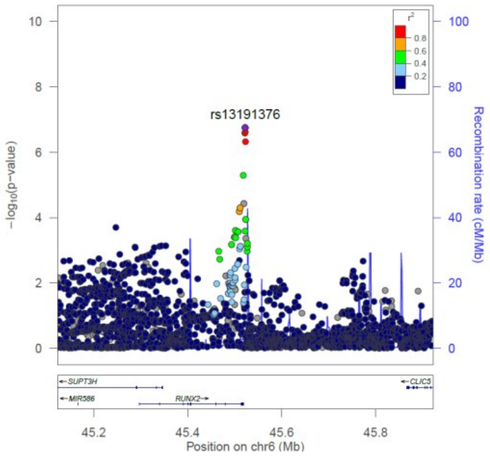

b.

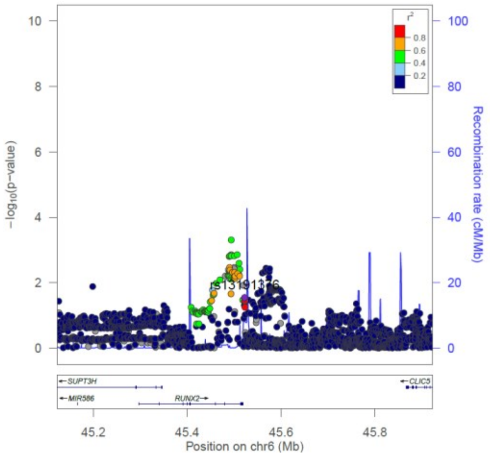

rs1412710 a.

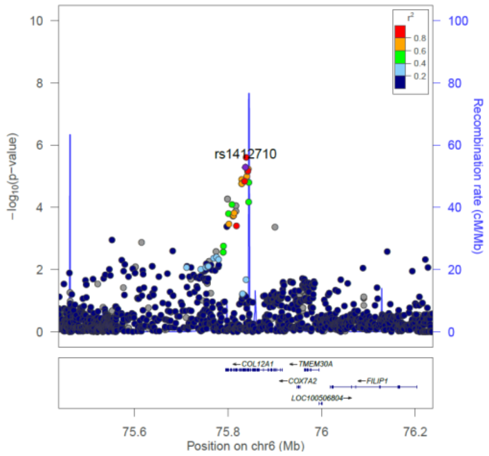

b.

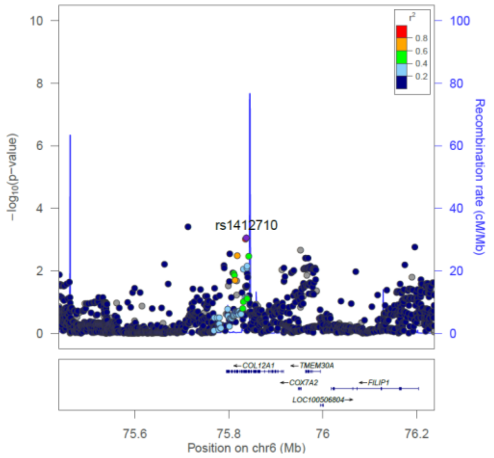

rs1931656 a.

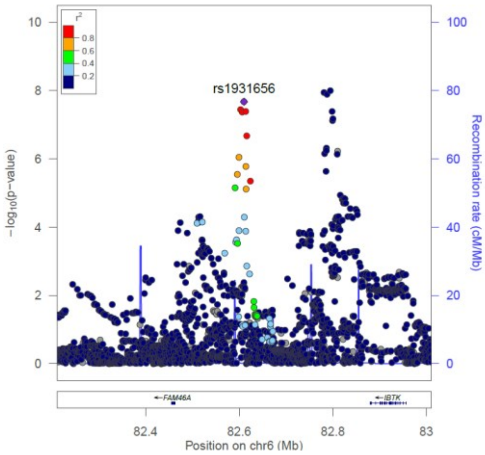

b.

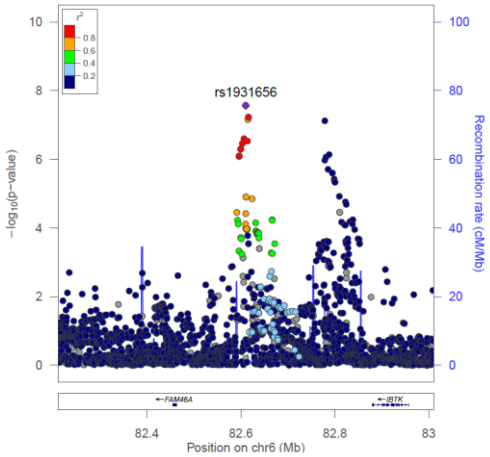

THBS2 a.

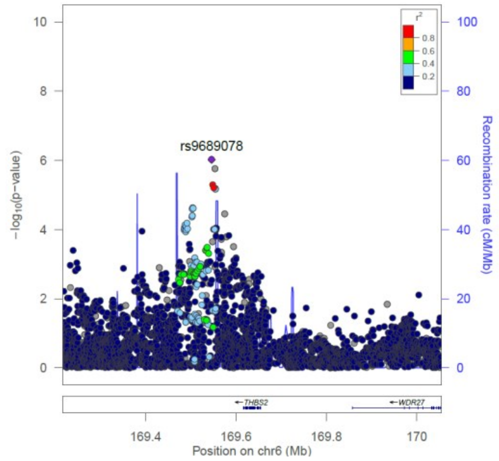

b.

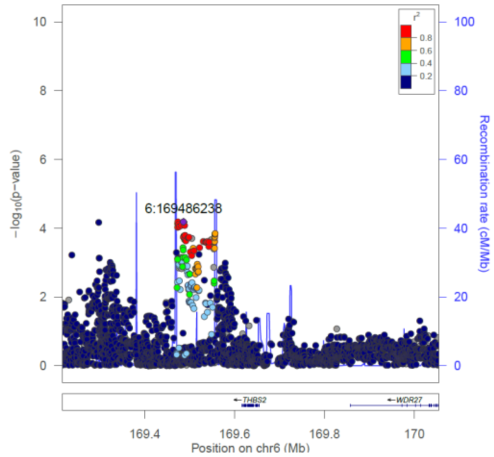

RASGEF1 a.

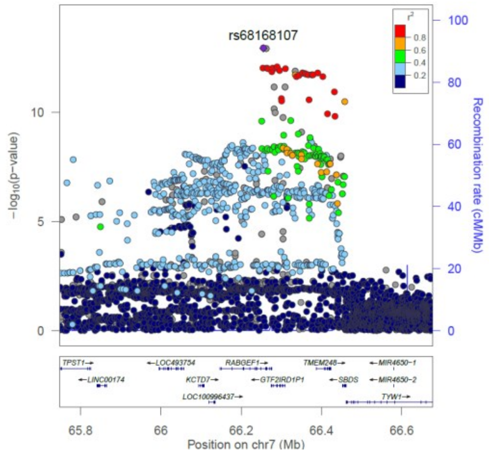

b.

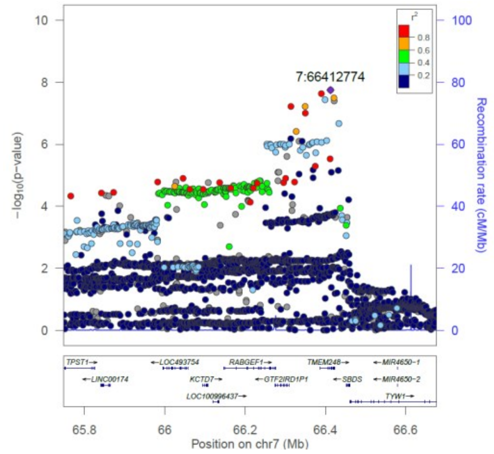

rs2106166 a.

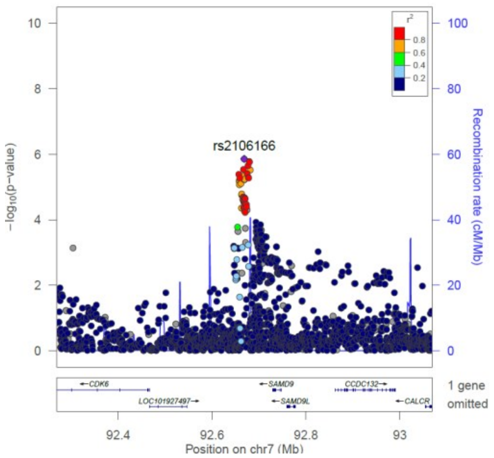

b.

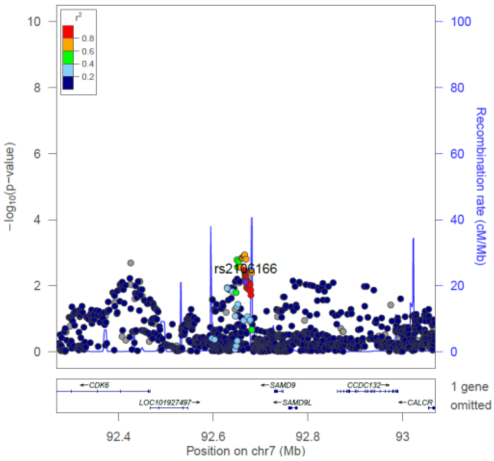

rs3808520 a.

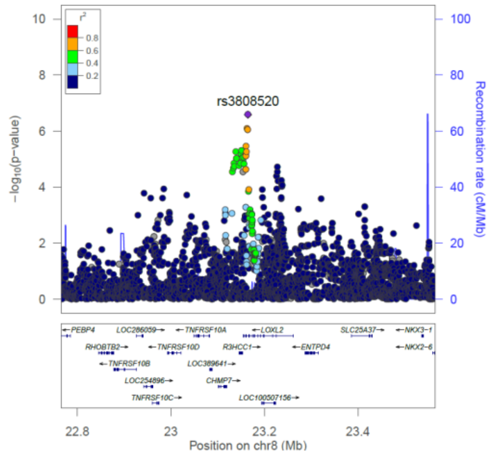

b.

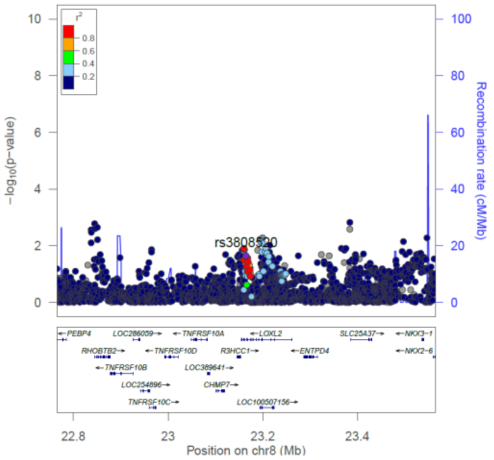

rs10429294 a.

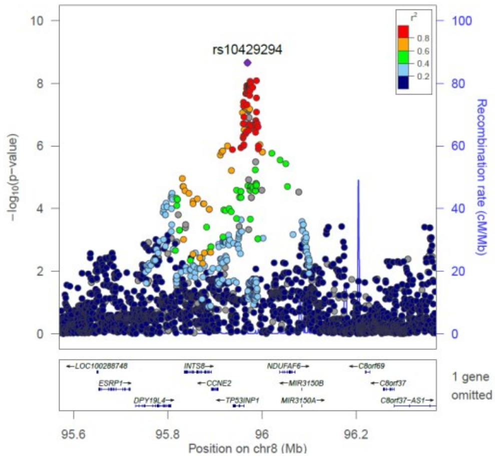

b.

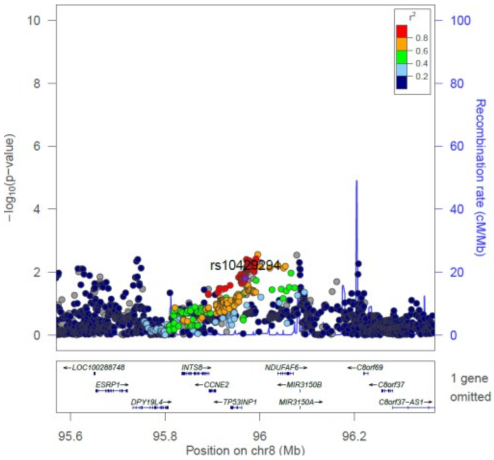

rs7026684 a.

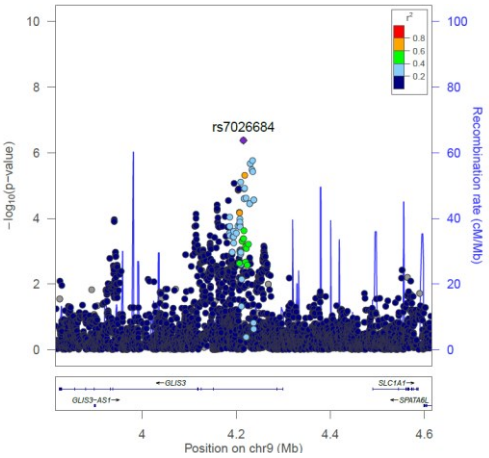

b.

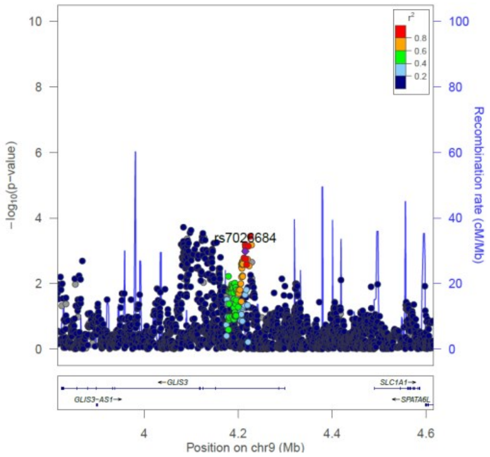

rs66720556 a.

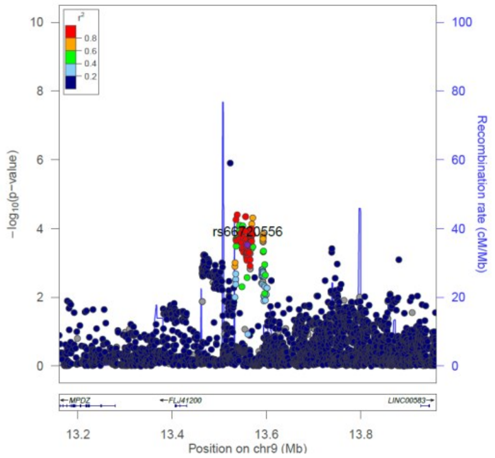

b.

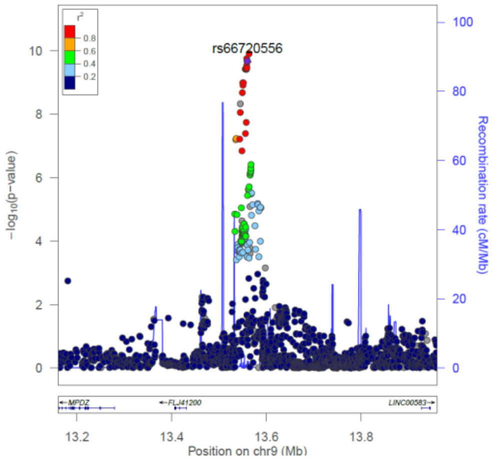

rs10980623 a.

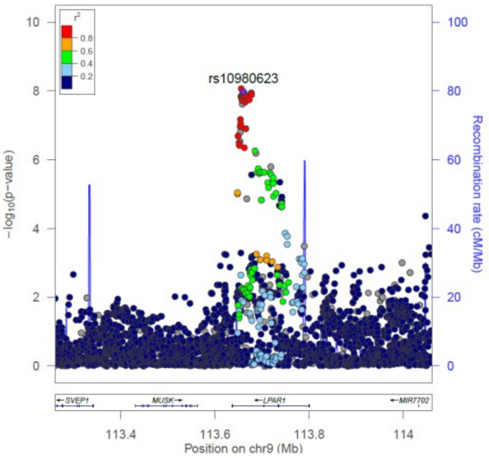

b.

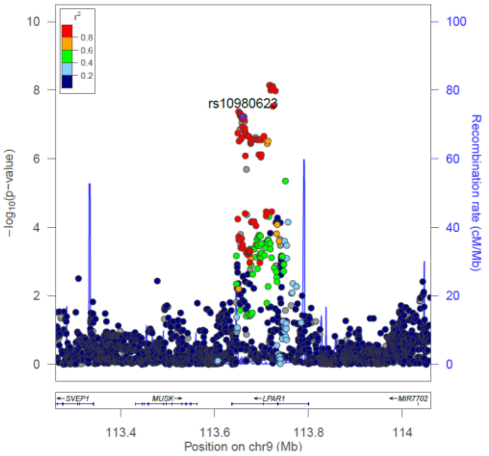

rs3132303 a.

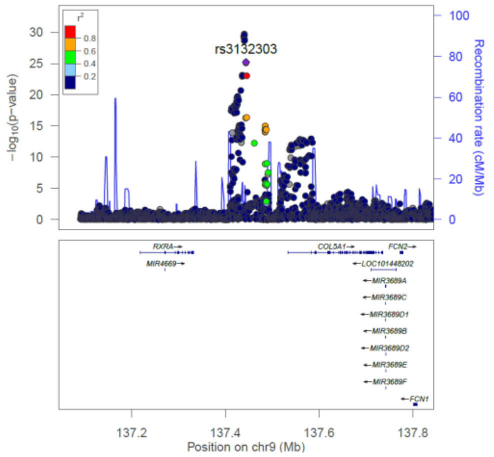

b.

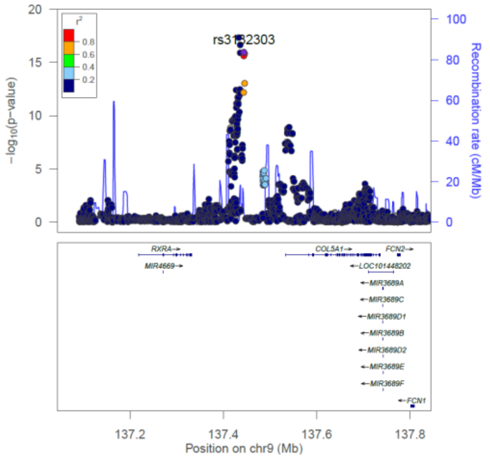

rs7040970 a.

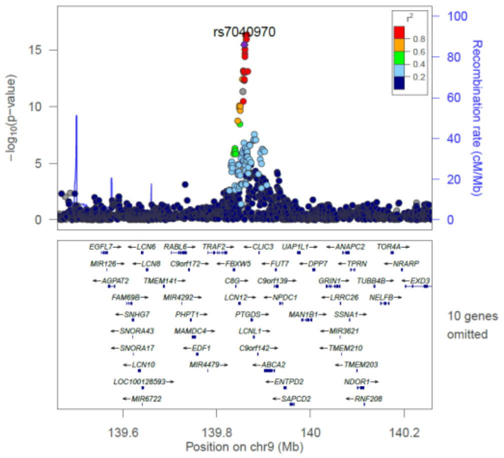

b.

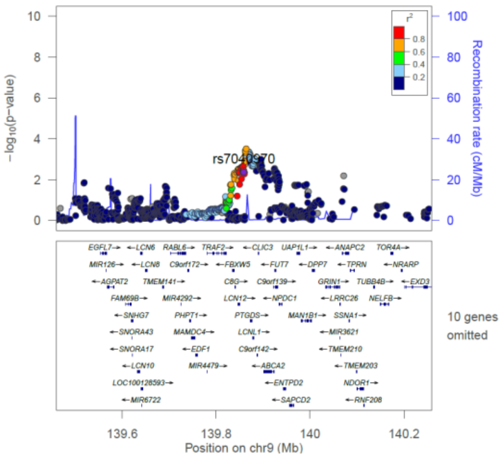

rs35809595 a.

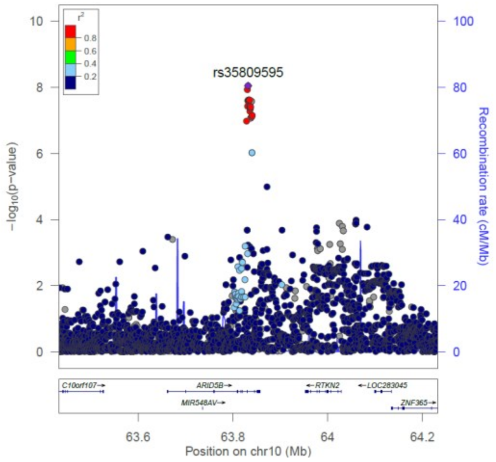

b.

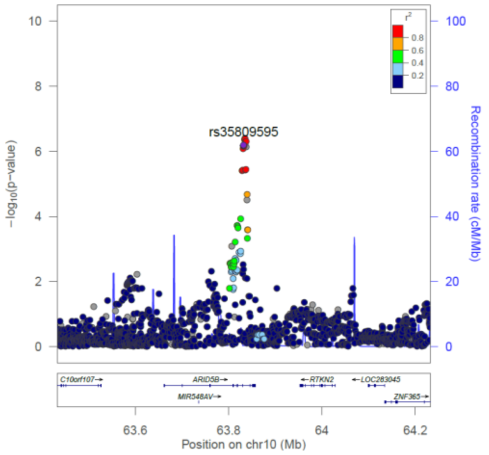

rs2419835 a.

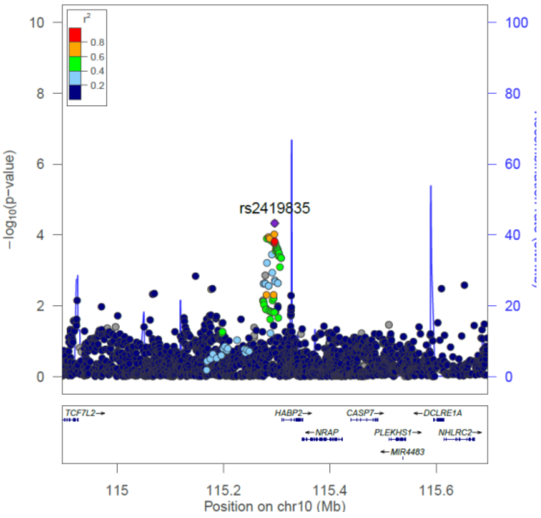

b.

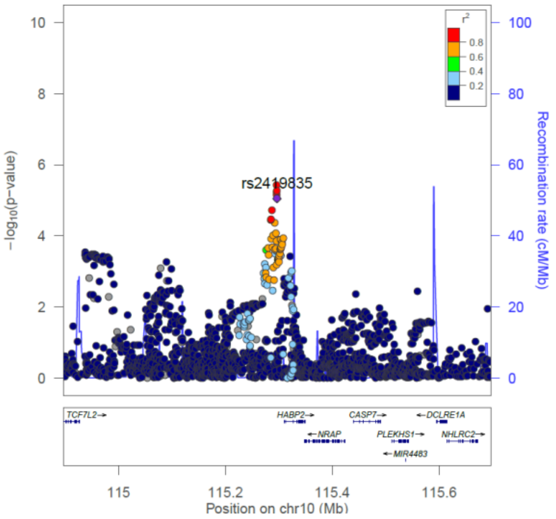

rs4938174 a.

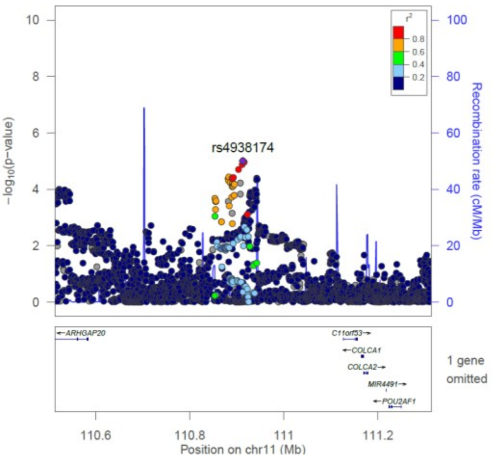

b.

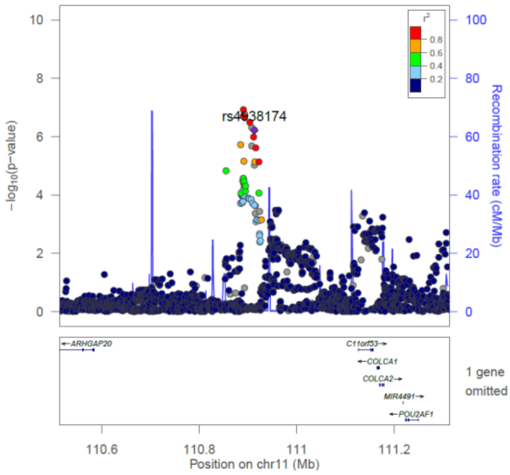

rs56009602 a.

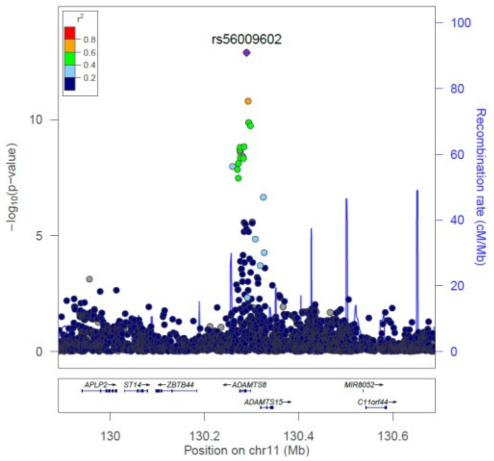

b.

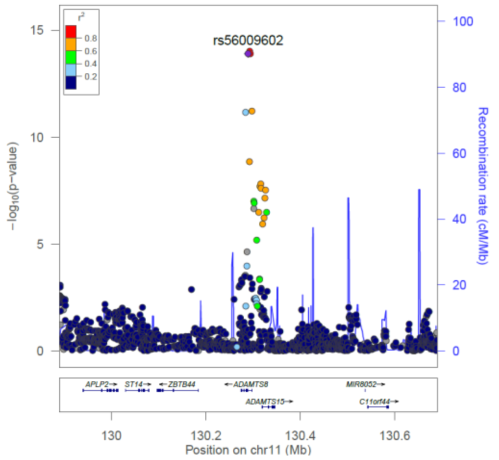

rs7308752 a.

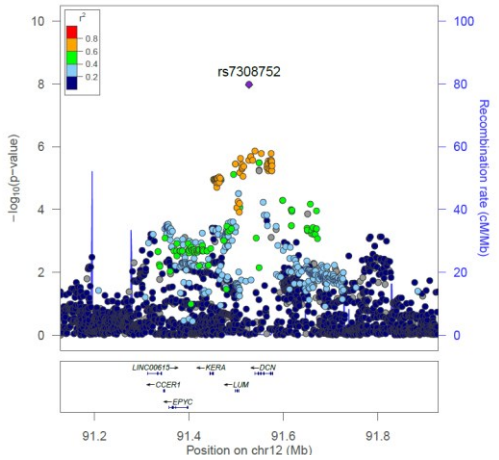

b.

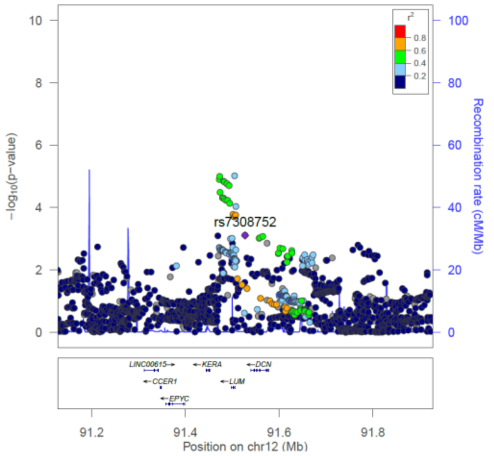

rs11553764 a.

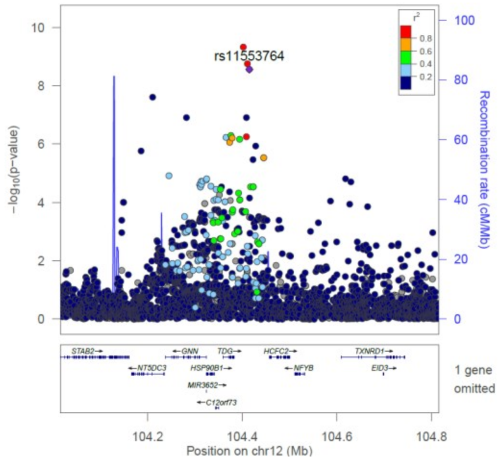

b.

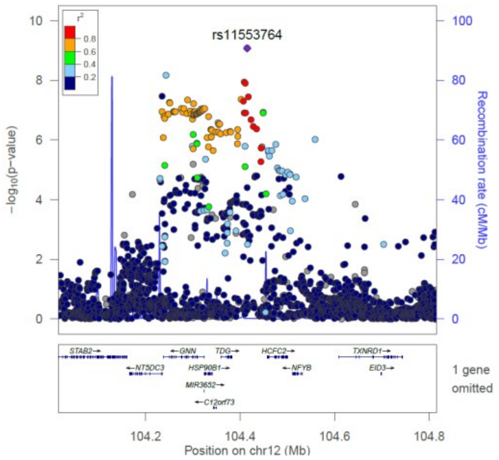

rs10161679 a.

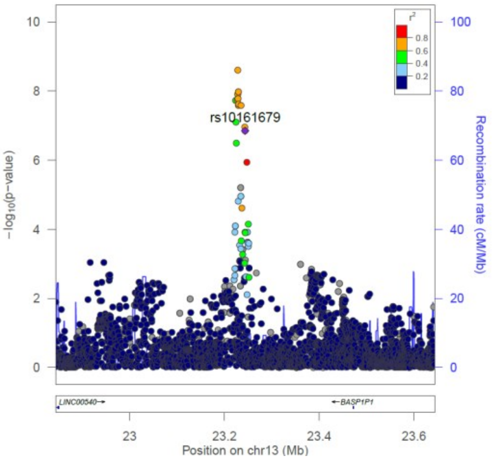

b.

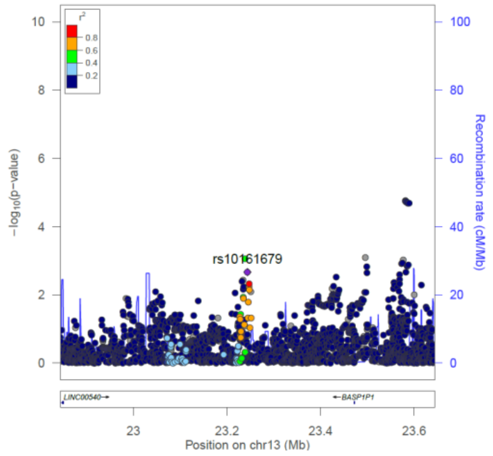

FOXO1 a.

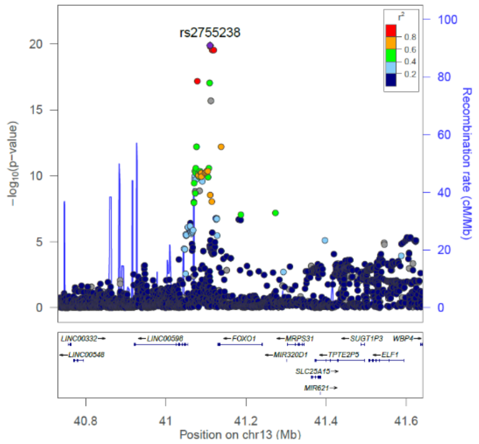

b.

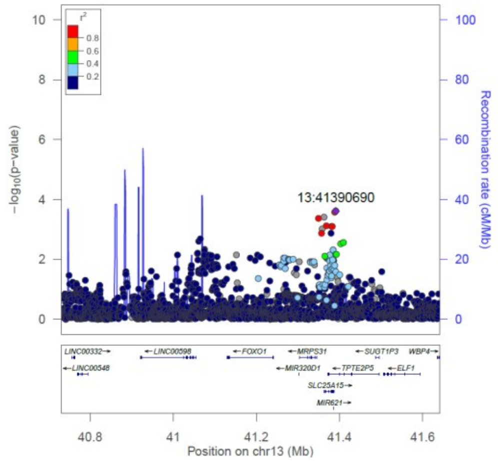

rs56223983 a.

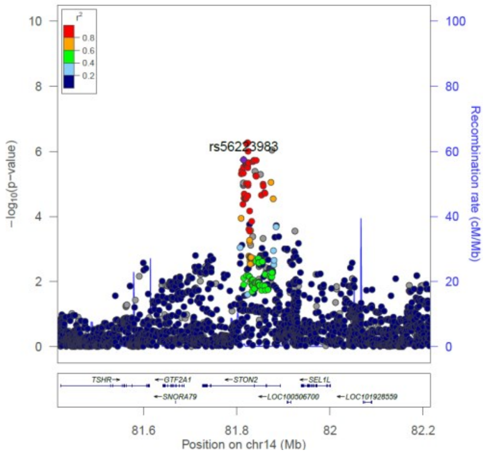

b.

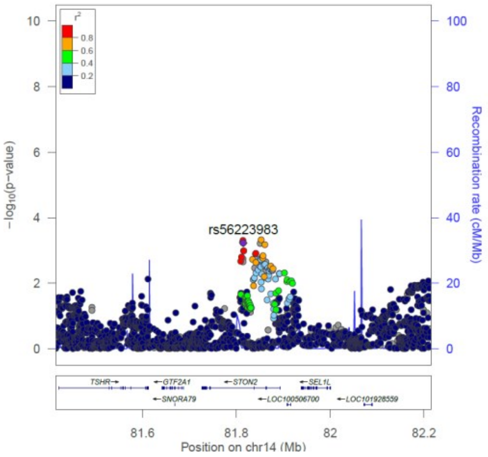

rs785422 a.

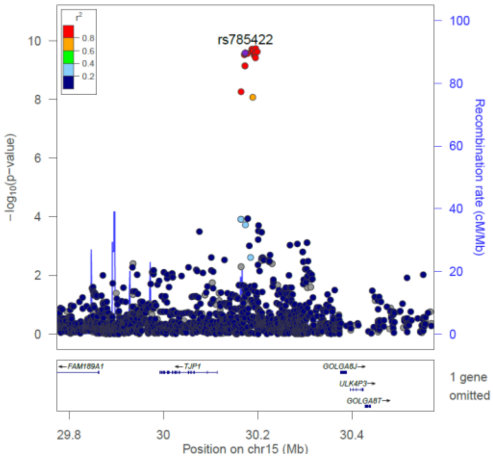

b.

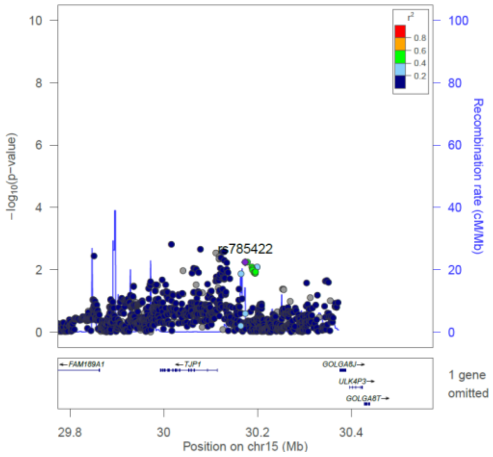

rs8030753 a.

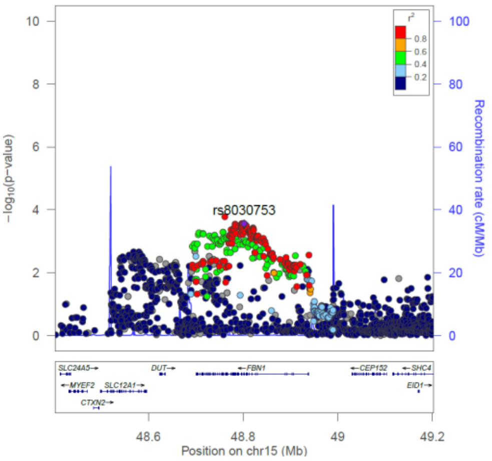

b.

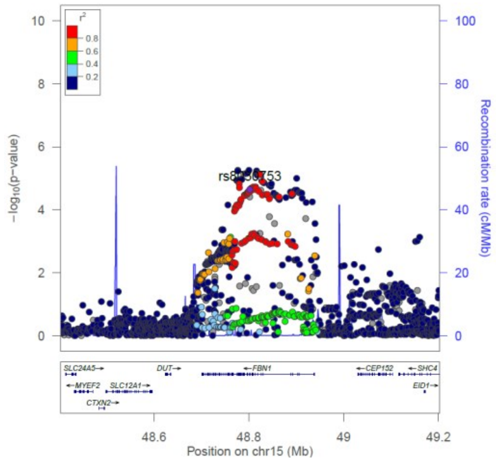

rs12912010 a.

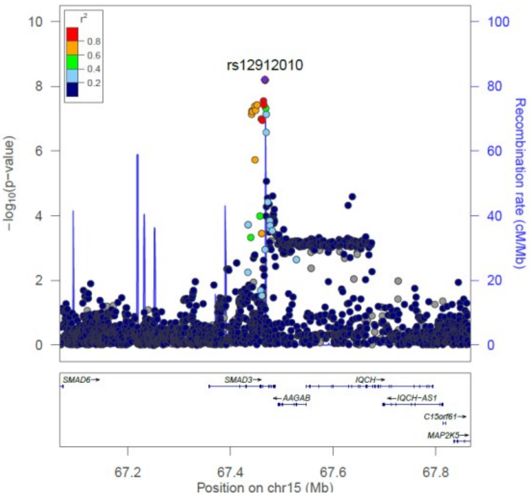

b.

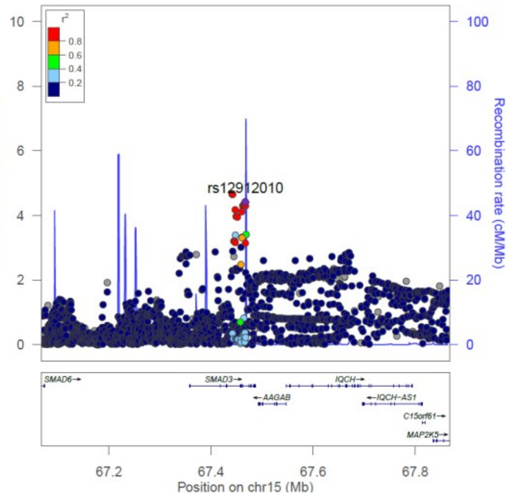

rs4843040 a.

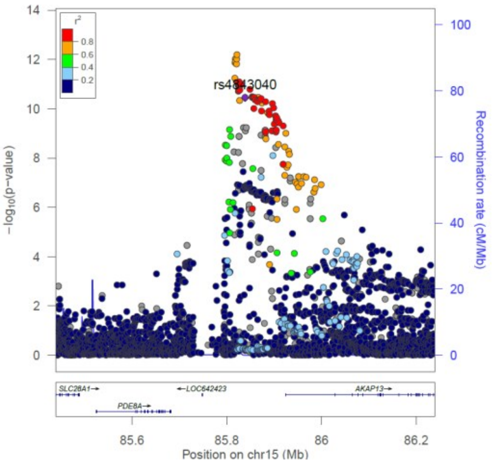

b.

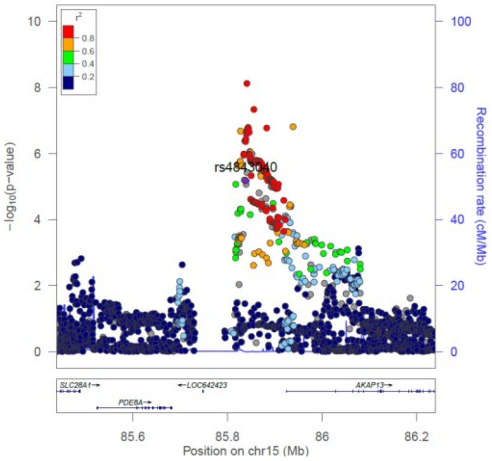

rs930847 a.

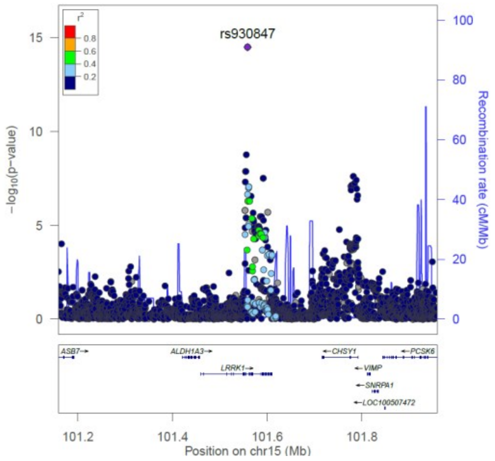

b.

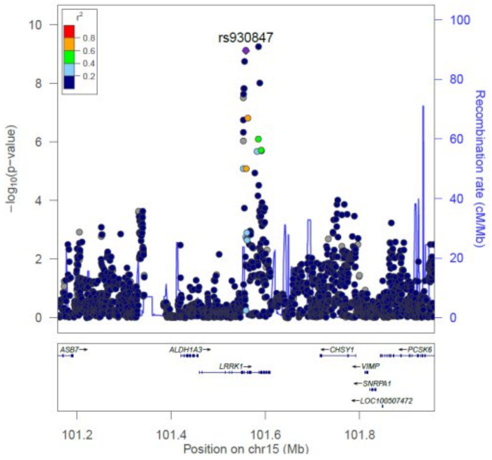

rs35193497 a.

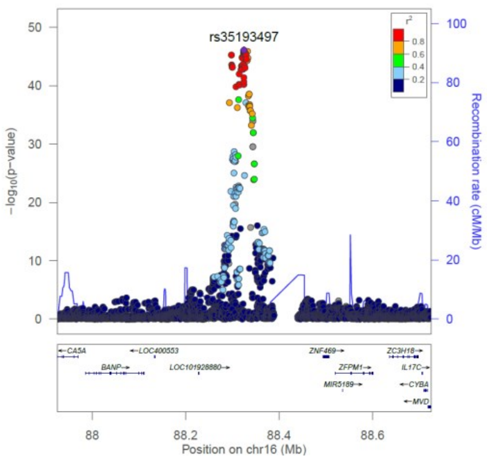

b.

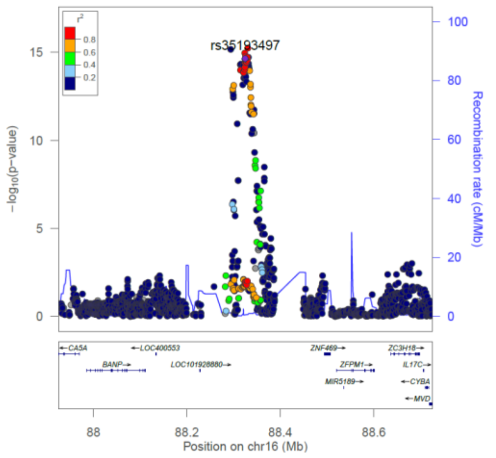

rs4792535 a.

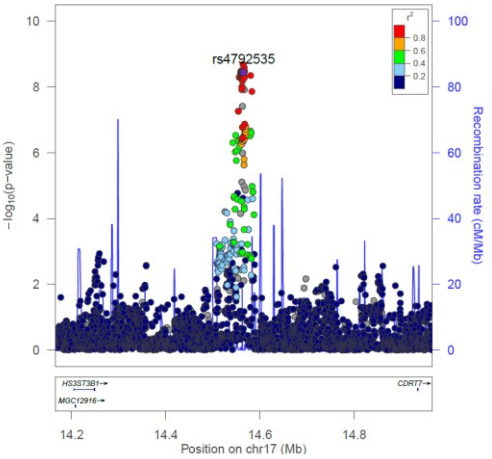

b.

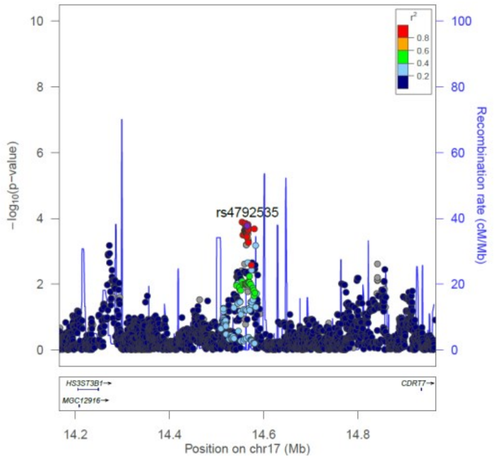

rs8133436 a.

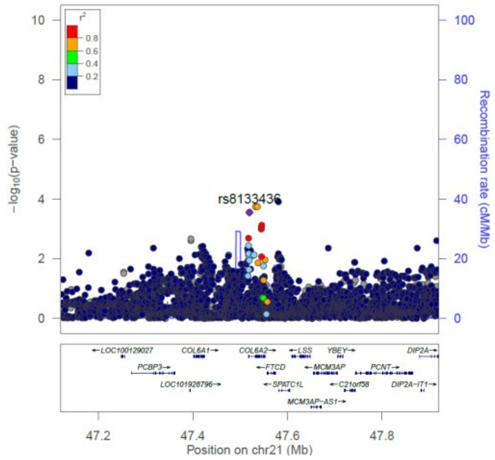

b.

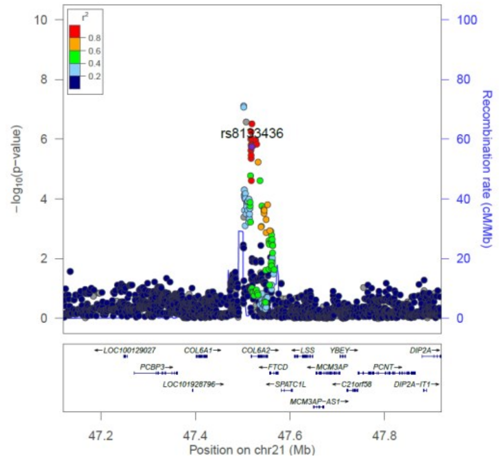

rs71313931 a.

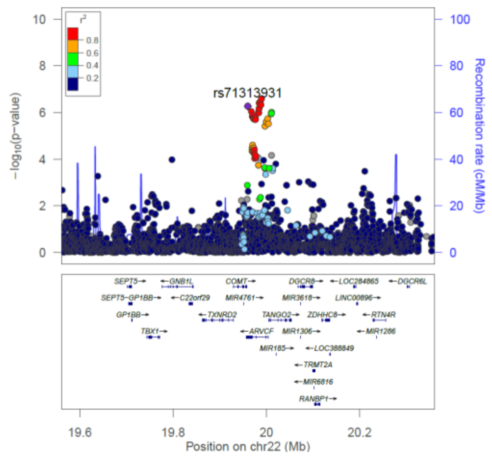

b.

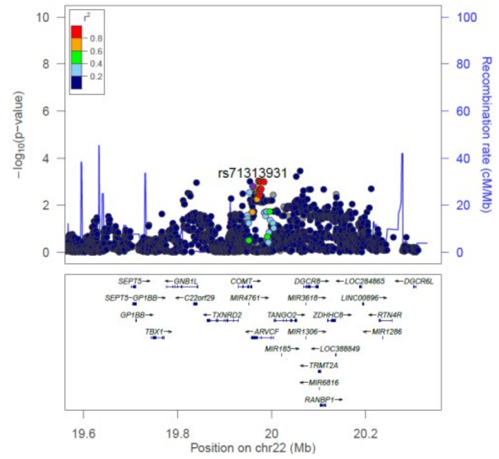

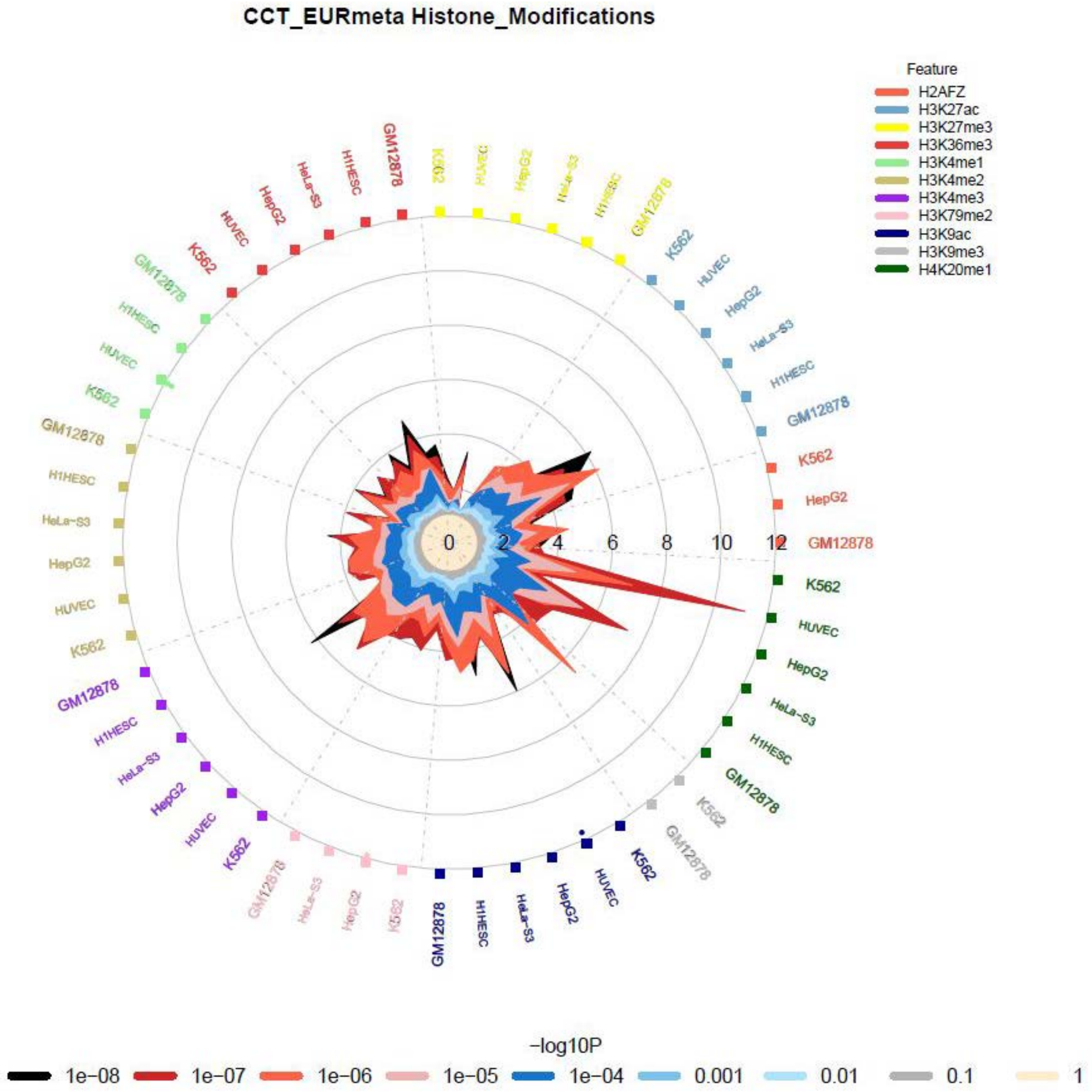

Supplementary Figure 6. Histone modification annotation enrichment amongst CCT associated variants, using European-specific GWAS results

The radial axis represents the fold enrichment in regulatory histone modifications features amongst the CCT European meta-analysis association signals. Eleven histone modification marks based on ENCODE ChIP-Seq data from the human cell-lines legended in the outer circle were available: H2AFZ (marking nucleosomal mobility), H3K27ac (marking active enhancer elements), H3K27me3 (inactive transcription site, poised enhancers), H3K36me3 (facultative and constitutive heterochromatin), H3K4me1(active, primed or poised enhancers), H3K4me2 (Transcription factor binding regions), H3K4me3 (active promoters), H3K79me2 (5’ end of gene

bodies), H3K9ac(active promoters), H3K9me3 (constitutive heterochromatin), H4K20me1(transcriptional activation). Enrichment is plotted for nine GWAS significance thresholds (colour coded as indicated in bottom line of figure based on -log10P-value, *P* being the CCT association *P*-value

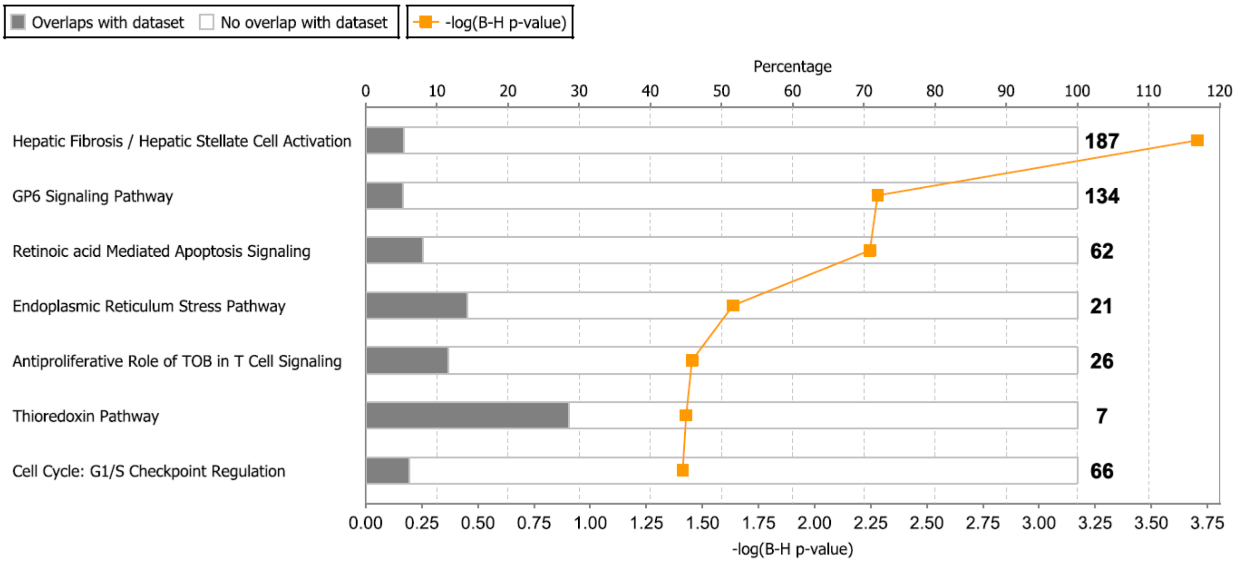

© 2000-2017 QIAGEN. All rights reserved.

Supplementary Figure 7. Canonical pathways pointed by the genes located +/- 200kb of the lead CCT-associated SNPs.

The bar chart shows the canonical pathways found using Ingenuity Pathway Analysis (IPA) software. The *y-axis* (on the left) shows the name of the canonical pathway, while the *y-axis* (on the right) shows the number of genes that belong to each canonical pathway. The *x-axis* (on the top) shows the percentage of genes located in the vicinity (+/- 200kb) of CCT-loci that belong to each canonical pathway, whereas the *x-axis*(on the bottom) shows the -log Benjamini-Hochberg corrected *P*-value (B-H *P*-value). The orange points connected by a line represent the significance level of each canonical pathway. Only significant canonical pathways -log (B-H *P*-value=0.05) are shown in the figure.

Supplementary Note 2

Acknowledgements

Australian & New Zealand Registry of Advanced Glaucoma (ANZRAG)
Support for recruitment of ANZRAG was provided by the Royal Australian and New Zealand College of Ophthalmology (RANZCO) Eye Foundation. Genotyping was funded by the National Health and Medical Research Council of Australia (#535074 and #1023911). The authors acknowledge the support of Ms Bronwyn Usher-Ridge and Ms Emmanuelle Souzeau in patient recruitment and data collection, Professor Matthew A.Brown and Dr Patrick Danoy for genotyping and Mr Rhys Fogarty and Dr Matthew Law for data analysis.

Beijing Eye Study (JJES)
Beijing Eye Study was supported by National Natural Science Foundation of China (grant 81170890).

Blue Mountains Eye Study
BMES was supported by the Australian National Health & Medical Research Council (NH&MRC), Canberra Australia (974159, 211069, 457349, 512423, 475604, 529912); the Centre for Clinical Research Excellence in Translational Clinical Research in Eye Diseases; NH&MRC research fellowships (358702, 632909 to J.J.W, 1028444 to P.N.B.); and the Wellcome Trust, UK as part of Wellcome Trust Case Control Consortium 2 (A. Viswanathan, P. McGuffin, P. Mitchell, F. Topouzis, P. Foster) for genotyping costs of the entire BMES population (085475B08Z, 08547508Z, 076113). The Centre for Eye Research Australia receives Operational Infrastructure Support from the Victorian government. BMES acknowledges Elena Rochtchina from the Centre for Vision Research, Department of Ophthalmology and Westmead Millennium Institute University of Sydney (NSW Australia); John Attia, Rodney Scott, Elizabeth G. Holliday from the University of Newcastle (Newcastle, NSW Australia); Jing Xie, and Andrea J. Richardson from the Centre for Eye Research Australia, University of Melbourne; Michael T. Inouye, Medical Systems Biology, Department of Pathology & Department of Microbiology & Immunology, University of Melbourne (Victoria, Australia); Ananth Viswanathan, Moorfields Eye Hospital (London, UK); Paul J. Foster, NIHR Biomedical Research Centre for Ophthalmology, UCL Institute of Ophthalmology & Moorfields Eye Hospital (London); Peter McGuffin, MRC Social Genetic and Developmental Psychiatry Research Centre, Institute of Psychiatry, King's College (London, United Kingdom); Fotis Topouzis, Department of Ophthalmology, School of Medicine, Aristotle University of Thessaloniki, AHEPA Hospital (Thessaloniki, Greece); Xueling Sim, National University of Singapore.

CROATIA studies

The **CROATIA** studies were funded by grants from the Medical Research Council (UK), from the Republic of Croatia Ministry of Science, Education and Sports (108-1080315-0302; 216-1080315-0302) and the Croatian Science Foundation (8875); and the CROATIA-Korčula

genotyping was funded by the European Union framework program 6 project EUROSPAN (LSHGCT2006018947). The CROATIA studies acknowledges Dr. Biljana Andrijević Derk, Valentina Lacmanović Lončar, Krešimir Mandić, Antonija Mandić, Ivan Škegro, Jasna Pavičić Astaloš, Ivana Merc, Miljenka Martinović, Petra Kralj, Tamara Knežević and Katja Barać-Juretić as well as the recruitment team from the Croatian Centre for Global Health, University of Split and the Institute of Anthropological Research in Zagreb for the ophthalmological data collection; Peter Lichner and the Helmholtz Zentrum Munchen (Munich, Germany), AROS Applied Biotechnology, Aarhus, Denmark and the Wellcome Trust Clinical facility (Edinburgh, United Kingdom) for SNP array genotyping; . Genetic analyses were supported by the MRC HGU "QTL in Health and Disease" core programme.

ORCADES

ORCADES recruitment and genotyping were supported by the Chief Scientist Office of the Scottish Government, the Royal Society, the UK Medical Research Council Human Genetics Unit and the European Union framework program 6 EUROSPAN project (LSHGCT2006018947). ORCADES acknowledges the invaluable contributions of Lorraine Anderson and the research nurses in Orkney, in particular Margaret Pratt who performed the eye measurements, as well as the administrative team in Edinburgh University, the Wellcome Trust Clinical facility (Edinburgh, United Kingdom) for DNA extraction, Peter Lichner and the Helmholtz Zentrum Munchen (Munich, Germany) for genotyping, and Mirna Kirin, Pau Navarro and Peter Joshi for the genetic data imputation. Genetic analyses were supported by the MRC HGU "QTL in Health and Disease" core programme.

Erasmus Rucphen Family (ERF) Study and Rotterdam Study

Erasmus Rucphen family study: The ERF study as a part of EUROSPAN (European Special Populations Research Network) was supported by European Commission FP6 STRP grant number 018947 (LSHG-CT-2006-01947) and also received funding from the European Community's Seventh Framework Programme (FP7/2007-2013)/grant agreement HEALTH-F4-2007-201413 by the European Commission under the program 'Quality of Life and Management of the Living Resources' of the 5th Framework Programme (number QLG2-CT-2002-012.

The Rotterdam Study is funded by Erasmus Medical Center and Erasmus University, Rotterdam, Netherlands Organization for the Health Research and Development (ZonMw), the Research Institute for Diseases in the Elderly (RIDE), the Ministry of Education, Culture and Science, the Ministry for Health, Welfare and Sports, the European Commission (DG XII), and the Municipality of Rotterdam. The generation and management of GWAS genotype data for the Rotterdam Study (RS-I, RS-II, RS-III) was executed by the Human Genotyping Facility of the Genetic Laboratory of the Department of Internal Medicine, Erasmus MC, Rotterdam, The Netherlands. The GWAS datasets are supported by the Netherlands Organization of Scientific Research NWO Investments (nr. 175.010.2005.011, 911-03-012), the Genetic Laboratory of the Department of Internal Medicine, Erasmus MC, the Research Institute for Diseases in the Elderly (014-93-015; RIDE2), the Netherlands Genomics Initiative (NGI)/Netherlands Organization for Scientific Research (NWO) Netherlands Consortium for Healthy Aging (NCHA), project nr. 050-060-810. This study makes use of an extended dataset of RS-II and RS-III samples based on Illumina Omni 2.5 and 5.0 GWAS genotype data. This dataset was funded by the Genetic Laboratory of the

Department of Internal Medicine, the Department of Forensic Molecular Biology, and the Department of Dermatology, Erasmus MC, Rotterdam, The Netherlands. We thank Pascal Arp, Mila Jhamai, Marijn Verkerk, Lizbeth Herrera and Marjolein Peters, MSc, and Carolina Medina-Gomez, PhD, for their help in creating the GWAS database. A.I. Iglesias was supported by the National Institute of Health (NIH), National Eye Institute (NEI), (1 R01 EY024233-03).

Glaucoma Genes and Environment (GLAUGEN) and National Eye Institute (NEI) Glaucoma Human Genetics Collaboration (NEIGHBOR)

Genotyping services for the NEIGHBOR study were provided by the Center for Inherited Disease Research (CIDR) and were supported by the National Eye Institute through grant HG005259-01 (JL Wiggs). Additionally, CIDR is funded through a federal contract from the National Institutes of Health to The Johns Hopkins University, contract number HHSN268200782096C. Genotyping for the GLAUGEN dataset at the Broad Institute was supported by GENEVA project grant HG004728 (LR Pasquale) and U01-HG004424 (Broad Institute). Genotype data cleaning and analysis for the GLAUGEN study was supported by U01 HG004446 (C Laurie). Collecting and processing samples for the NEIGHBOR dataset was supported by the National Eye Institute through ARRA grants 3R01EY015872-05S1 (JL Wiggs) and 3R01EY019126-02S1 (MA Hauser). Funding for the collection of cases and controls was provided by NIH grants: EY015543 (RR Allingham), EY006827 (D Gaasterland); HL73042, HL073389, EY13315 (MA Hauser); CA87969 (JH Kang), CA49449 (JH Kang), CA55075 (JH Kang), EY009149 (PR Lichter), HG004608 (C McCarty), EY008208 (FA Medeiros), EY015473 (LR Pasquale), EY012118 (M Pericak-Vance), EY015682 (A Realini), EY011671 (JE Richards), EY09580 (JE Richards), EY013178 (JS Schuman), RR015574, EY015872 (JL Wiggs), EY010886 (JL Wiggs), EY009847 (JL Wiggs), EY011008, EY144428 (K Zhang), EY144448 (K Zhang), EY18660 (K Zhang). JL Wiggs and LR Pasquale are also supported by the Harvard Glaucoma Center for Excellence and the Margolis Fund. Y Liu is supported by the Glaucoma Research Foundation, American Health Assistance Foundation, and the Glaucoma Foundation. JL Wiggs, LR Pasquale, DC Musch, and JE Richards are supported by Research to Prevent Blindness.

Gutenberg Health Study (GHS I, GHS II)

The Gutenberg Health Study was funded through the government of Rhineland-Palatine ("Stiftung Rheinland Pfalz für Innovation" (AZ961386261733); the research programs "Wissen schafft Zukunft" 67 and "Schwerpunkt Vaskuläre Prävention" of the Johannes Gutenberg-University of Mainz; Boehringer Ingelheim, Ingelheim, Germany; PHILIPS Medical Systems, Hamburg, Germany; National Genome Network "NGFNplus" by the Federal Ministry of Education and Research, Germany (A301GS0833).

Western Australian Pregnancy Cohort (Raine) Study

The authors would like to thank the staff of the Western Australia Pregnancy Cohort (Raine) Study, and the participants and their families. The core management of the Raine Study is funded by The University of Western Australia (UWA), The Telethon Kids Institute, Raine Medical Research Foundation, Women's and Infant's Research Foundation, Curtin University, Murdoch University, Edith Cowan University, and The University of Notre Dame

Australia. The 20-year follow-up of Generation 2 of the Raine Study was funded by Australian National Health and Medical Research Council (NHMRC) project grant 1021105, Lions Eye Institute, the Australian Foundation for the Prevention of Blindness and the Ophthalmic Research Institute of Australia. SY is supported by NHMRC Early Career Fellowship (CJ Martin - Overseas Biomedical Fellowship)

Singapore (SCES, SIMES, SINDI)

We acknowledge the following source of funding support : National Medical Research Council, Singapore (NMRC/TCR/002-SERI/2008 (R626/47/2008TCR), CSA R613/34/2008, NMRC 0796/2003, STaR/0003/2008), the National Research Foundation of Singapore, the Biomedical Research Council, Singapore (BMRC 09/1/35/ 19/616, 08/1/35/19/550, 10/1/35/19/675) and Genome Institute of Singapore (GIS/12-AR2105). The Singapore Tissue Network and the Genome Institute of Singapore, Agency for Science, Technology and Research, Singapore provided services.

Twins Eye Study in Tasmania (TEST) and Brisbane Adolescent Twin Study (BATS)

TEST and BATS (Australian Twins) were supported by an Australian National Health and Medical Research Council (NHMRC) Enabling Grant (2004-2009, 350415, 2005-2007); Clifford Craig Medical Research Trust; Ophthalmic Research Institute of Australia; American Health Assistance Foundation; Peggy and Leslie Cranbourne Foundation; Foundation for Children; Jack Brockhoff Foundation; National Institutes of Health/National Eye Institute (RO1EY01824601 (2007-2010)); Pfizer Australia Senior Research Fellowship (to D.A.M.); and Australian NHMRC Career Development Award (to S.M.). Genotyping was funded by an NHMRC Medical Genomics Grant; US National Institutes of Health/National Eye Institute (1RO1EY018246), Australian sample imputation analyses were carried out on the Genetic Cluster Computer which is financially supported by the Netherlands Scientific Organization (NWO48005003). Australian Twins thanks Nicholas Martin, Scott Gordon, Dale Nyholt, Sarah Medland, Brian McEvoy, Margaret Wright, Anjali Henders, Megan Campbell for ascertaining and processing genotyping data; Jane MacKinnon, Shayne Brown, Lisa Kearns, Jonathan Ruddle, 68 Sandra Staffieri, Olivia Bigault, Colleen Wilkinson, Byoung Sung Chu, Robert Macmillan, Johan Poulsen, Yaling Ma, Julie Barbour for assisting with clinical examinations; and Dr Camilla Day and staff at the Center for Inherited Disease Research.

TwinsUK

TwinsUK received funding from the Wellcome Trust; the European Union MyEuropia Marie Curie Research Training Network; Guide Dogs for the Blind Association; the European Community’s FP7 (HEALTHF22008201865GEFOS); ENGAGE (HEALTHF42007201413); the FP-5 GenomEUtwin Project (QLG2CT200201254); US National Institutes of Health/National Eye Institute (1RO1EY018246); NIH Center for Inherited Disease Research; the National Institute for Health Research comprehensive Biomedical Research Centre award to Guy’s and St. Thomas’ National Health Service Foundation Trust partnering with King’s College London. A.N. received funding from Fight for Sight and The Worshipful Company of Spectacle Makers. P.G.H. is the recipient of a Fight for Sight ECI award. C.J.H. is an NIHR

Senior Research fellow. We acknowledge the contribution of Drs Toby Andrew, Margarida Lopes, Samantha Fahy and Diana Kozareva.

REFERENCES

1. Lu, Y. *et al.* Genome-wide association analyses identify multiple loci associated with central corneal thickness and keratoconus. *Nat Genet* **45**, 155-63 (2013).

2. Wang, Y., Rabinowitz, Y.S., Rotter, J.I. & Yang, H. Genetic epidemiological study of keratoconus: evidence for major gene determination. *Am J Med Genet* **93**, 403-9 (2000).

3. Psaty, B.M. *et al.* Cohorts for Heart and Aging Research in Genomic Epidemiology (CHARGE) Consortium: Design of prospective meta-analyses of genome-wide association studies from 5 cohorts. *Circ Cardiovasc Genet* **2**, 73-80 (2009).

4. Burdon, K.P. *et al.* Genome-wide association study identifies susceptibility loci for open angle glaucoma at TMCO1 and CDKN2B-AS1. *Nat Genet* **43**, 574-8 (2011).

5. Bailey, J.N. *et al.* Genome-wide association analysis identifies TXNRD2, ATXN2 and FOXC1 as susceptibility loci for primary open-angle glaucoma. *Nat Genet* **48**, 189-94 (2016).

6. Feuer, W.J. *et al.* The Ocular Hypertension Treatment Study: reproducibility of cup/disk ratio measurements over time at an optic disc reading center. *Am J Ophthalmol* **133**, 19-28 (2002).

7. Yang, J. *et al.* Conditional and joint multiple-SNP analysis of GWAS summary statistics identifies additional variants influencing complex traits. *Nat Genet* **44**, 369-75, S1-3 (2012).

8. Yang, J., Lee, S.H., Goddard, M.E. & Visscher, P.M. GCTA: a tool for genome-wide complex trait analysis. *Am J Hum Genet* **88**, 76-82 (2011).

9. Howie, B., Fuchsberger, C., Stephens, M., Marchini, J. & Abecasis, G.R. Fast and accurate genotype imputation in genome-wide association studies through pre-phasing. *Nat Genet* **44**, 955-9 (2012).

10. Springelkamp, H. *et al.* ARHGEF12 influences the risk of glaucoma by increasing intraocular pressure. *Hum Mol Genet* **24**, 2689-99 (2015).

11. Geer, L.Y. *et al.* The NCBI BioSystems database. *Nucleic Acids Res* **38**, D492-6 (2010).
