# Supplementary material for: Cross-ancestry genome-wide association analysis of corneal thickness strengthens link between complex and Mendelian eye diseases
Source: Nat Commun. 2018 May 14;9:1864. doi: 10.1038/s41467-018-03646-6 (PMC5951816; doi:10.1038/s41467-018-03646-6)
Supplement: Supplementary file 3 — Description of Additional Supplementary Files [file 41467_2018_3646_MOESM3_ESM.pdf]

Description of Additional Supplementary Files

File Name: Supplementary Data 1
Description: The genes not identified through single variant GWAS tests are highlighted.

File Name: Supplementary Data 2
Description: OMIM genes and associated Mendelian disease, with transcription start site within 1Mb of the CCT associated variant.

File Name: Supplementary Data 3
Description: Table shows annotation for the 54 lead variants (in bold) and those in high LD with maximum or medium evidence of being regulatory variants according to their Regulome DB score.

File Name: Supplementary Data 4
Description: Top 30 biosystems gene-set or pathways observed through pathway analysis of gene-based pvalues obtained using VEGAS2, using a 10kb window. In following table, the gene-set or pathway ID comprises the BiosystemID_SourceDatabaseID_SourceDatabaseTerm.

File Name: Supplementary Data 5
Description: Top 30 biosystems gene-set or pathways observed through pathway analysis of gene-based pvalues obtained using VEGAS2, using a 200kb window. In following table, the gene-set or pathway ID comprises the BiosystemID_SourceDatabaseID_SourceDatabaseTerm.
